# Hypervalent iodine-mediated Ritter-type amidation of terminal alkenes: The synthesis of isoxazoline and pyrazoline cores

**DOI:** 10.3762/bjoc.14.89

**Published:** 2018-05-11

**Authors:** Sang Won Park, Soong-Hyun Kim, Jaeyoung Song, Ga Young Park, Darong Kim, Tae-Gyu Nam, Ki Bum Hong

**Affiliations:** 1 Department of Pharmacy and Institute of Pharmaceutical Science and Technology, Hanyang University, Ansan, Gyeonggi-do 15588, Republic of Korea; 2 New Drug Development Center (NDDC), Daegu-Gyeongbuk Medical Innovation Foundation (DGMIF), 80 Cheombok-ro, Dong-gu, Daegu, Republic of Korea

**Keywords:** amido-amidation, hypervalent iodine, isoxazoline, metal-free, oxyamidation, pyrazoline

## Abstract

Hypervalent iodine-mediated olefin functionalization provides a rapid gateway towards accessing both various heterocyclic cores and functional groups. In this regard, we have developed a Ritter-type alkene functionalization utilizing a PhI(OAc)_2_ ((diacetoxyiodo)benzene, PIDA)/Lewis acid combination in order to access isoxazoline and pyrazoline cores. Based on allyl ketone oximes and allyl ketone tosylhydrazones, we have developed an alkene oxyamidation and amido-amidation protocol en route to accessing both isoxazoline and pyrazoline cores. Additionally, acetonitrile serves as both the solvent and an amine source in the presence of this PIDA/Lewis acid combination. This operationally straightforward and metal-free protocol provides an easy access to isoxazoline and pyrazoline derivatives.

## Introduction

Isoxazoline and pyrazoline-containing heterocycles are abundant in natural products and biologically active molecules [1–5]. Thus, these scaffolds are also important from the standpoint of pharmaceutical and medicinal chemistry [6–11]. Not surprisingly, the construction of diverse heterocyclic cores including isoxazolines and pyrazolines has garnered much attention from synthetic chemists [12–15]. Among precedent synthetic methods, the functionalization of unactivated olefins provides a rapid construction of different heterocycles [16–17]. More specifically, the formation of isoxazoline and pyrazoline cores via alkene heterofunctionalization of allyl ketone oximes and/or allyl ketone tosylhydrazones has been well documented [18–22]. For example, diverse halonium sources have been utilized for the synthesis of isoxazolines via halocyclization. Furthermore, transition metal-, visible light, and hypervalent iodine-mediated oxidative cyclization protocols provide isoxazoline backbones bearing diverse substituents such as –SR, -CF_3_, -OH and halogens [23–27].

## Results and Discussion

**Scheme 1:**
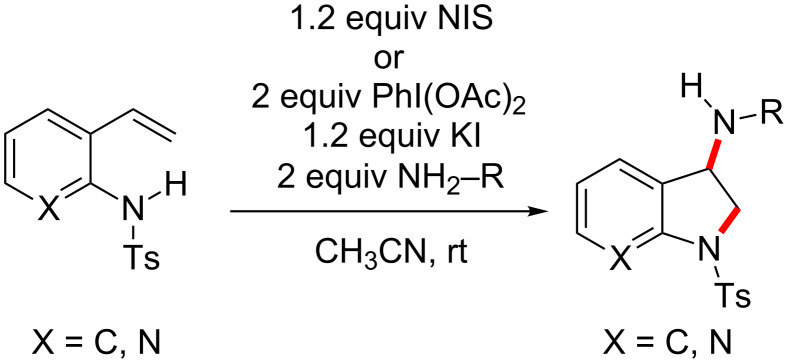
Hypervalent iodine-mediated heterofunctionalization of terminal alkenes.

As depicted in Scheme 1, we have previously reported an inter-/intramolecular alkene diamination using either *N*-iodosuccinimide (NIS) or a phenyliodine diacetate (PIDA)/halide additive combination [28–30]. Vinylanilines and vinylaminopyridines in combination with electron-rich, Brønsted basic amines were converted to their corresponding indoline and azaindoline derivatives.

However, an attempted expansion of this methodology to allyl ketone oximes and allyl ketone tosylhydrazones proved unsuccessful under the previously reported reaction conditions (see Supporting Information File 1, Table S1). Upon optimization with various oxidants and additives screened, it was found that a Lewis acid additive can promote the olefin heterofunctionalization via a Ritter-type amidation using acetonitrile as both the solvent and the amine source. Interestingly, this hypervalent iodine-mediated Ritter-type oxyamidation of **1a** proved less efficient in the presence of solvent combinations with acetonitrile, despite acetonitrile being used in vast excess (see Supporting Information File 1, Table S1). Herein, we entail the first example of a hypervalent iodine(III)-mediated Ritter-type oxyamidation and amido-amidation of terminal alkenes in the presence of a Lewis acid.

Optimization studies of this Ritter-type oxyamidation commenced with oxidant screening in the presence of a Lewis acid (Table 1). Without oxidant, the Ritter-type oxyamidation still proceeded to give **3a** albeit in low yield (Table 1, entry 1). The background reaction mediated by a Lewis acid seemed plausible via an electrophilic activation of the double bond. When the reaction is performed in the presence of hypervalent iodine reagents such as PIFA ([bis(trifluoroacetoxy)iodo]benzene), PhI(NPhth)_2_ and PIDP (bis(*tert*-butylcarbonyloxy)iodobenzene) much better yields were obtained (Table 1, entries 2–4), with PhI(OAc)_2_ proving to be the best (Table 1, entry 5). Refluxing conditions further improved the yield (Table 1, entry 6). Additionally, other cyclic hypervalent iodine oxidants such as IBX (2-iodoxybenzoic acid) and DMP (Dess–Martin periodinane) (Table 1, entries 7 and 8) gave similar yields to the background reaction. Lastly, a Lewis acid screen (Table 1, entries 9–12) was performed. Among the tested Lewis acids, AlCl_3_, SnCl_4_, TiCl_4_, TMSOTf and BF_3_·Et_2_O, the latter was found to be the best choice of additive. Remarkably, the activation of PIDA by a Lewis acid (BF_3_·OEt_2_) seemed to be crucial for this Ritter-type oxyamidation to proceed (Table 1, entry 13). Based on these experiments, we chose the conditions outlined in Table 1, entry 5 for our further investigations due to the mild (room temperature) reaction conditions.

**Table 1:** Hypervalent iodine-mediated Ritter-type alkene oxyamidation.

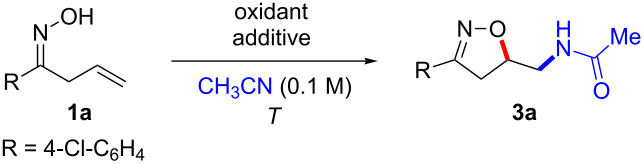				

entry^a^	oxidant (equiv)	additive (equiv)	*T* (°C)	yield of **3a** (%)^b^

1	–	BF_3_·OEt_2_ (1.0)	25	10
2	PhI(OCOCF_3_)_2_ (1.0)	BF_3_·OEt_2_ (1.0)	25	35
3	PhI(NPhth)_2_ (1.0)	BF_3_·OEt_2_ (1.0)	25	42
4	PIDP (1.0)	BF_3_·OEt_2_ (1.0)	25	49
5	PhI(OAc)_2_ (1.0)	BF_3_·OEt_2_ (1.0)	25	55
6	PhI(OAc)_2_ (1.0)	BF_3_·OEt_2_ (1.0)	reflux	60
7	IBX (1.0)	BF_3_·OEt_2_ (1.0)	25	14
8	DMP (1.0)	BF_3_·OEt_2_ (1.0)	25	14
9	PhI(OAc)_2_ (1.0)	AlCl_3_ (1.0)	25	0
10	PhI(OAc)_2_ (1.0)	SnCl_4_ (1.0)	25	0
11	PhI(OAc)_2_ (1.0)	TiCl_4_ (1.0)	25	12
12	PhI(OAc)_2_ (1.0)	TMSOTf (1.0)	25	45
13	PhI(OAc)_2_ (1.0)	–	25	0

^a^All reactions were performed on a 0.21 mmol scale (0.1 M) and with a standard 18 h reaction time. ^b^Isolated yield.

**Scheme 2:**
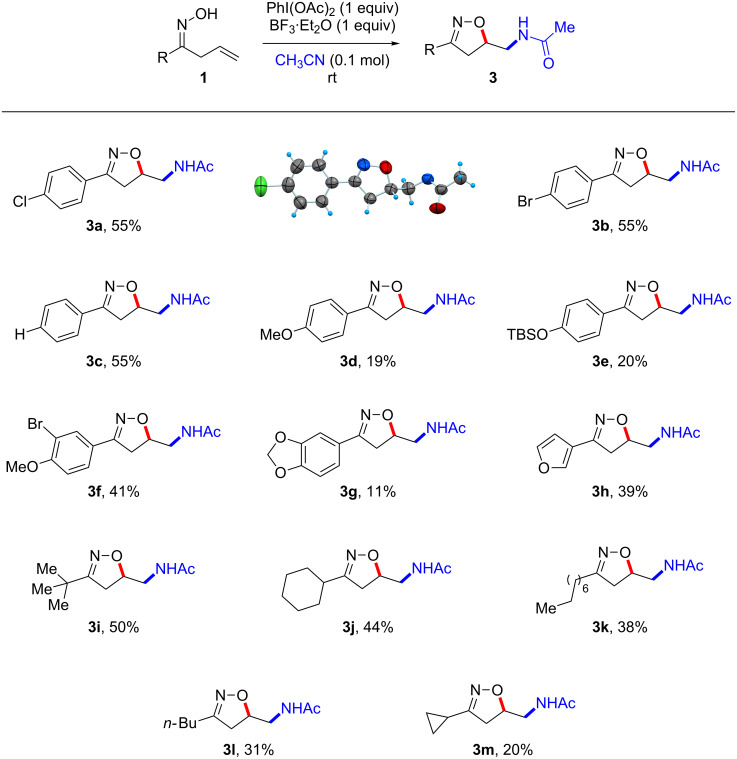
Substrate scope of the Ritter-type oxyamidation: isoxazoline synthesis. All reactions were performed on a 0.21 mmol scale (0.1 M) and with a standard 18 h reaction time.

Next a series of allyl ketone oximes **1** were subjected to the optimized reaction conditions and the results are summarized in Scheme 2. Phenyl and electron-deficient aryl allyl ketone oximes showed robust reactivity as the corresponding products were obtained in good yields (**3a**–**c**, **3f**) [31]. However, electron-rich aryl allyl ketone oximes such as **1d**, **1e** and **1g** proved inferior. Also the furan-substituted allyl ketone oxime delivered the desired product **3h** albeit in a moderate yield. In addition, various alkyl allyl ketone oximes were investigated. While cyclopropyl allyl ketone oxime **1m** was converted to the corresponding isoxazoline **3m** in 20% yield, other alkyl allyl ketone oximes afforded higher yields of the desired products. This observation is ascribed differences in reactivity due to an increased steric bulk at the α-position (*t*-Bu > *c*-Hex > *n*-Oct > *n*-Bu).

**Scheme 3:**
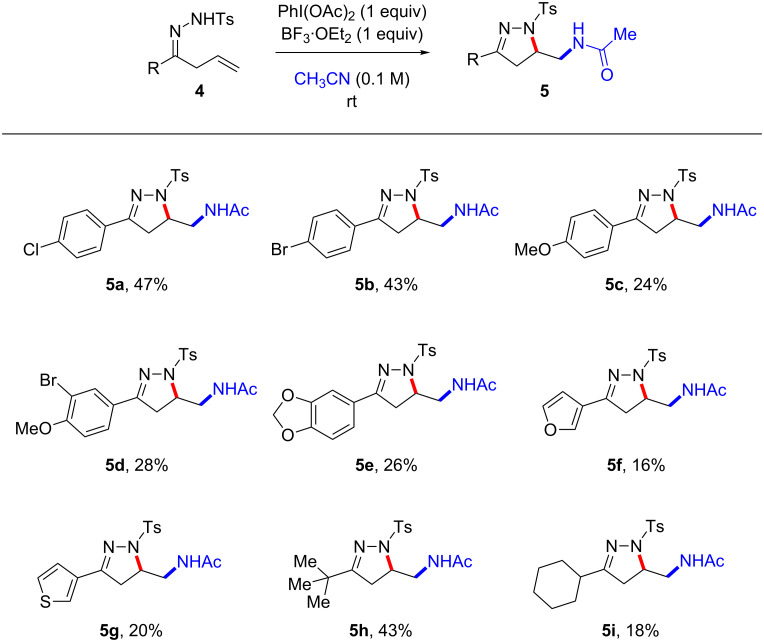
Substrate scope of Ritter-type amido-amidation: pyrazoline synthesis. All reactions were performed on a 0.21 mmol scale (0.1 M) and with a standard 18 h reaction time.

We next explored the hypervalent iodine-mediated Ritter-type amido-amidation reaction in the presence of a Lewis acid in order to access pyrazoline cores (Scheme 3). A series of allyl ketone tosylhydrazones **4** were subjected to the same reaction conditions. In general, the yields of the pyrazoline cores decreased marginally relative to the Ritter-type oxyamidation reaction. Monosubstituted aryl allyl ketone tosylhydrazones showed good reactivity and provided the pyrazoline heterocycles **5a**–**c** in moderate yields (24–47%). On the other hand, disubstituted aryl allyl ketone tosylhydrazones **4d** and **4e** yielded the corresponding products in 28% and 26%, respectively. The reaction of heteroaryl allyl ketone tosylhydrazones such as 3-furyl allyl ketone tosylhydrazone **4f** and 3-thiophenyl allyl ketone tosylhydrazone **4g** provided their desired products in low yields. Lastly, alkyl allyl ketone tosylhydrazones **4h** and 4**i** seemed to maintain of the trend in which increased reactivity is the result of increasing size of the alkyl side chain at the α-position (*t*-Bu > *c*-Hex).

**Scheme 4:**
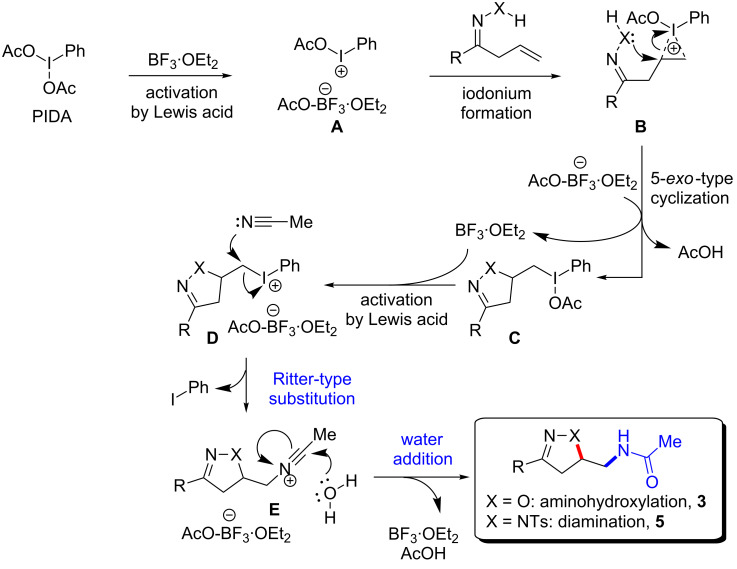
Plausible mechanism of the hypervalent iodine(III)-mediated Ritter-type oxyamidation/amido-amidation in the presence of a Lewis acid.

Based on these experimental data and previous reports [32–33], a plausible mechanism of the Ritter-type oxyamidation and amido-amidation is proposed in Scheme 4. First, an activation of hypervalent iodine(III) by the Lewis acid generates the active iodine(III) species **A** in situ. The resulting iodine(III) then, in turn, forms the electrophilic iodonium intermediate **B** with the terminal alkene of the allyl ketone oxime or allyl ketone tosylhydrazone. The subsequent 5-*exo*-type cyclization by nucleophilic attack on the iodonium then leads to the isoxazoline or pyrazoline cores (**C**) bearing the hypervalent iodine(III) group. Following iodine activation by the Lewis acid, the iodonium ion **D** undergoes nucleophilic substitution with excess acetonitrile to form intermediate **E**. Then water can add to the corresponding nitrilium and subsequent tautomerization delivers isoxazoline **3** (X = O) and pyrazoline **5** (X = NTs) via a Ritter-type oxyamidation and amido-amidation.

## Conclusion

In summary, we have developed a hypervalent iodine(III)-mediated inter-/intramolecular Ritter-type oxyamidation and amido-amidation protocol in the presence of a Lewis acid. This transformation provides direct access to diverse 5-acetaminomethyl substituted 2-isoxazoline/2-pyrazoline derivatives using acetonitrile as both the solvent and amine source.

## Supporting Information

File 1 Experimental procedures, characterization data, and copies of ^1^H and ^13^C NMR spectra.
