# Supplementary material for: Hypervalent iodine-mediated Ritter-type amidation of terminal alkenes: The synthesis of isoxazoline and pyrazoline cores
Source: Beilstein J Org Chem. 2018 May 11;14:1028–33. doi: 10.3762/bjoc.14.89 (PMC6009250; doi:10.3762/bjoc.14.89)

Supporting Information

for

Hypervalent iodine-mediated Ritter-type amidation of terminal alkenes: The synthesis of isoxazoline and pyrazoline cores

Sang Won Park[§,1], Soong-Hyun Kim[§,2], Jaeyoung Song[2], Ga Young Park[2], Darong Kim[2], Tae-Gyu Nam[*,1], and Ki Bum Hong[*,2]

Address: [1]Department of Pharmacy and Institute of Pharmaceutical Science and Technology, Hanyang University, Ansan, Gyeonggi-do 15588, Republic of Korea and [2]New Drug Development Center (NDDC), Daegu-Gyeongbuk Medical Innovation Foundation (DGMIF), 80 Cheombok-ro, Dong-gu, Daegu, Republic of Korea

Email: Tae-Gyu Nam - tnam@hanyang.ac.kr; Ki Bum Hong - kbhong@dgmif.re.kr

*Corresponding author

[§]Equally contributing authors

Experimental procedures, characterization data, and copies of ^{1}H and ^{13}C NMR spectra

Table S1: Hypervalent iodine-mediated Ritter-type alkene oxyamidation.

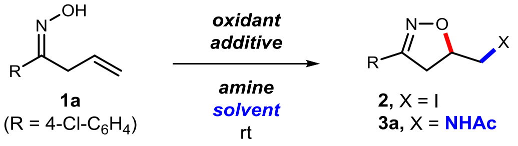

entry[a]	oxidant (equiv.)	additive (equiv.)	amine (equiv.)	solvent (0.1M)	yield of 2/3a (%)[b]
1	NIS (1.2)	BF_3•OEt_2 (1.0)	$BnNH_2$ (1.0)	CH_3CN	80 / 0
2	$PhI(OAc)_2$ (1.0)	BF_3•OEt_2 (1.0), I_2 (1.2)	$BnNH_2$ (1.0)	CH_3CN	77 / 0
3	$PhI(OAc)_2$ (1.0)	BF_3•OEt_2 (1.0), KI (1.0)	$BnNH_2$ (1.0)	CH_3CN	10 / 46
4	$PhI(OAc)_2$ (1.0)	BF_3•OEt_2 (1.0)	$BnNH_2$ (1.0)	CH_3CN	0 / 55
5	$PhI(NPhth)_2$ (1.0)	BF_3•OEt_2 (1.0)	$BnNH_2$ (1.0)	CH_3CN	0 / <5
6	$PhI(OAc)_2$ (1.0)	BF_3•OEt_2 (1.0)	-	CH_3CN	0 / 55
7		BF_3•OEt_2 (1.0)		CH_3CN	0 / 10
8	$PhI(OAc)_2$ (1.0)	BF_3•OEt_2 (1.0)	-	THF	0 / 0
9	$PhI(OAc)_2$ (1.0)	BF_3•OEt_2 (1.0)	-	MeOH	0 / 0
10	$PhI(NPhth)_2$ (1.0)	$AlCl_3$ (1.0)	-	CH_3CN	0 / 0
11	$PhI(NPhth)_2$ (1.0)	$TiCl_4$ (1.0)	-	CH_3CN	0 / 0
12	$PhI(NPhth)_2$ (1.0)	$SnCl_4$ (1.0)	-	CH_3CN	0 / 0
13	$PhI(NPhth)_2$ (1.0)	TMSOTf (1.0)	-	CH_3CN	0 / <5
14	$PhI(OAc)_2$ (1.0)	BF_3•OEt_2 (1.0)	-	Toluene	0 / 0
15	$PhI(OAc)_2$ (1.0)	BF_3•OEt_2 (1.0)	CH_3CN (5)	Toluene	0 / <5
16	$PhI(OAc)_2$ (1.0)	BF_3•OEt_2 (1.0)	CH_3CN (10)	Toluene	0 / <5
17	$PhI(OAc)_2$ (1.0)	BF_3•OEt_2 (1.0)	CH_3CN (100)	Toluene	0 / <5
18	$PhI(NPhth)_2$ (1.0)	BF_3•OEt_2 (1.0)	4-Methoxybenzonitrile (20)	Toluene	0 / 0
19	$PhI(NPhth)_2$ (1.0)	BF_3•OEt_2 (1.0)	4-Methoxybenzonitrile (20)	CH_3CN	0 / <5
20	$PhI(NPhth)_2$ (1.0)	BF_3•OEt_2 (1.0)	Terephthalonitrile (20)	Toluene	0 / 0
21	$PhI(NPhth)_2$ (1.0)	BF_3•OEt_2 (1.0)	Terephthalonitrile (20)	CH_3CN	0 / <5

[a]All reactions were performed on a 0.21 mmol scale (0.1 M) and a standard 18 h reaction time. [b]Isolated yield.

Experimental section

Additional experiments

Unless noted otherwise, materials were purchased from commercial suppliers and used without further purification. Air or moisture-sensitive reactions were carried out under an inert gas atmosphere. Progress of reactions was monitored by thin layer chromatography (TLC) using silica gel F_{254} plates. Purification of the products was performed by flash column chromatography using silica gel 60 (70–230 mesh) or by Biotage 'Isolera One' system with the indicated solvents. Melting points were determined using a Kruss melting pointer meter and were not corrected. NMR spectra were obtained using a Bruker spectrometer operating at 400 MHz or 600 MHz for ^1H NMR, and 100 MHz or 150 MHz for ^{13}C NMR, respectively. Chemical shifts (δ) are expressed in ppm using residual undeuterated solvent as an internal standard and coupling constants (*J*) are reported in hertz. Low-resolution mass spectra (LRMS) were obtained using an

Advion Expression CMS in the positive ion mode with an electrospray (ESI) source. High-resolution mass spectra (HRMS) were obtained using a Thermo Scientific LTQ Orbitrap XL mass spectrometer in the positive ion mode with an electrospray (ESI) source.

Hypervalent iodine-mediated intra-/intermolecular aminohydroxylation

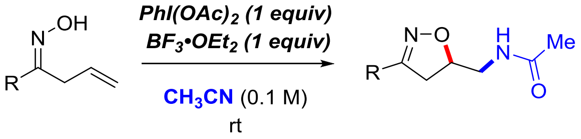

General procedure for isoxazoline formation: To a stirred solution of the corresponding oximes (1 equiv) in MeCN (0.1 M) were added $PhI(OAc)_2$ (1 equiv) followed by $BF_3{\cdot}OEt_2$ (1 equiv) at room temperature. After 18 h, the reaction mixture was quenched with 1 N aqueous solution of sodium thiosulfate (1 mL), dried over $MgSO_4$ and concentrated in vacuo. The obtained residue was purified using flash column chromatography (SiO_2, MeOH in CH_2Cl_2) to afford the corresponding isoxazolines.

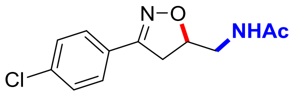

***N*-((3-(4-Chlorophenyl)-4,5-dihydroisoxazol-5-yl)methyl)acetamide (3a).** Prepared according to the general procedure using corresponding oxime (50 mg, 0.26 mmol). Flash column chromatography (SiO_2, 1–2% MeOH in CH_2Cl_2) yielded a white solid (36 mg, 55%). R_f = 0.20 (6% MeOH in CH_2Cl_2); m.p. : 185~189 °C; ^{1}H NMR (400 MHz, DMSO-d_6) δ 8.14 (t, J = 5.7 Hz, 1H), 7.69–7.63 (m, 2H), 7.55–7.49 (m, 2H), 4.74 (ddt, J = 10.9, 7.1, 5.6 Hz, 1H), 3.45 (dd, J = 17.1, 10.7 Hz, 1H), 3.25 (t, J = 5.8 Hz, 2H), 3.11 (dd, J = 17.2, 7.2 Hz, 1H), 1.81 (s, 3H) ppm; ^{13}C NMR (100 MHz, $CDCl_3$) δ 170.9, 156.5, 136.5, 129.3, 128.1, 127.8, 80.3, 42.5, 37.6, 23.4 ppm; MS (ESI) *m/z* $[M+Na]^+$ 273.1; HRMS (ESI): Exact mass calcd for $C_{12}H_{13}ClN_2O_2$ $[M+H]^+$ 253.0735, found 253.0738.

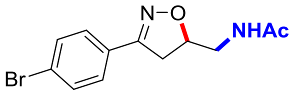

***N*-((3-(4-Bromophenyl)-4,5-dihydroisoxazol-5-yl)methyl)acetamide (3b).** Prepared according to the general procedure using corresponding oxime (185 mg, 0.77 mmol). Flash column chromatography (SiO_2, 1–2% MeOH in CH_2Cl_2) yielded a white solid (125 mg, 55%). R_f = 0.20 (5% MeOH in CH_2Cl_2); m.p. : 198~200 °C; ^{1}H NMR (600 MHz, DMSO-d_6) δ 8.14 (t, J = 5.8 Hz, 1H), 7.68 – 7.64 (m, 2H), 7.61 – 7.57 (m, 2H), 4.78 – 4.72 (m, 1H), 3.44 (dd, J = 17.1, 10.7 Hz, 1H), 3.26 (t, J = 5.6 Hz, 2H), 3.11 (dd, J = 17.1, 7.2 Hz, 1H), 1.82 (s, 3H) ppm; ^{13}C NMR (150 MHz, DMSO-d_6) δ 170.1,

156.4, 132.2, 129.1, 129.0, 123.8, 80.2, 42.3, 37.6, 23.0 ppm; MS (ESI) *m/z* $[M+Na]^+$ 317.0, 319.0; HRMS (ESI): Exact mass calcd for $C_{12}H_{13}BrN_2O_2$ $[M+H]^+$ 297.0237, found 297.0233.

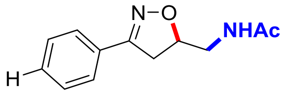

***N*-((3-Phenyl-4,5-dihydroisoxazol-5-yl)methyl)acetamide (3c).** Prepared according to the general procedure using corresponding oxime (81 mg, 0.50 mmol). Flash column chromatography (SiO_2, 1% MeOH in CH_2Cl_2) yielded a white solid (60 mg, 55%). R_f = 0.20 (5% MeOH in CH_2Cl_2); m.p. : 157~160 °C; ^{1}H NMR (400 MHz, DMSO-d_6) δ 8.15 (t, *J* = 5.7 Hz, 1H), 7.65 (ddd, *J* = 4.0, 3.0, 1.5 Hz, 2H), 7.47–7.43 (m, 3H), 4.73 (ddt, *J* = 10.9, 7.1, 5.6 Hz, 1H), 3.46 (dd, *J* = 17.1, 10.6 Hz, 1H), 3.26 (t, *J* = 5.8 Hz, 2H), 3.17–3.08 (m, 1H), 1.82 (s, 3H) ppm; ^{13}C NMR (100 MHz, $CDCl_3$) δ 170.9, 157.2, 130.3, 129.1, 128.8, 126.7, 79.8, 42.4, 37.6, 23.2 ppm; MS (ESI) *m/z* $[M+Na]^+$ 239.5; HRMS (ESI): Exact mass calcd for $C_{12}H_{14}N_2O_2$ $[M+H]^+$ 219.1128, found 219.1128.

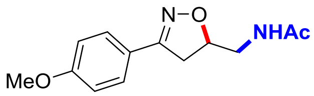

***N*-((3-(4-Methoxyphenyl)-4,5-dihydroisoxazol-5-yl)methyl)acetamide (3d).** Prepared according to the general procedure using corresponding oxime (96 mg, 0.50 mmol). Flash column chromatography (SiO_2, 1–3% MeOH in CH_2Cl_2) yielded a brown solid (23 mg, 19%). R_f = 0.25 (5% MeOH in CH_2Cl_2); m.p. : 180~184 °C; ^{1}H NMR (400 MHz, $CDCl_3$) δ 7.61 – 7.55 (m, 2H), 6.94 – 6.89 (m, 2H), 6.04 (s, 1H), 4.87 – 4.78 (m, 1H), 3.83 (s, 3H), 3.60 (ddd, *J* = 14.3, 5.8, 3.2 Hz, 1H), 3.49 (dt, *J* = 14.2, 6.1 Hz, 1H), 3.37 (dd, *J* = 16.8, 10.5 Hz, 1H), 3.07 (dd, *J* = 16.8, 7.3 Hz, 1H), 1.98 (s, 3H) ppm; ^{13}C NMR (100 MHz, $CDCl_3$) δ 170.7, 161.3, 156.8, 128.3, 1216, 114.2, 79.5, 55.4, 42.4, 37.8, 23.2 ppm; MS (ESI) *m/z* 269.2 $[M+H]^+$; HRMS (ESI): Exact mass calcd for $C_{13}H_{16}N_2O_3$ $[M+H]^+$ 249.1236, found 249.1234

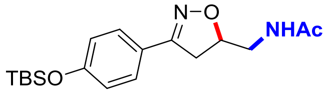

***N*-((3-(4-((*tert*-Butyldimethylsilyl)oxy)phenyl)-4,5-dihydroisoxazol-5-yl)methyl)acetamide (3e).** Prepared according to the general procedure using corresponding oxime (188 mg, 0.65 mmol). Flash column chromatography (SiO_2, 1–2% MeOH in CH_2Cl_2) yielded a brown liquid (43 mg, 20%). R_f = 0.20 (5% MeOH in CH_2Cl_2); ^{1}H NMR (600 MHz, $CDCl_3$) δ 7.52 (d, *J* = 8.6 Hz, 2H), 6.84 (d, *J* = 8.6 Hz, 2H), 6.16 (s, 1H), 4.87 – 4.76 (m, 1H), 3.59 (ddd, *J* = 14.2, 5.7, 3.1 Hz, 1H), 3.51 – 3.44 (m, 1H), 3.36 (dd, *J* = 16.7, 10.5 Hz, 1H), 3.06 (dd, *J* = 16.8, 7.3 Hz, 1H), 1.98 (s, 3H), 0.97 (s, 9H), 0.20 (s, 6H) ppm; ^{13}C NMR (150 MHz, $CDCl_3$) δ 171.1,

157.9, 157.0, 128.4, 122.4, 120.6, 79.6, 42.6, 38.0, 25.8, 23.4, 18.3, -4.2 ppm; MS (ESI) *m/z* 369.1 [M+Na]$^+$; HRMS (ESI): Exact mass calcd for $C_{18}H_{28}N_2O_3Si$ [M+H]$^+$ 349.1947, found 349.1942.

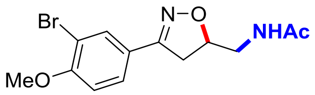

***N*-((3-(3-Bromo-4-methoxyphenyl)-4,5-dihydroisoxazol-5-yl)methyl)acetamide (3f).** Prepared according to the general procedure using corresponding oxime (216 mg, 0.80 mmol). Flash column chromatography (SiO_2, 1–2% MeOH in CH_2Cl_2) yielded a light yellow solid (108 mg, 41%). R_f = 0.20 (5% MeOH in CH_2Cl_2); m.p. : 122~126 ^{o}C; ^{1}H NMR (600 MHz, $CDCl_3$) δ 7.81 (s, 1H), 7.51 (d, *J* = 8.1 Hz, 1H), 6.87 (d, *J* = 8.4 Hz, 1H), 6.25 (s, 1H), 4.83 (s, 1H), 3.90 (s, 3H), 3.57 (d, *J* = 13.1 Hz, 1H), 3.52 – 3.46 (m, 1H), 3.33 (dd, *J* = 16.4, 10.5 Hz, 1H), 3.04 (dd, *J* = 16.5, 6.9 Hz, 1H), 1.98 (s, 3H) ppm; ^{13}C NMR (100 MHz, $CDCl_3$) δ 170.9, 157.3, 155.7, 131.6, 127.2, 122.9, 112.0, 111.7, 79.9, 56.4, 42.3, 37.6, 23.2 ppm; MS (ESI) *m/z* [M+Na]$^+$ 347.3, 349.3; HRMS (ESI): Exact mass calcd for $C_{13}H_{15}BrN_2O_3$ [M+H]$^+$ 327.0344, found 327.0339.

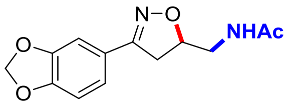

***N*-((3-(Benzo[*d*][1,3]dioxol-5-yl)-4,5-dihydroisoxazol-5-yl)methyl)acetamide (3g).** Prepared according to the general procedure using corresponding oxime (103 mg, 0.50 mmol). Flash column chromatography (SiO_2, 0.5–2% MeOH in CH_2Cl_2) yielded a light yellow solid (14 mg, 11%). R_f = 0.20 (5% MeOH in CH_2Cl_2); m.p. : 154~158 ^{o}C; ^{1}H NMR (600 MHz, $CDCl_3$) δ 7.24 (d, *J* = 1.5 Hz, 1H), 7.01 (dd, *J* = 8.1, 1.6 Hz, 1H), 6.81 (d, *J* = 8.1 Hz, 1H), 6.05 (s, 1H), 6.01 (s, 2H), 4.87 – 4.78 (m, 1H), 3.62 – 3.57 (m, 1H), 3.52 – 3.46 (m, 1H), 3.35 (dd, *J* = 16.7, 10.6 Hz, 1H), 3.05 (dd, *J* = 16.7, 7.3 Hz, 1H), 2.00 (s, 3H) ppm; ^{13}C NMR (100 MHz, $CDCl_3$) δ 170.7, 156.8, 149.5, 148.2, 123.2, 121.7, 108.3, 106.4, 101.6, 79.7, 42.3, 37.8, 23.3 ppm; MS (ESI) *m/z* 283.1 [M+Na]$^+$; HRMS (ESI): Exact mass calcd for $C_{13}H_{14}N_2O_2$ [M+H]$^+$ 263.1031, found 263.1026.

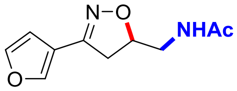

***N*-((3-(Furan-3-yl)-4,5-dihydroisoxazol-5-yl)methyl)acetamide (3h).** Prepared according to the general procedure using corresponding oxime (76 mg, 0.50 mmol). Flash column chromatography (SiO_2, 1–2% MeOH in CH_2Cl_2) yielded a white solid (41 mg, 39%). R_f = 0.20 (5% MeOH in CH_2Cl_2); m.p. : 153~156 ^{o}C; ^{1}H NMR (600 MHz, $CDCl_3$) δ 7.61 (s, 1H), 7.44 (s, 1H), 6.72 (s, 1H), 6.18 (s, 1H), 4.79 (s, 1H), 3.52 (dd, *J* = 57.5, 12.5 Hz, 2H), 3.26 (dd, *J* = 15.7, 10.5 Hz, 1H), 2.96 (dd, *J* = 15.9, 6.1 Hz,

1H), 1.98 (s, 3H) ppm; ^{13}C NMR (100 MHz, $CDCl_3$) δ 170.8, 150.8, 144.3, 142.6, 116.6, 108.0, 79.4, 42.2, 38.1, 23.2 ppm; MS (ESI) *m/z* 229.4 $[M+Na]^+$; HRMS (ESI): Exact mass calcd for $C_{10}H_{12}N_2O_3$ $[M+H]^+$ 209.0921, found 209.0921.

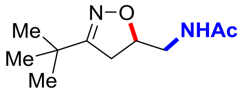

***N*-((3-(*tert*-Butyl)-4,5-dihydroisoxazol-5-yl)methyl)acetamide (3i).** Prepared according to the general procedure using corresponding oxime (71 mg, 0.50 mmol). Flash column chromatography (SiO_2, 1–2% MeOH in CH_2Cl_2) yielded a light yellow solid (49 mg, 50%). R_f = 0.30 (5% MeOH in CH_2Cl_2); m.p. : 79~81 °C; ^{1}H NMR (400 MHz, $CDCl_3$) δ 6.14 (s, 1H), 4.72 – 4.57 (m, 1H), 3.47 – 3.32 (m, 2H), 3.01 (dd, *J* = 17.1, 10.5 Hz, 1H), 2.71 (dd, *J* = 17.1, 6.5 Hz, 1H), 1.97 (s, 3H), 1.16 (s, 9H) ppm; ^{13}C NMR (100 MHz, $CDCl_3$) δ 170.8, 166.6, 78.6, 42.5, 37.0, 33.0, 28.0, 27.7, 23.2 ppm; MS (ESI) *m/z* 219.6 $[M+Na]^+$; HRMS (ESI): Exact mass calcd for $C_{10}H_{18}N_2O_2$ $[M+H]^+$ 199.1437, found 199.1441.

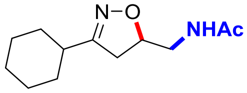

***N*-((3-Cyclohexyl-4,5-dihydroisoxazol-5-yl)methyl)acetamide (3j).** Prepared according to the general procedure using corresponding oxime (163 mg, 0.98 mmol). Flash column chromatography (SiO_2, 1–2% MeOH in CH_2Cl_2) yielded a brown liquid (97 mg, 44%). R_f = 0.30 (5% MeOH in CH_2Cl_2); ^{1}H NMR (400 MHz, $CDCl_3$) δ 6.25 (s, 1H), 4.64 – 4.55 (m, 1H), 3.42 (ddd, *J* = 14.2, 5.9, 3.4 Hz, 1H), 3.34 (dt, *J* = 14.2, 6.1 Hz, 1H), 2.99 – 2.91 (m, 1H), 2.65 (dd, *J* = 17.3, 6.6 Hz, 1H), 2.34 (dt, *J* = 11.1, 5.3 Hz, 1H), 1.96 (s, 3H), 1.83 – 1.71 (m, 4H), 1.69 – 1.62 (m, 1H), 1.33 – 1.17 (m, 5H) ppm; ^{13}C NMR (100 MHz, $CDCl_3$) δ 170.90, 163.43, 78.11, 77.48, 77.16, 76.84, 42.56, 38.12, 37.28, 30.44, 30.40, 25.83, 25.73, 23.16 ppm; MS (ESI) *m/z* 245.2 $[M+Na]^+$; HRMS (ESI): Exact mass calcd for $C_{10}H_{18}N_2O_2$ $[M+H]^+$ 225.1592, found 225.1597.

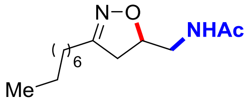

***N*-((3-*n*-Octyl-4,5-dihydroisoxazol-5-yl)methyl)acetamide (3k).** Prepared according to the general procedure using corresponding oxime (99 mg, 0.50 mmol). Flash column chromatography (SiO_2, 0.5–1% MeOH in CH_2Cl_2) yielded a white solid (48 mg, 38%). R_f = 0.30 (5% MeOH in CH_2Cl_2); m.p. : 94~97 °C; ^{1}H NMR (400 MHz, $CDCl_3$) δ 6.08 (s, 1H), 4.65 (dtd, *J* = 10.1, 6.7, 3.2 Hz, 1H), 3.51 (ddd, *J* = 14.1, 5.6, 3.1 Hz, 1H), 3.40 – 3.29 (m, 1H), 2.99 (dd, *J* = 17.2, 10.5 Hz, 1H), 2.65 (dd, *J* = 17.3, 7.0 Hz, 1H), 2.36 – 2.27 (m, 2H), 1.57 – 1.48 (m, 2H), 1.27 (dd, *J* = 7.2, 4.6 Hz, 11H), 0.87 (t, *J* = 6.9 Hz, 3H) ppm; ^{13}C NMR (100 MHz, $CDCl_3$) δ 170.8, 159.8, 78.2, 42.4, 39.8, 31.9, 29.3,

29.2, 29.1, 27.6, 26.4, 23.0, 22.6, 14.1 ppm; MS (ESI) *m/z* 275.2 $[M+Na]^+$; HRMS (ESI): Exact mass calcd for $C_{10}H_{18}N_2O_2$ $[M+H]^+$ 255.2064, found 255.2067.

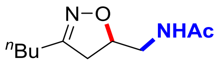

***N*-((3-*n*-Butyl-4,5-dihydroisoxazol-5-yl)methyl)acetamide (3l).** Prepared according to the general procedure using corresponding oxime (153 mg, 1.08 mmol). Flash column chromatography (SiO_2, 1–3% MeOH in CH_2Cl_2) yielded a light yellow solid (62 mg, 31%). R_f = 0.20 (5% MeOH in CH_2Cl_2); m.p. : 79~84 °C; ^{1}H NMR (MHz, $CDCl_3$) δ 6.02 (s, 1H), 4.65 (dtd, *J* = 10.2, 6.7, 3.2 Hz, 1H), 3.51 (ddd, *J* = 14.1, 5.9, 3.2 Hz, 1H), 3.36 (dt, *J* = 14.1, 6.1 Hz, 1H), 2.99 (dd, *J* = 17.2, 10.5 Hz, 1H), 2.66 (dd, *J* = 17.3, 7.0 Hz, 1H), 2.33 (t, *J* = 7.6 Hz, 2H), 2.00 (s, 3H), 1.56 – 1.47 (m, 2H), 1.35 (dq, *J* = 14.4, 7.3 Hz, 2H), 0.92 (t, *J* = 7.3 Hz, 3H) ppm; ^{13}C NMR (100 MHz, $CDCl_3$) δ 170.8, 159.7, 78.3 42.5, 39.8, 28.5, 27.3, 23.2, 22.3, 13.70 ppm; MS (ESI) *m/z* 219.0 $[M+Na]^+$; HRMS (ESI): Exact mass calcd for $C_{10}H_{18}N_2O_2$ $[M+H]^+$ 199.1435, found 199.1441

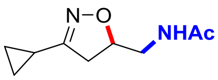

***N*-((3-Cyclopropyl-4,5-dihydroisoxazol-5-yl)methyl)acetamide (3m).** Prepared according to the general procedure using corresponding oxime (90 mg, 0.72 mmol). Flash column chromatography (SiO_2, 1–2% MeOH in CH_2Cl_2) yielded a white solid (26 mg, 20%). R_f = 0.30 (5% MeOH in CH_2Cl_2); m.p. : 109~111 °C; ^{1}H NMR (400 MHz, $CDCl_3$) δ 6.01 (s, 1H), 4.63 (dtd, *J* = 10.2, 6.6, 3.3 Hz, 1H), 3.47 (ddd, *J* = 14.2, 5.7, 3.3 Hz, 1H), 3.41 – 3.32 (m, 1H), 2.84 (dd, *J* = 16.9, 10.4 Hz, 1H), 2.51 (dd, *J* = 17.0, 6.8 Hz, 1H), 2.01 (s, 3H), 1.76 (tt, *J* = 8.4, 5.1 Hz, 1H), 0.93 – 0.87 (m, 2H), 0.77 – 0.71 (m, 2H) ppm; ^{13}C NMR (100 MHz, $CDCl_3$) δ 171.0, 161.8, 78.4, 42.5, 37.9, 23.3, 9.1, 6.3, 6.2 ppm; MS (ESI) *m/z* 203.1 $[M+Na]^+$; HRMS (ESI): Exact mass calcd for $C_{10}H_{18}N_2O_2$ $[M+H]^+$ 183.1128, found 183.1127.

Hypervalent iodine-mediated intra-/intermolecular diamination

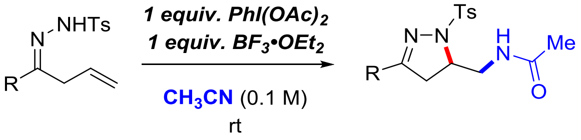

General procedure for pyrazoline formation: To a stirred solution of the corresponding hydrazone (1 equiv) in MeCN (0.1 M) were added $PhI(OAc)_2$ (1 equiv) followed by $BF_3 \cdot OEt_2$ (1 equiv) at room temperature. After 18 h, the reaction mixture was quenched with 1 N aqueous

solution of sodium thiosulfate (1 mL), dried over $MgSO_4$ and concentrated in vacuo. The obtained residue was purified by flash column chromatography (SiO_2, MeOH in CH_2Cl_2) to afford the corresponding pyrazolines.

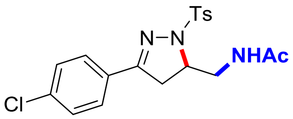

***N*-((3-(4-Chlorophenyl)-1-tosyl-4,5-dihydro-1*H*-pyrazol-5-yl)methyl)acetamide (5a).** Prepared according to the general procedure using the corresponding hydrazone (63 mg, 0.18 mmol). Flash column chromatography (SiO_2, 1–3% MeOH in CH_2Cl_2) yielded a white solid (34 mg, 47%). R_f = 0.30 (5% MeOH in CH_2Cl_2); m.p. : 134~138 ^oC; ^1H NMR (400 MHz, $CDCl_3$) δ 7.67 (d, *J* = 8.3 Hz, 2H), 7.50 – 7.46 (m, 2H), 7.25 – 7.18 (m, 4H), 6.45 (t, *J* = 5.8 Hz, 1H), 3.96 – 3.87 (m, 1H), 3.77 (ddd, *J* = 14.1, 7.2, 4.2 Hz, 1H), 3.64 – 3.56 (m, 1H), 2.97 – 2.88 (m, 2H), 2.30 (s, 3H), 1.94 (s, 3H) ppm; ^{13}C NMR (100 MHz, $CDCl_3$) δ 171.4, 158.0, 144.8, 136.9, 131.2, 129.8, 129.0, 128.6, 128.3, 126.7, 61.7, 42.1, 37.2, 23.3, 21.6 ppm; MS (ESI) *m/z* 428.3 $[M+Na]^+$; HRMS (ESI): Exact mass calcd for $C_{19}H_{20}ClN_3O_3S$ $[M+H]^+$ 406.0995, found 406.0987

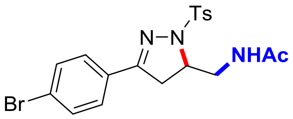

***N*-((3-(4-Bromophenyl)-1-tosyl-4,5-dihydro-1*H*-pyrazol-5-yl)methyl)acetamide (5b).** Prepared according to the general procedure using the corresponding hydrazone (196 mg, 0.50 mmol). Flash column chromatography (SiO_2, 0.5% MeOH in CH_2Cl_2) yielded a white solid (97 mg, 43%). R_f = 0.35 (5% MeOH in CH_2Cl_2); m.p. : 86~90 ^oC; ^1H NMR (600 MHz, $CDCl_3$) δ 7.51 (d, *J* = 7.8 Hz, 2H), 7.06 – 7.00 (m, 2H), 6.28 (s, 1H), 3.75 (t, *J* = 9.7 Hz, 1H), 3.65 – 3.58 (m, 1H), 3.45 (d, *J* = 14.2 Hz, 1H), 2.82 – 2.71 (m, 2H), 2.15 (s, 3H), 1.79 (s, 3H) ppm; ^{13}C NMR (100 MHz, $CDCl_3$) δ 171.3, 158.1, 144.8, 131.9, 131.2, 129.8, 129.3, 128.6, 128.4, 125.4, 61.8, 42.1, 37.1, 23.3, 21.7 ppm; MS (ESI) *m/z* 471.1, 473.2 $[M+Na]^+$; HRMS (ESI): Exact mass calcd for $C_{19}H_{20}BrN_3O_3S$ $[M+H]^+$ 450.0488, found 450.0482.

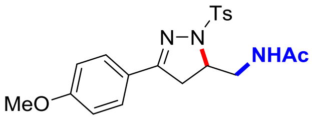

***N*-((3-(4-Methoxyphenyl)-1-tosyl-4,5-dihydro-1*H*-pyrazol-5-yl)methyl)acetamide (5c).** Prepared according to the general procedure using the corresponding hydrazone (158 mg, 0.46 mmol). Flash column chromatography (SiO_2, 1–2% MeOH in CH_2Cl_2) yielded a light yellow solid (45 mg, 24%). R_f = 0.35 (5% MeOH in CH_2Cl_2); m.p. : 155~160 ^oC; ^1H NMR (600 MHz, $CDCl_3$) δ 7.75 (d, *J* = 8.3 Hz, 2H), 7.59 (d, *J* = 8.9 Hz, 2H), 7.27 (d, *J* = 8.3 Hz, 2H), 6.91 – 6.86 (m, 2H),

6.29 (s, 1H), 3.93 (d, *J* = 4.4 Hz, 1H), 3.79 (dd, *J* = 6.9, 4.7 Hz, 1H), 3.71 – 3.64 (m, 1H), 2.96 (qd, *J* = 17.5, 9.7 Hz, 2H), 2.38 (s, 3H), 2.02 (s, 3H) ppm; ^{13}C NMR (100 MHz, $CDCl_3$) δ 171.1, 161.8, 144.6, 131.3, 129.7, 128.7, 128.6, 126.9, 122.9, 114.1, 61.3, 55.4, 42.1, 37.3, 23.4, 21.6 ppm; MS (ESI) *m/z* 422.2 $[M+Na]^+$; HRMS (ESI): Exact mass calcd for $C_{20}H_{23}N_3O_4S$ $[M+H]^+$ 402.1484, found 402.1482.

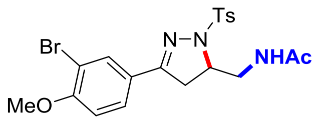

***N*-((3-(3-Bromo-4-methoxyphenyl)-1-tosyl-4,5-dihydro-1*H*-pyrazol-5-yl)methyl)acetamide (5d).** Prepared according to the general procedure using the corresponding hydrazone (211 mg, 0.50 mmol). Flash column chromatography (SiO_2, 1% MeOH in CH_2Cl_2) yielded a white solid (66 mg, 28%). R_f = 0.40 (5% MeOH in CH_2Cl_2); m.p. : 101~103 °C; ^{1}H NMR (600 MHz, $CDCl_3$) δ 7.79 (s, 1H), 7.68 (d, *J* = 7.7 Hz, 2H), 7.47 (d, *J* = 8.1 Hz, 1H), 7.23 – 7.18 (m, 2H), 6.79 (d, *J* = 7.9 Hz, 1H), 6.39 (s, 1H), 3.89 (s, 1H), 3.84 (s, 3H), 3.76 (d, *J* = 12.6 Hz, 1H), 3.60 (d, *J* = 13.8 Hz, 1H), 2.97 – 2.77 (m, 2H), 2.32 (s, 3H), 1.96 (s, 3H) ppm; ^{13}C NMR (100 MHz, $CDCl_3$) δ 171.3, 157.8, 144.8, 131.9, 131.2, 129.8, 128.6, 128.5, 127.7, 124.2, 112.1, 111.5, 61.5, 56.4, 42.1, 37.2, 23.3, 21.7 ppm; MS (ESI) *m/z* 490.4, 492.2 $[M+Na]^+$; HRMS (ESI): Exact mass calcd for $C_{20}H_{22}BrN_3O_4S$ $[M+H]^+$ 480.0592, found 480.0587.

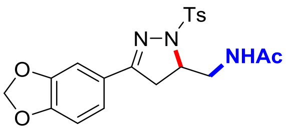

***N*-((3-(Benzo[*d*][1,3]dioxol-5-yl)-1-tosyl-4,5-dihydro-1*H*-pyrazol-5-yl)methyl)acetamide (5e).** Prepared according to the general procedure using the corresponding hydrazone (143 mg, 0.40 mmol). Flash column chromatography (SiO_2, 1–3% MeOH in CH_2Cl_2) yielded a light yellow solid (44 mg, 26%). R_f = 0.25 (5% MeOH in CH_2Cl_2); m.p. : 177~180 °C; ^{1}H NMR (600 MHz, $CDCl_3$) δ 7.72 (d, *J* = 7.7 Hz, 2H), 7.26 – 7.22 (m, 3H), 6.94 (d, *J* = 8.0 Hz, 1H), 6.73 (d, *J* = 7.8 Hz, 1H), 6.48 (s, 1H), 5.97 (s, 2H), 3.92 (s, 1H), 3.81 – 3.74 (m, 1H), 3.65 (d, *J* = 14.0 Hz, 1H), 2.92 (ddd, *J* = 26.0, 17.4, 9.9 Hz, 2H), 2.36 (s, 3H), 2.01 (s, 3H) ppm; ^{13}C NMR (100 MHz, $CDCl_3$) δ 171.1, 158.6, 150.1, 148.2, 144.6, 131.4, 129.7, 128.6, 128.5, 124.6, 122.3, 108.1, 106.5, 101.6, 61.4, 42.2, 37.4, 23.3, 21.6 ppm; MS (ESI) *m/z* 436.5 $[M+Na]^+$; HRMS (ESI): Exact mass calcd for $C_{20}H_{21}N_3O_5S$ $[M+H]^+$ 416.1278, found 416.1275.

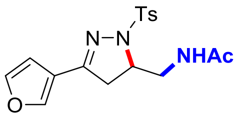

***N*-((3-(Furan-3-yl)-1-tosyl-4,5-dihydro-1*H*-pyrazol-5-yl)methyl)acetamide (5f).** Prepared according to the general procedure

using the corresponding hydrazone (170 mg, 0.50 mmol). Flash column chromatography (SiO_2, 1–2% MeOH in CH_2Cl_2) yielded a white solid (28 mg, 16%). R_f = 0.40 (5% MeOH in CH_2Cl_2); m.p. : 83~86 °C; ^{1}H NMR (600 MHz, $CDCl_3$) δ 7.73 (d, *J* = 7.8 Hz, 2H), 7.56 (s, 1H), 7.42 (s, 1H), 7.28 (d, *J* = 7.8 Hz, 2H), 6.77 (s, 1H), 6.42 (s, 1H), 3.92 (s, 1H), 3.77 (s, 1H), 3.65 (d, *J* = 13.1 Hz, 1H), 2.85 (dd, *J* = 22.2, 14.8 Hz, 2H), 2.39 (s, 3H), 2.03 (s, 3H) ppm; ^{13}C NMR (100 MHz, $CDCl_3$) δ 171.2, 153.2, 144.7, 144.3, 143.7, 131.3, 129.7, 128.6, 118.6, 108.2, 61.0, 42.1, 37.8, 23.3, 21.6 ppm; MS (ESI) *m/z* 382.7 $[M+Na]^+$; HRMS (ESI): Exact mass calcd for $C_{17}H_{19}N_3O_4S$ $[M+H]^+$ 362.1172, found 362.1169.

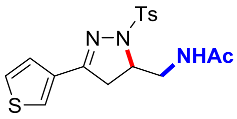

***N*-((3-(Thiophen-3-yl)-1-tosyl-4,5-dihydro-1*H*-pyrazol-5-yl)methyl)acetamide (5g).** Prepared according to the general procedure using the corresponding hydrazone (150 mg, 0.46 mmol). Flash column chromatography (SiO_2, 1–2% MeOH in CH_2Cl_2) yielded a gray solid (33 mg, 20%). R_f = 0.30 (5% MeOH in CH_2Cl_2); m.p. : 172~177 °C; ^{1}H NMR (600 MHz, $CDCl_3$) δ 7.75 (d, *J* = 7.8 Hz, 2H), 7.42 (d, *J* = 4.9 Hz, 1H), 7.28 (d, *J* = 7.8 Hz, 2H), 7.13 (s, 1H), 7.01 (d, *J* = 3.2 Hz, 1H), 6.50 (s, 1H), 3.96 (s, 1H), 3.83 (d, *J* = 13.2 Hz, 1H), 3.68 (d, *J* = 13.6 Hz, 1H), 3.06 – 2.94 (m, 2H), 2.39 (s, 3H), 2.05 (s, 3H) ppm; ^{13}C NMR (150 MHz, $CDCl_3$) δ 171.62, 154.74, 144.88, 133.92, 131.36, 129.88, 129.85, 128.84, 127.70, 61.69, 42.32, 38.11, 23.33, 21.76 ppm; MS (ESI) *m/z* 398.8 $[M+Na]^+$; HRMS (ESI): Exact mass calcd for $C_{17}H_{19}N_3O_3S_2$ $[M+H]^+$ 378.0947, found 378.0941

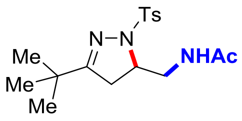

***N*-((3-(*tert*-Butyl)-1-tosyl-4,5-dihydro-1*H*-pyrazol-5-yl)methyl)acetamide (5h).** Prepared according to the general procedure using the corresponding hydrazone (70 mg, 0.24 mmol). Flash column chromatography (SiO_2, 1% MeOH in CH_2Cl_2) yielded a colorless caramel (36 mg, 43%). R_f = 0.30 (5% MeOH in CH_2Cl_2); ^{1}H NMR (600 MHz, $CDCl_3$) δ 7.68 (d, *J* = 7.8 Hz, 2H), 7.29 (d, *J* = 7.8 Hz, 2H), 6.31 (s, 1H), 3.86 – 3.77 (m, 1H), 3.64 (dd, *J* = 13.7, 6.5 Hz, 1H), 3.51 (d, *J* = 14.0 Hz, 1H), 2.52 (d, *J* = 9.2 Hz, 2H), 2.40 (s, 3H), 1.99 (s, 3H), 1.02 (s, 9H) ppm; ^{13}C NMR (150 MHz, $CDCl_3$) δ 172.3, 171.1, 144.6, 131.4, 129.5, 128.8, 60.9, 42.8, 36.5, 34.4, 27.9, 23.3, 21.7 ppm; MS (ESI) *m/z* 372.4 $[M+Na]^+$; HRMS (ESI): Exact mass calcd for $C_{20}H_{21}N_3O_5S$ $[M+H]^+$ 352.1690, found 352.1689.

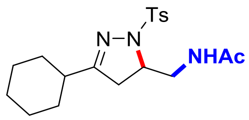

***N*-((3-Cyclohexyl-1-tosyl-4,5-dihydro-1*H*-pyrazol-5-yl)methyl)acetamide (5i).** Prepared according to the general procedure using the corresponding hydrazone (160 mg, 0.50 mmol). Flash column chromatography (SiO_2, 0.5–2% MeOH in CH_2Cl_2) yielded a pale yellow caramel (35 mg, 18%). R_f = 0.30 (5% MeOH in CH_2Cl_2); ^{1}H NMR (400 MHz, $CDCl_3$) δ 7.68 (d, *J* = 8.3 Hz, 2H), 7.28 (d, *J* = 8.0 Hz, 2H), 6.23 (s, 1H), 3.81 – 3.72 (m, 1H), 3.60 (ddd, *J* = 13.9, 6.9, 4.9 Hz, 1H), 3.55 – 3.45 (m, 1H), 2.48 (d, *J* = 9.3 Hz, 2H), 2.40 (s, 3H), 2.28 – 2.16 (m, 1H), 1.99 (s, 3H), 1.70 – 1.59 (m, 5H), 1.23 – 1.11 (m, 5H) ppm; ^{13}C NMR (100 MHz, $CDCl_3$) δ 171.1, 168.9, 144.5, 131.4, 129.5, 128.7, 60.1, 42.7, 39.2, 37.8, 30.2, 30.0, 25.7, 25.6, 25.5, 23.3, 21.6 ppm; MS (ESI) *m/z* 398.3 $[M+Na]^+$; HRMS (ESI): Exact mass calcd for $C_{20}H_{21}N_3O_5S$ $[M+H]^+$ 378.1851, found 378.1846

Crystal structure report for 3a

Table S2: Data collection details for **3a.**

Axis	dx/mm	2θ/°	ω/°	φ/°	χ/°	Width/°	Frames	Time/s	Wavelength/Å	Voltage/kV	Current/mA	Temperature/K
Phi	33.952	104.11	105.68	0.00	-23.00	0.70	514	20.00	1.54184	50	50.0	n/a
Omega	33.952	104.11	-5.12	80.00	61.50	0.70	158	20.00	1.54184	50	50.0	n/a
Omega	33.952	104.11	-5.12	-120.00	61.50	0.70	158	20.00	1.54184	50	50.0	n/a
Omega	33.952	-2.13	-100.90	144.00	44.50	0.70	136	20.00	1.54184	50	50.0	n/a
Omega	33.952	-2.13	-100.90	-144.00	44.50	0.70	136	20.00	1.54184	50	50.0	n/a
Omega	33.952	104.11	-5.12	0.00	61.50	0.70	158	20.00	1.54184	50	50.0	n/a
Phi	33.952	104.11	15.08	0.00	23.00	0.70	514	20.00	1.54184	50	50.0	n/a
Omega	33.952	104.11	-5.12	160.00	61.50	0.70	158	20.00	1.54184	50	50.0	n/a
Omega	33.952	-2.13	-100.90	-72.00	44.50	0.70	136	20.00	1.54184	50	50.0	n/a
Phi	33.952	104.11	107.35	0.00	-44.00	0.70	514	20.00	1.54184	50	50.0	n/a
Omega	33.952	104.11	-5.12	-40.00	61.50	0.70	158	20.00	1.54184	50	50.0	n/a
Omega	33.952	104.11	-5.12	-160.00	61.50	0.70	158	20.00	1.54184	50	50.0	n/a
Omega	33.952	104.11	107.50	-120.00	-44.50	0.70	136	20.00	1.54184	50	50.0	n/a
Omega	33.952	104.11	107.50	120.00	-44.50	0.70	136	20.00	1.54184	50	50.0	n/a
Omega	33.952	104.11	-5.12	40.00	61.50	0.70	158	20.00	1.54184	50	50.0	n/a

Table S3: Sample and crystal data for **3a.**

**Identification code**	20171218_HKB	
**Chemical formula**	$C_{48}H_{48}Cl_4N_8O_8$	
**Formula weight**	1006.74 g/mol	
**Temperature**	293(2) K	
**Wavelength**	1.54184 Å	
**Crystal size**	0.095 x 0.111 x 0.125 mm	
**Crystal system**	orthorhombic	
**Space group**	P c a 21	
**Unit cell dimensions**	a = 8.711(2) Å	α = 90.00(3)°
	b = 4.877(2) Å	β = 90.00(3)°
	c = 29.462(6) Å	γ = 90.00(3)°
**Volume**	1251.7(6) $Å^3$	
**Z**	1	
**Density (calculated)**	1.336 g/cm^3	
**Absorption coefficient**	2.646 mm^{-1}	
**F(000)**	524	

Table S4: Data collection and structure refinement for **3a.**

**Theta range for data collection**	9.09 to 76.04°
**Index ranges**	-10<=h<=10, -6<=k<=6, -35<=l<=36
**Reflections collected**	21763
**Independent reflections**	2457 [R(int) = 0.1599]
**Coverage of independent reflections**	95.1%
**Max. and min. transmission**	0.7780 and 0.7540
**Structure solution technique**	direct methods
**Structure solution program**	SHELXS-97 (Sheldrick 2008)
**Refinement method**	Full-matrix least-squares on F^2
**Refinement program**	SHELXL-2014 (Sheldrick 2014)
**Function minimized**	Σ w(F_o^2 - F_c^2)^2
**Data / restraints / parameters**	2457 / 1 / 155
**Goodness-of-fit on F^2**	1.065
**Final R indices**	2405 data; I>2σ(I) R1 = 0.0703, wR2 = 0.1203
	all data R1 = 0.0717, wR2 = 0.1209
**Weighting scheme**	$w=1/[\sigma^2(F_o^2)+(0.0247P)^2+0.4272P]$ where $P=(F_o^2+2F_c^2)/3$
**Absolute structure parameter**	0.021(15)
**Largest diff. peak and hole**	0.130 and -0.206 eÅ^{-3}
**R.M.S. deviation from mean**	0.024 eÅ^{-3}

Table S5: Atomic coordinates and equivalent isotropic atomic displacement parameters (Å^2) for **3a.**

U(eq) is defined as one third of the trace of the orthogonalized U_{ij} tensor.

	**x/a**	**y/b**	**z/c**	**U(eq)**
Cl1	0.4072(2)	0.2735(4)	0.29353(5)	0.1236(7)
O1	0.6575(4)	0.5798(5)	0.66449(14)	0.0907(10)
N1	0.6158(4)	0.1441(6)	0.64329(11)	0.0619(8)
C1	0.7813(6)	0.2260(9)	0.70662(18)	0.0831(13)
C7	0.5344(4)	0.2414(7)	0.44105(14)	0.0567(8)
N2	0.6822(4)	0.0718(8)	0.50225(13)	0.0806(11)
O2	0.6996(4)	0.0983(7)	0.54931(10)	0.0876(10)
C2	0.6803(5)	0.3317(6)	0.67033(14)	0.0616(9)
C4	0.6008(4)	0.3168(8)	0.56581(15)	0.0619(9)
C3	0.5159(4)	0.2171(8)	0.60612(16)	0.0669(10)
C5	0.4993(4)	0.3887(8)	0.52544(15)	0.0648(9)
C9	0.3895(6)	0.4376(11)	0.38002(18)	0.0844(13)
C8	0.4282(5)	0.4284(9)	0.42497(15)	0.0714(10)
C6	0.5754(4)	0.2306(7)	0.48871(14)	0.0568(8)
C10	0.4570(6)	0.2614(10)	0.35035(16)	0.0777(12)
C11	0.5612(7)	0.0734(11)	0.36482(18)	0.0883(14)
C12	0.5993(6)	0.0624(10)	0.40963(17)	0.0798(12)

Table S6: Bond lengths (Å) for **3a.**

Cl1-C10	1.730(5)	O1-C2	1.238(4)
N1-C2	1.337(5)	N1-C3	1.444(6)
C1-C2	1.478(7)	C1-H00F	0.96
C1-H00G	0.96	C1-H00H	0.96
C7-C8	1.383(6)	C7-C12	1.393(6)
C7-C6	1.450(6)	N2-C6	1.274(5)
N2-O2	1.401(5)	O2-C4	1.453(5)
C4-C3	1.481(6)	C4-C5	1.523(6)
C4-H4	0.98	C3-H00C	0.97
C3-H00D	0.97	C5-C6	1.485(5)
C5-H00A	0.97	C5-H00B	0.97
C9-C10	1.359(7)	C9-C8	1.367(7)
C9-H00I	0.93	C8-H00E	0.93
C10-C11	1.359(7)	C11-C12	1.362(7)
C11-H00K	0.93	C12-H00J	0.93

Table S7: Bond angles (°) for **3a.**

C2-N1-C3	122.5(3)	C2-C1-H00F	109.5
C2-C1-H00G	109.5	H00F-C1-H00G	109.5
C2-C1-H00H	109.5	H00F-C1-H00H	109.5
H00G-C1-H00H	109.5	C8-C7-C12	117.2(4)
C8-C7-C6	121.4(4)	C12-C7-C6	121.4(4)
C6-N2-O2	109.4(3)	N2-O2-C4	109.6(3)
O1-C2-N1	121.2(4)	O1-C2-C1	122.5(4)
N1-C2-C1	116.3(3)	O2-C4-C3	108.9(3)
O2-C4-C5	104.6(3)	C3-C4-C5	114.3(3)
O2-C4-H4	109.7	C3-C4-H4	109.7
C5-C4-H4	109.7	N1-C3-C4	112.8(3)
N1-C3-H00C	109.0	C4-C3-H00C	109.0
N1-C3-H00D	109.0	C4-C3-H00D	109.0
H00C-C3-H00D	107.8	C6-C5-C4	100.9(3)
C6-C5-H00A	111.6	C4-C5-H00A	111.6
C6-C5-H00B	111.6	C4-C5-H00B	111.6
H00A-C5-H00B	109.4	C10-C9-C8	119.7(5)
C10-C9-H00I	120.2	C8-C9-H00I	120.2
C9-C8-C7	121.2(4)	C9-C8-H00E	119.4
C7-C8-H00E	119.4	N2-C6-C7	120.3(4)
N2-C6-C5	114.4(4)	C7-C6-C5	125.2(3)
C11-C10-C9	120.9(5)	C11-C10-Cl1	119.6(4)
C9-C10-Cl1	119.5(4)	C10-C11-C12	119.6(5)
C10-C11-H00K	120.2	C12-C11-H00K	120.2
C11-C12-C7	121.4(5)	C11-C12-H00J	119.3
C7-C12-H00J	119.3		

Table S8: Anisotropic atomic displacement parameters (Å^2) for **3a.**

The anisotropic atomic displacement factor exponent takes the form: $-2\pi^2[h^2 a^{*2} U_{11} + ... + 2 h k a^* b^* U_{12}]$

	**U_{11}**	**U_{22}**	**U_{33}**	**U_{23}**	**U_{13}**	**U_{12}**
Cl1	0.1339(12)	0.1716(16)	0.0652(8)	0.0055(9)	-0.0082(8)	-0.0135(12)
O1	0.122(3)	0.0402(13)	0.110(3)	0.0013(14)	-0.003(2)	0.0020(14)
N1	0.0769(19)	0.0466(14)	0.0621(19)	0.0055(13)	0.0061(15)	-0.0011(14)
C1	0.104(3)	0.077(3)	0.068(3)	0.003(2)	-0.003(3)	0.001(2)
C7	0.0522(17)	0.0494(17)	0.068(2)	0.0033(14)	0.0061(16)	-0.0078(14)
N2	0.077(2)	0.096(3)	0.069(2)	0.0077(18)	0.0074(16)	0.0342(19)
O2	0.0799(19)	0.118(3)	0.0645(19)	0.0067(17)	0.0013(15)	0.0439(18)
C2	0.074(2)	0.0453(16)	0.066(2)	0.0055(16)	0.0128(17)	0.0008(15)
C4	0.0562(18)	0.0601(18)	0.069(2)	0.0033(16)	0.0013(16)	0.0000(15)
C3	0.061(2)	0.066(2)	0.074(3)	0.0023(18)	0.0063(19)	-0.0064(17)
C5	0.0644(19)	0.0616(19)	0.068(2)	-0.0011(18)	-0.0034(19)	0.0096(16)
C9	0.087(3)	0.088(3)	0.078(3)	0.012(2)	-0.007(2)	0.009(2)
C8	0.072(2)	0.075(3)	0.067(3)	0.0033(19)	0.0022(19)	0.0088(19)
C6	0.0491(16)	0.0526(17)	0.069(2)	0.0056(15)	0.0079(16)	-0.0020(13)
C10	0.076(2)	0.089(3)	0.068(3)	0.005(2)	0.004(2)	-0.020(2)
C11	0.095(3)	0.094(4)	0.076(3)	-0.018(3)	0.009(2)	-0.004(3)
C12	0.085(3)	0.077(3)	0.077(3)	-0.006(2)	0.003(2)	0.012(2)

Table S9: Hydrogen atomic coordinates and isotropic atomic displacement parameters (Å^2) for **3a.**

	**x/a**	**y/b**	**z/c**	**U(eq)**
H00F	0.7831	0.0293	0.7055	0.125
H00G	0.7431	0.2844	0.7356	0.125
H00H	0.8834	0.2955	0.7023	0.125
H4	0.6634	0.4760	0.5742	0.074
H00C	0.4455	0.3589	0.6161	0.08
H00D	0.4554	0.0582	0.5976	0.08
H00A	0.3943	0.3288	0.5301	0.078
H00B	0.5004	0.5840	0.5192	0.078
H00I	0.3173	0.5637	0.3698	0.101
H00E	0.3823	0.5502	0.4451	0.086
H00K	0.6062	-0.0469	0.3443	0.106
H00J	0.6702	-0.0674	0.4194	0.096

Figure S1. ^{1}H NMR (DMSO-d_6) of **3a**

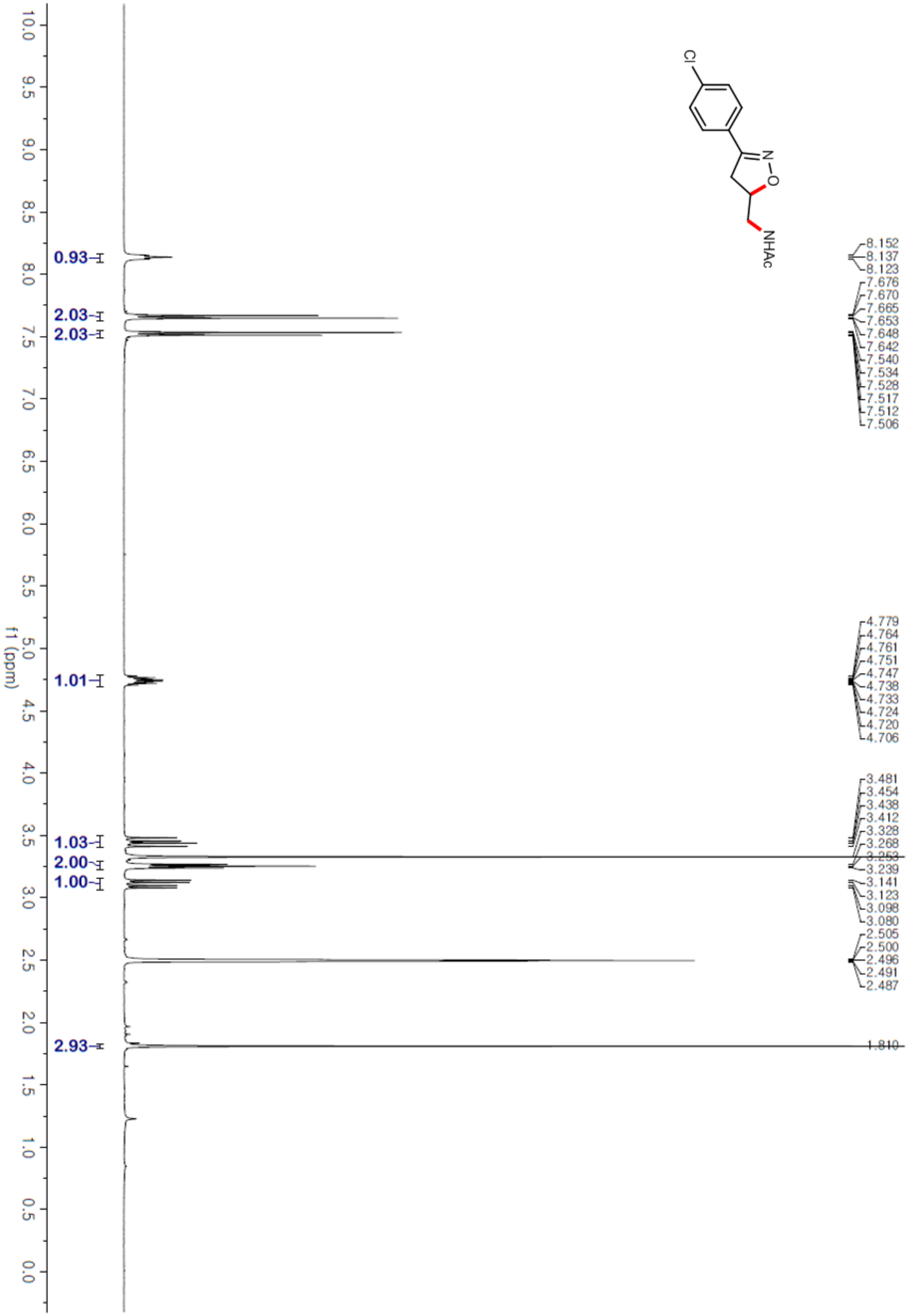

Figure S1. ^{13}C NMR ($CDCl_3$) of **3a**

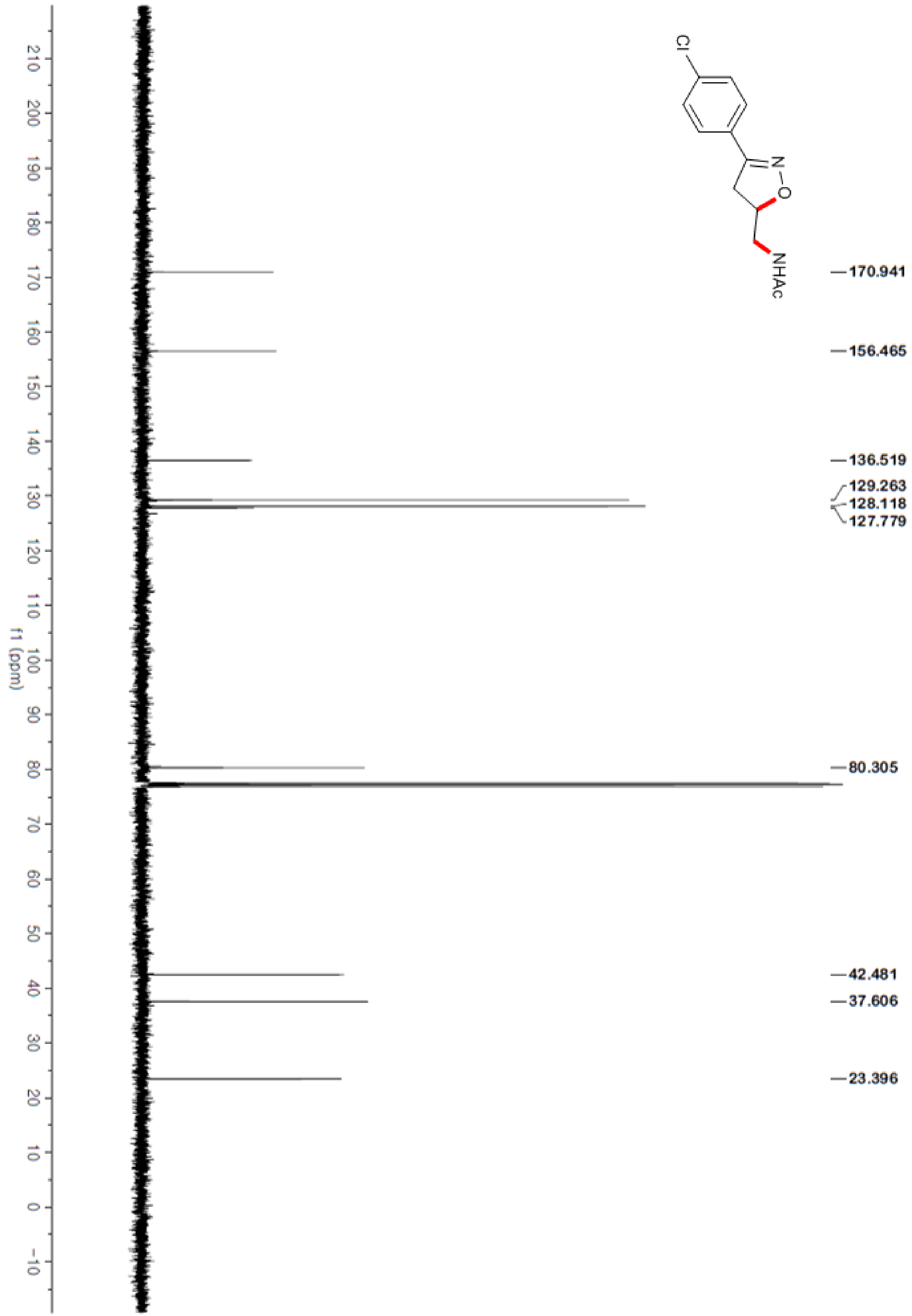

Figure S3. ^{1}H NMR (DMSO-d_6) of **3b**

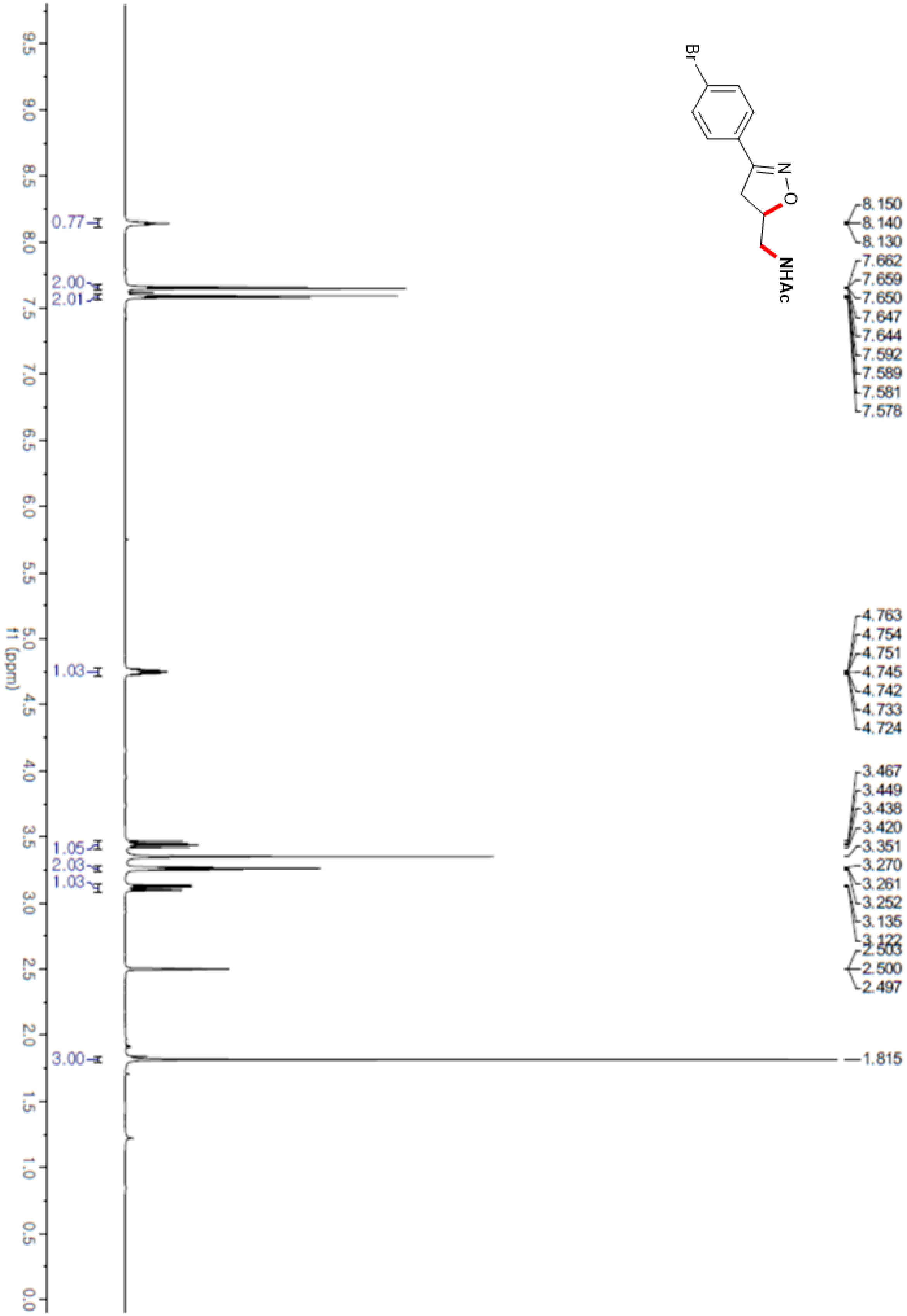

Figure S4. ^{13}C NMR (DMSO-d_6) of **3b**

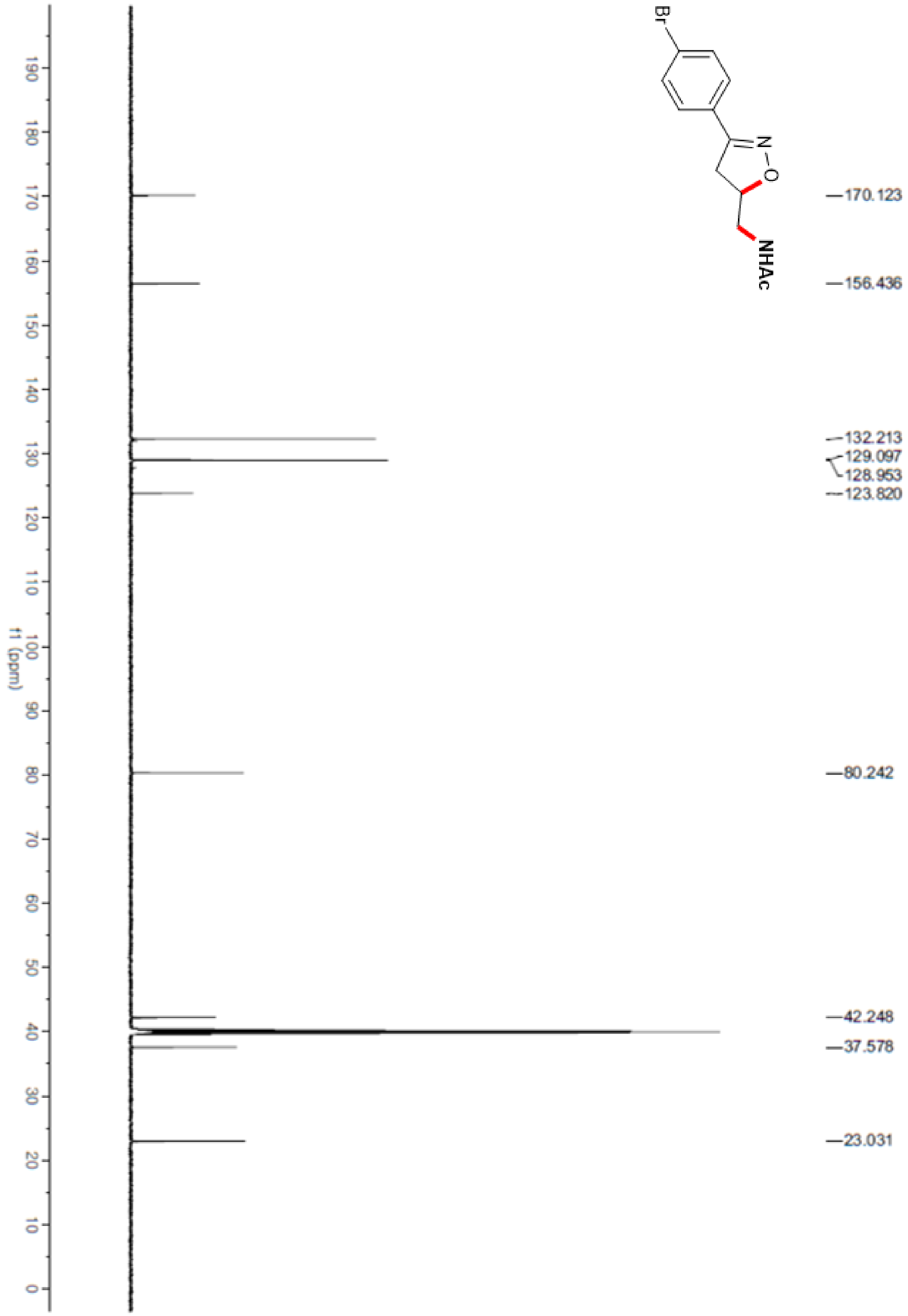

Figure S5. ^{1}H NMR (DMSO-d_6) of **3c**

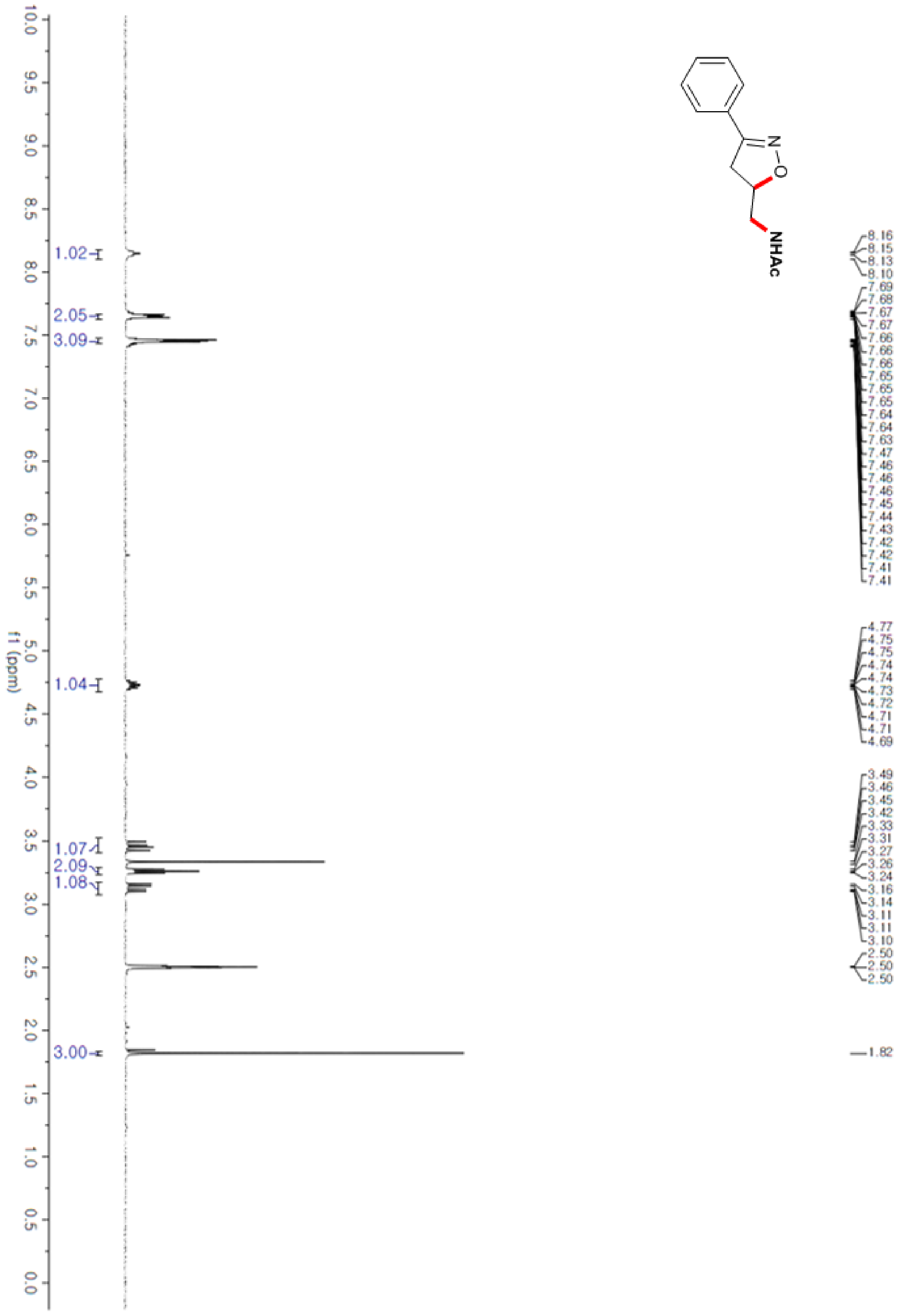

Figure S6. ^{13}C NMR ($CDCl_3$) of **3c**

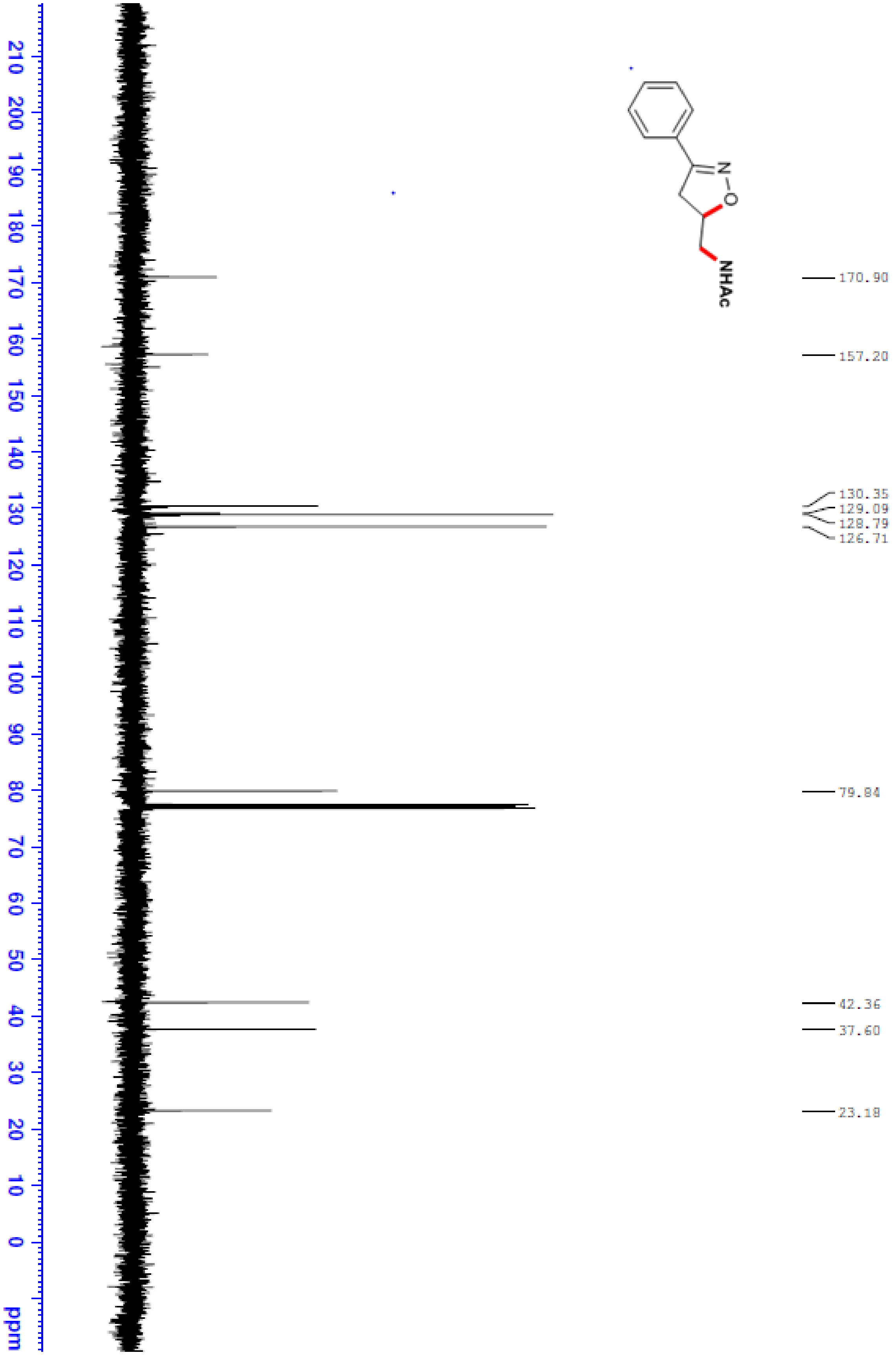

Figure S7. ^{1}H NMR ($CDCl_3$) of **3d**

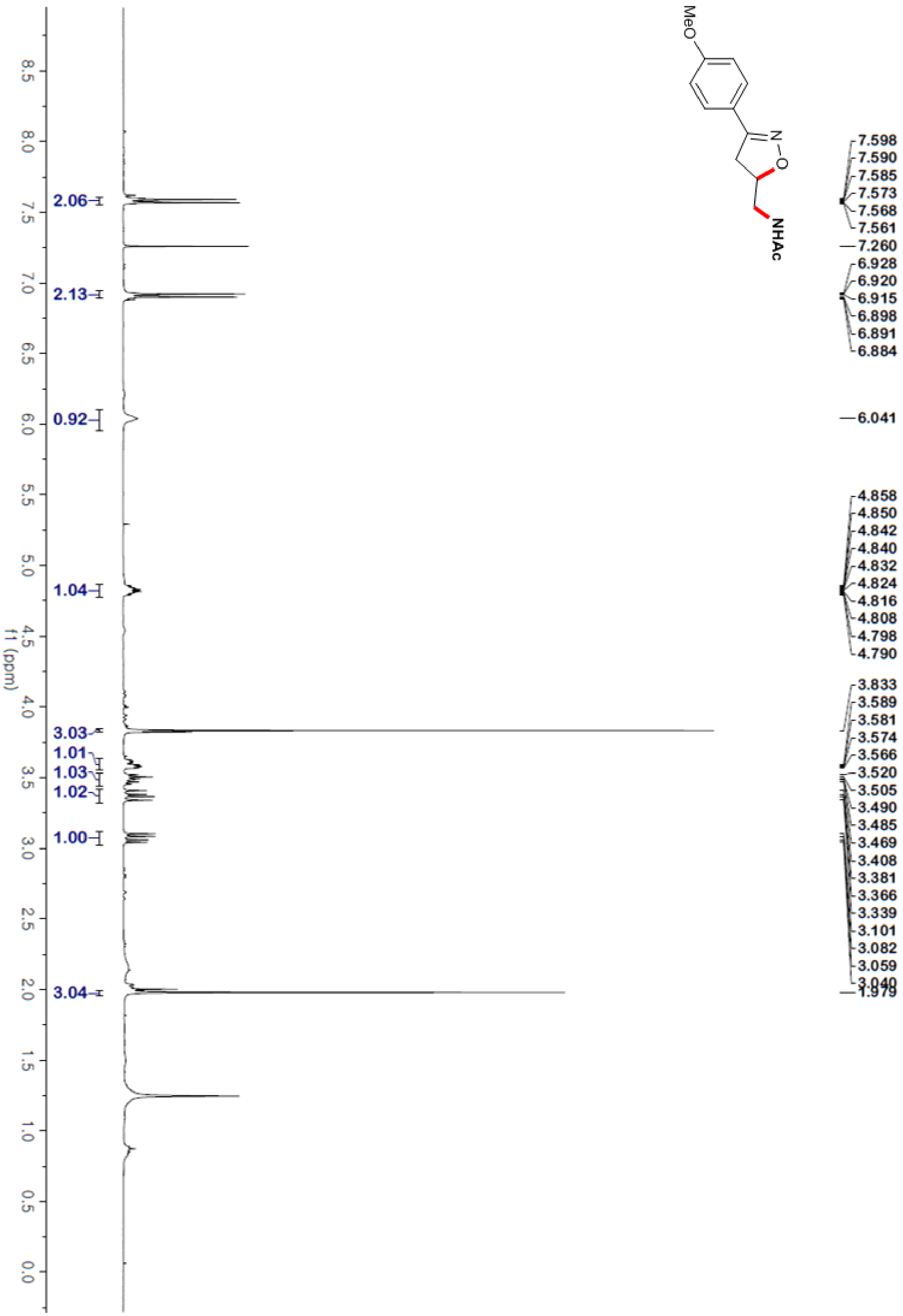

Figure S8. ^{13}C NMR ($CDCl_3$) of **3d**

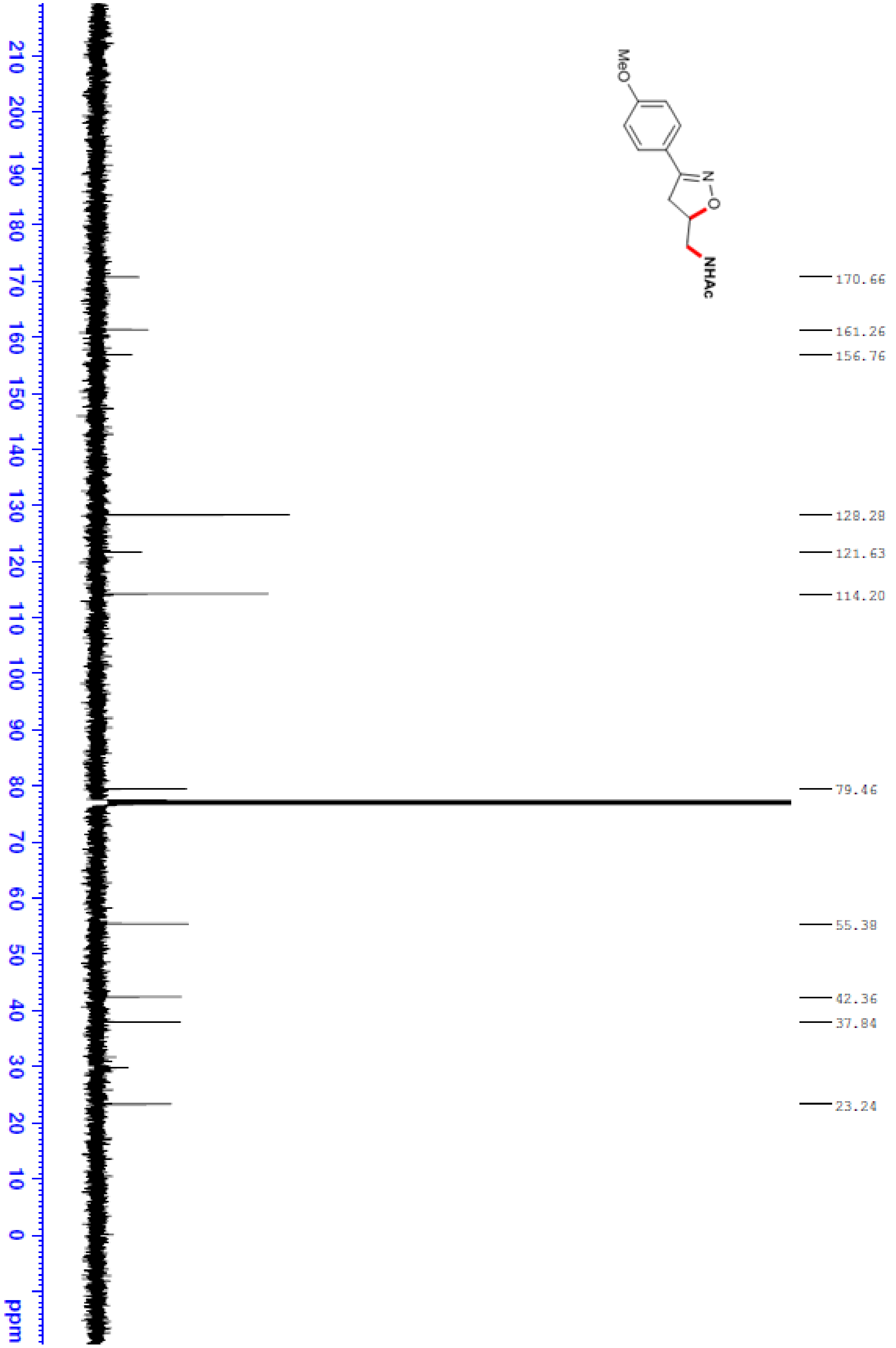

Figure S9. ^{1}H NMR ($CDCl_3$) of **3e**

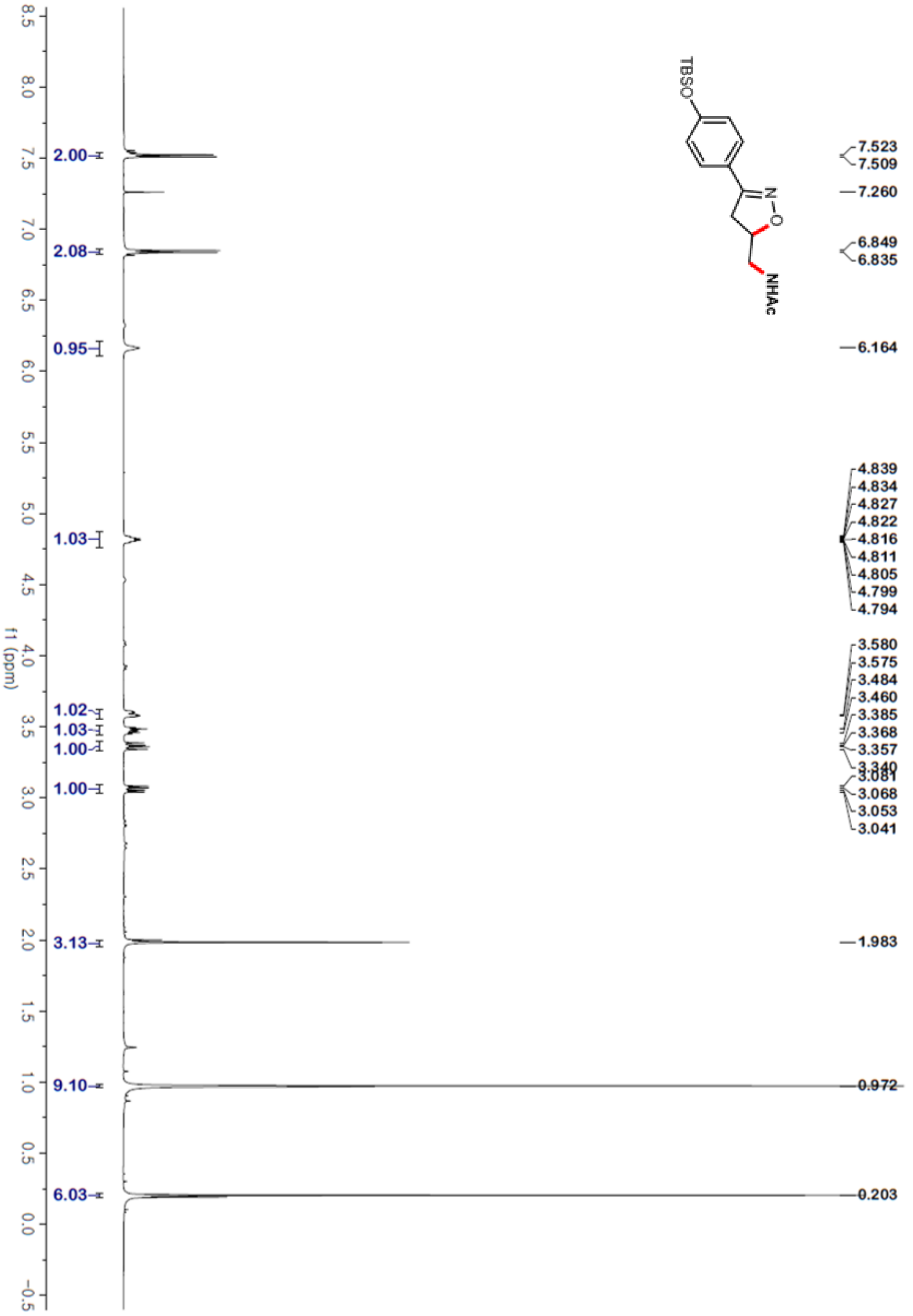

Figure S10. ^{13}C NMR ($CDCl_3$) of **3e**

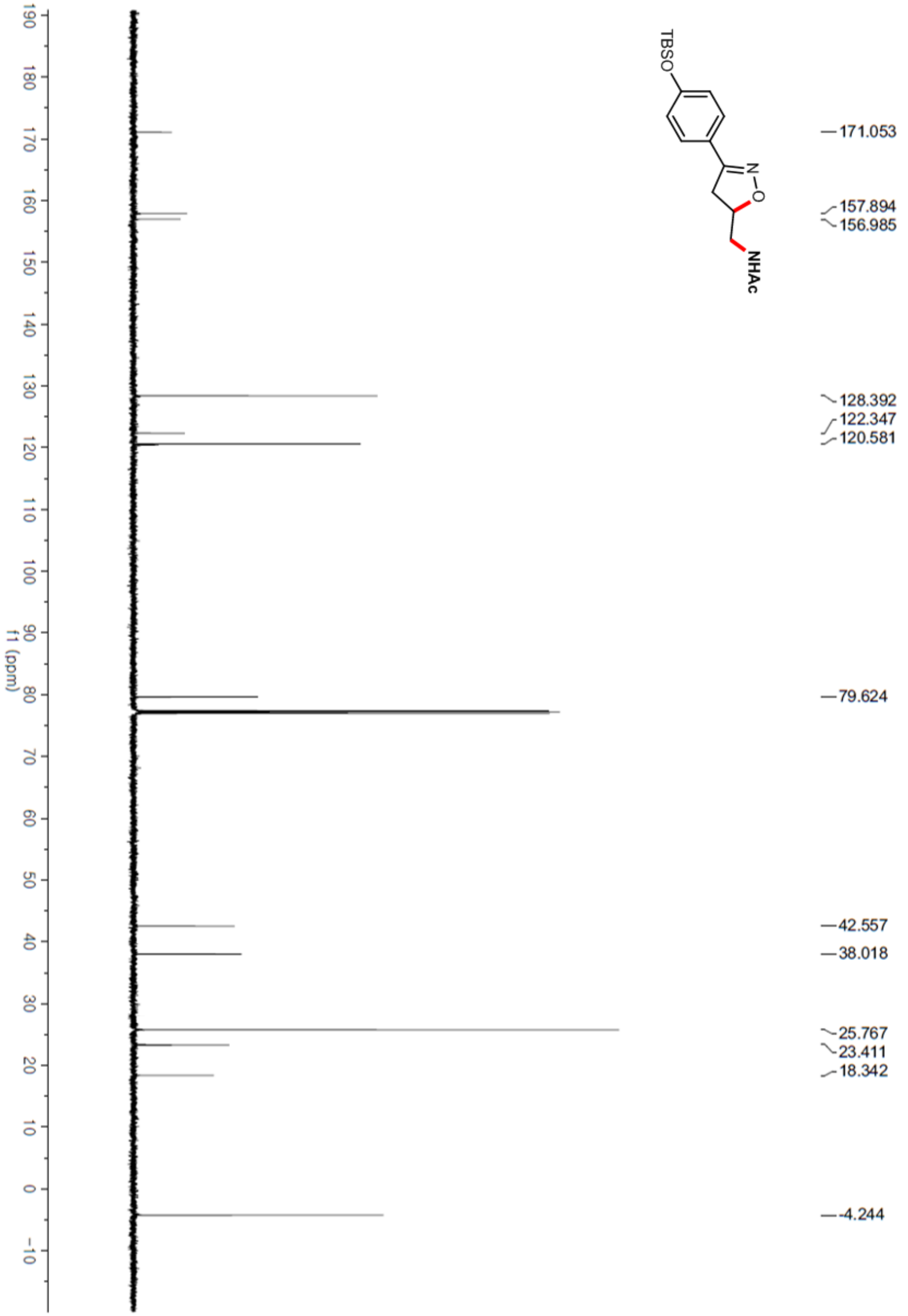

Figure S11. ^{1}H NMR ($CDCl_3$) of **3f**

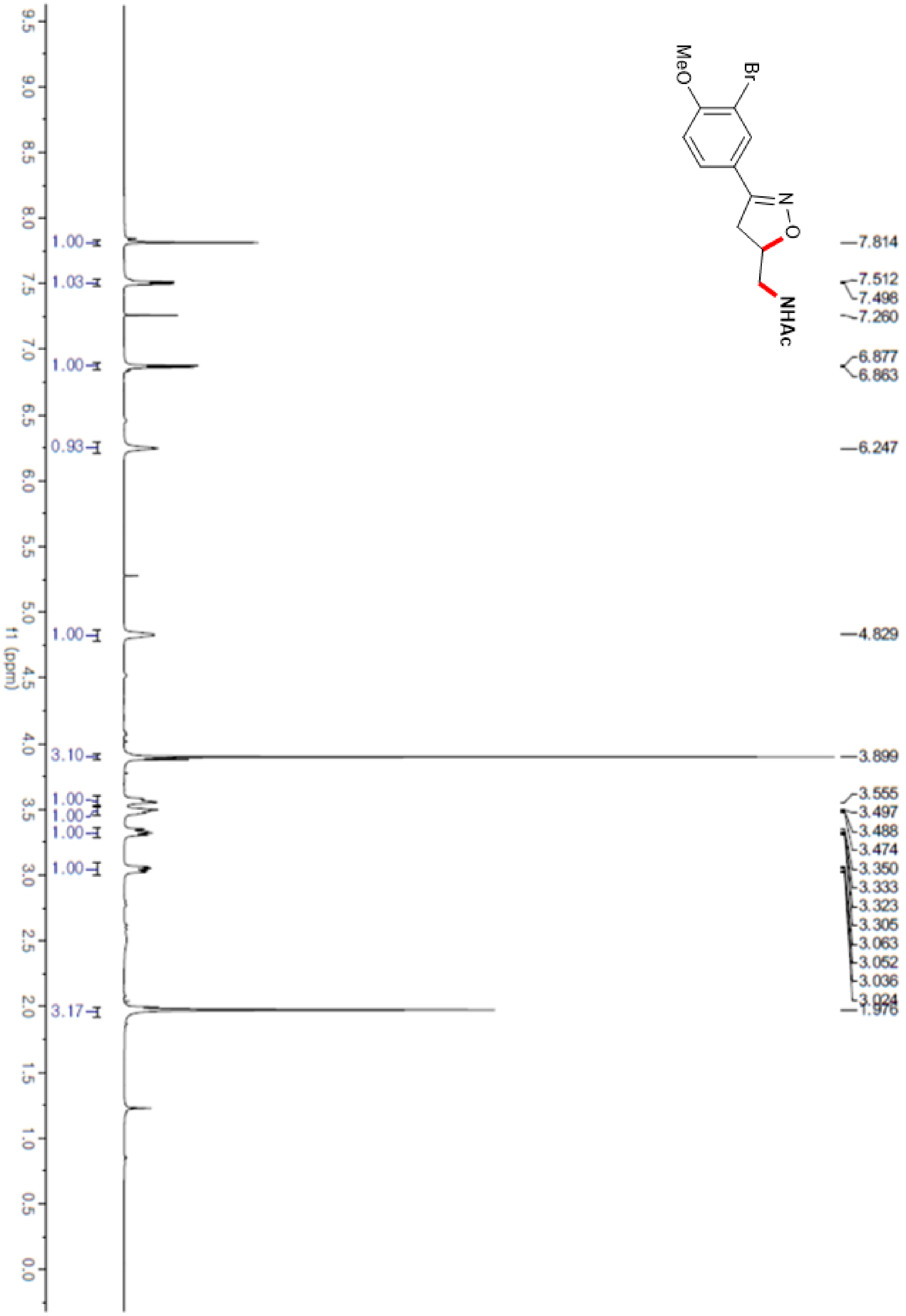

Figure S12. ^{13}C NMR ($CDCl_3$) of **3f**

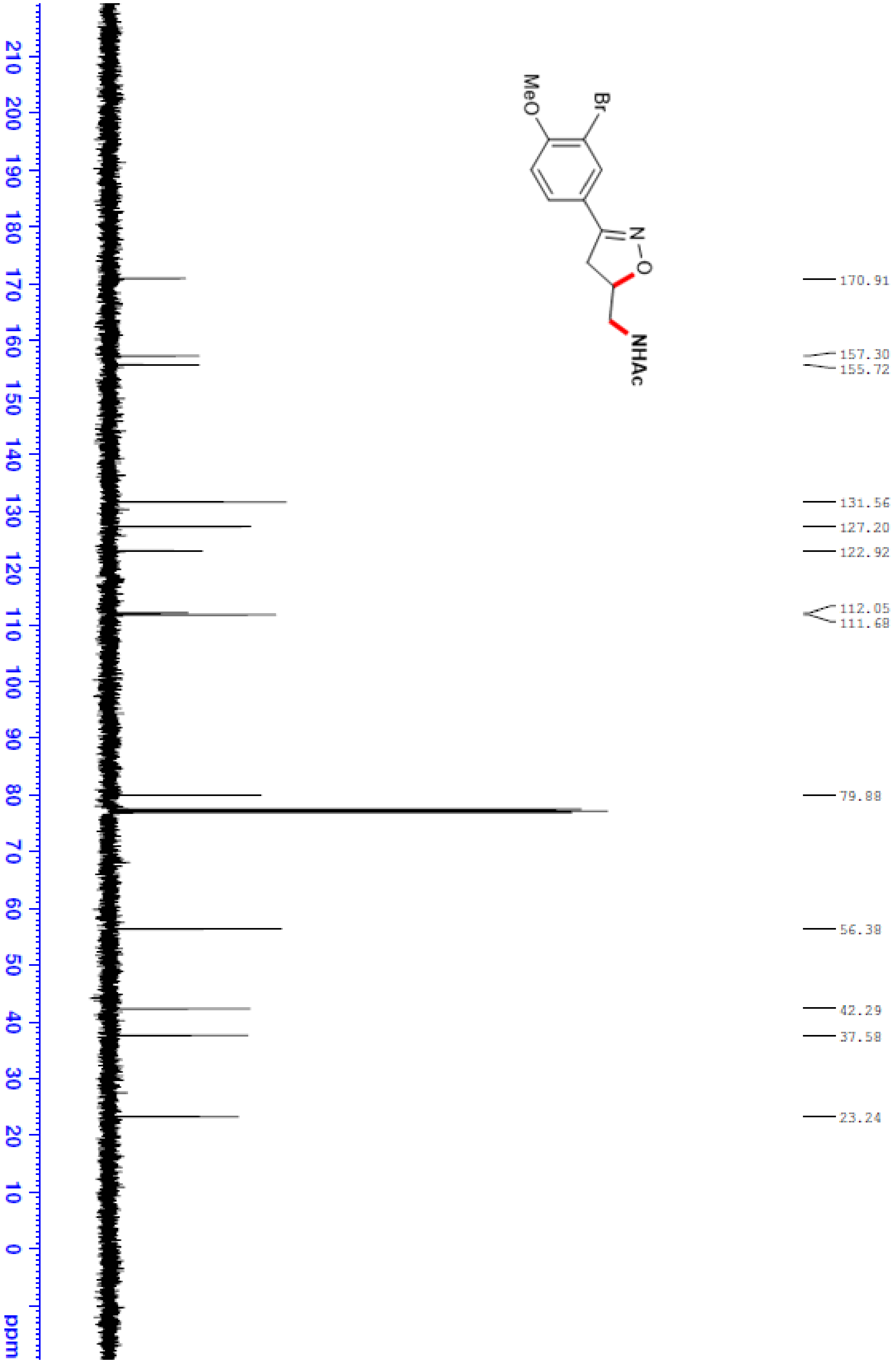

Figure S13. ^{1}H NMR ($CDCl_3$) of **3g**

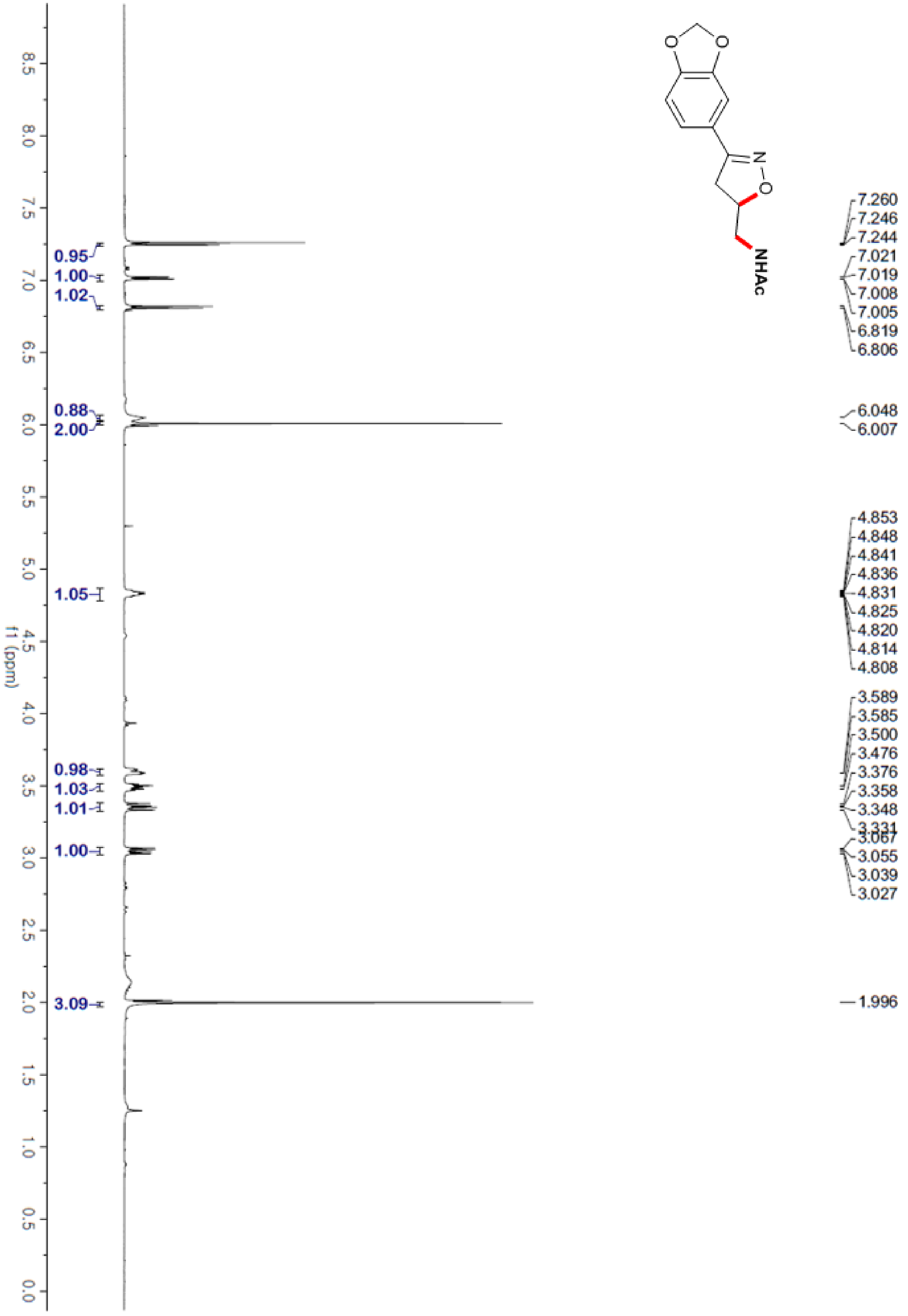

Figure S14. ^{13}C NMR ($CDCl_3$) of **3g**

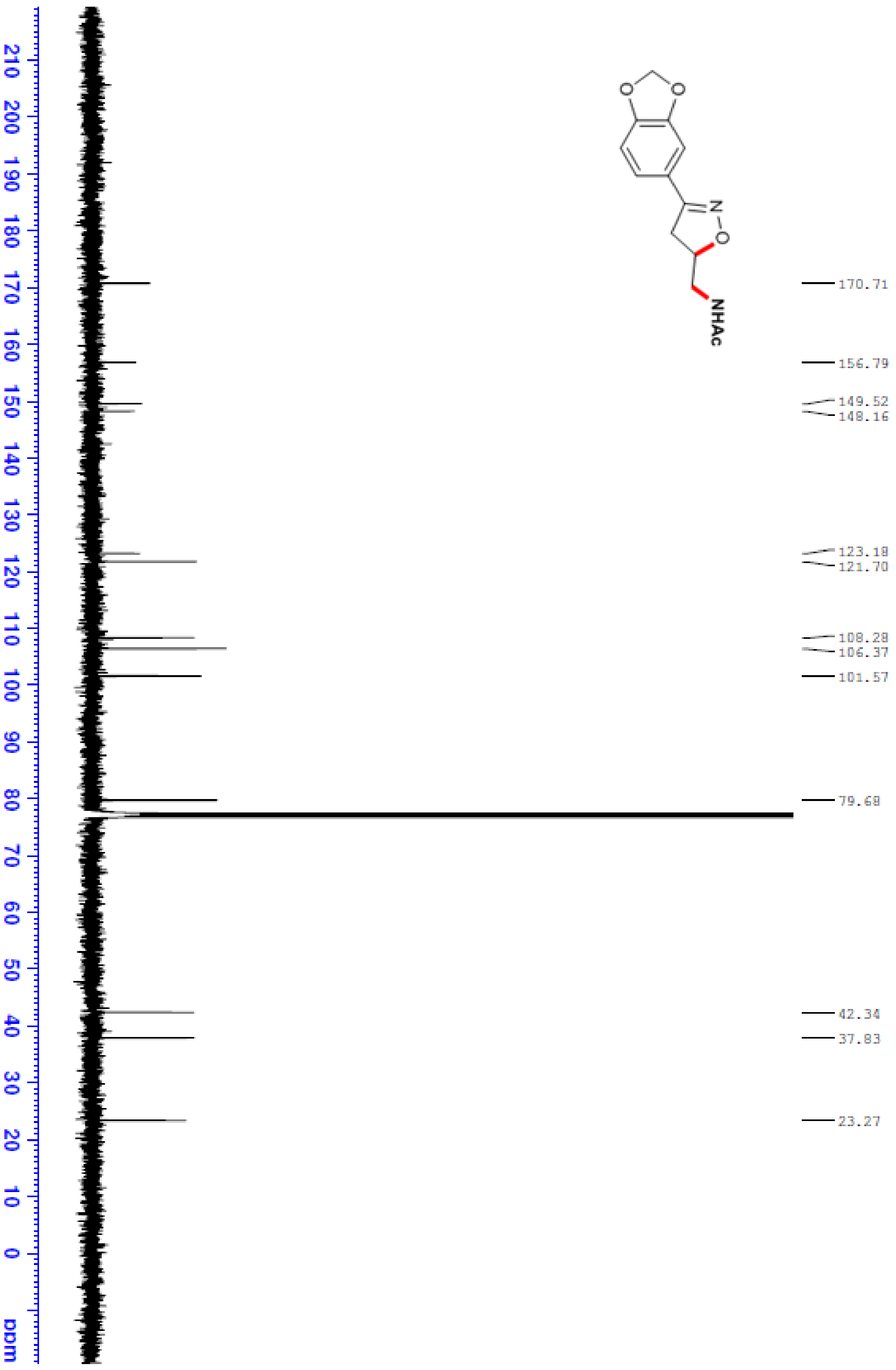

Figure S15. ^{1}H NMR ($CDCl_3$) of **3h**

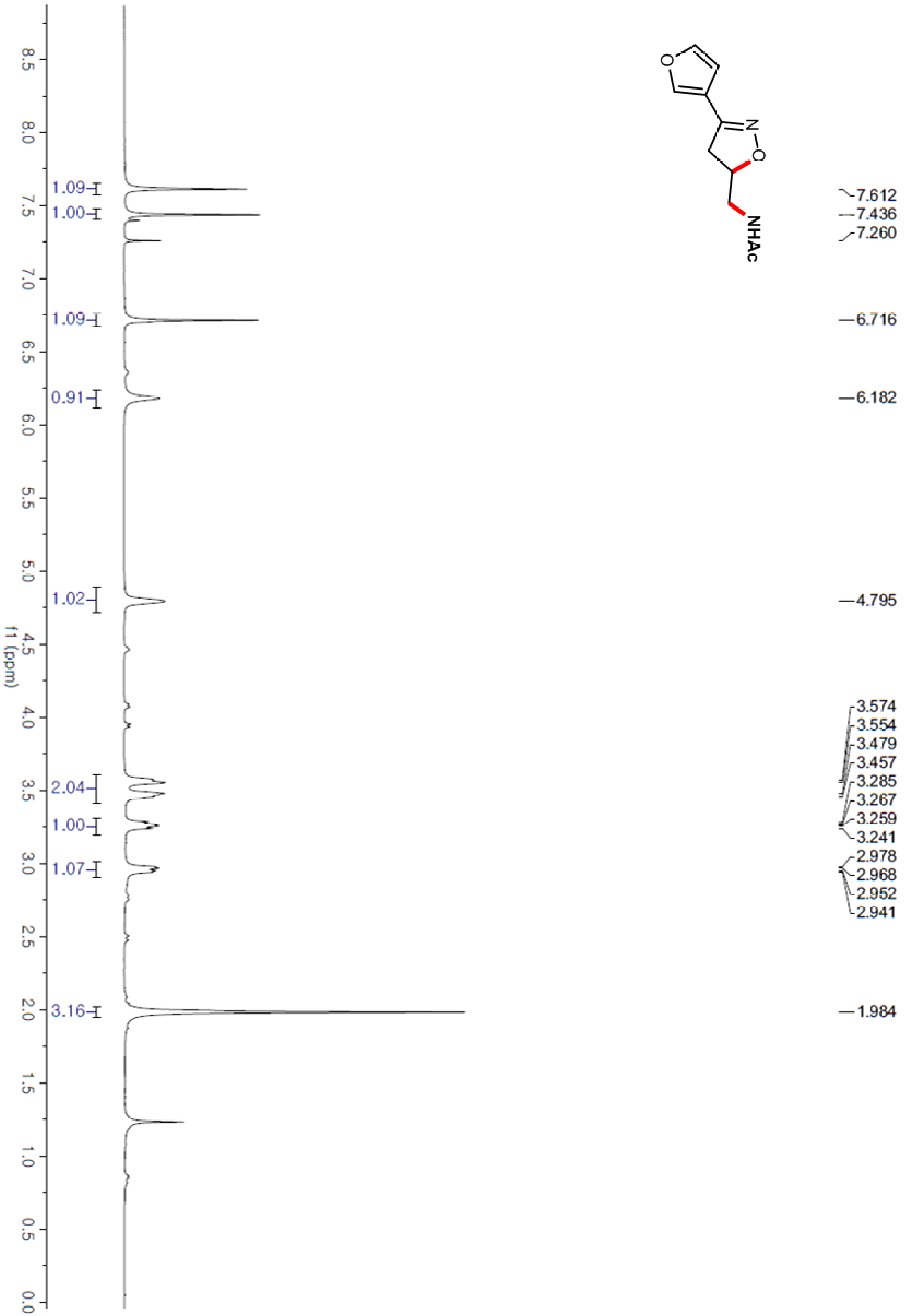

Figure S16. ^{13}C NMR ($CDCl_3$) of **3h**

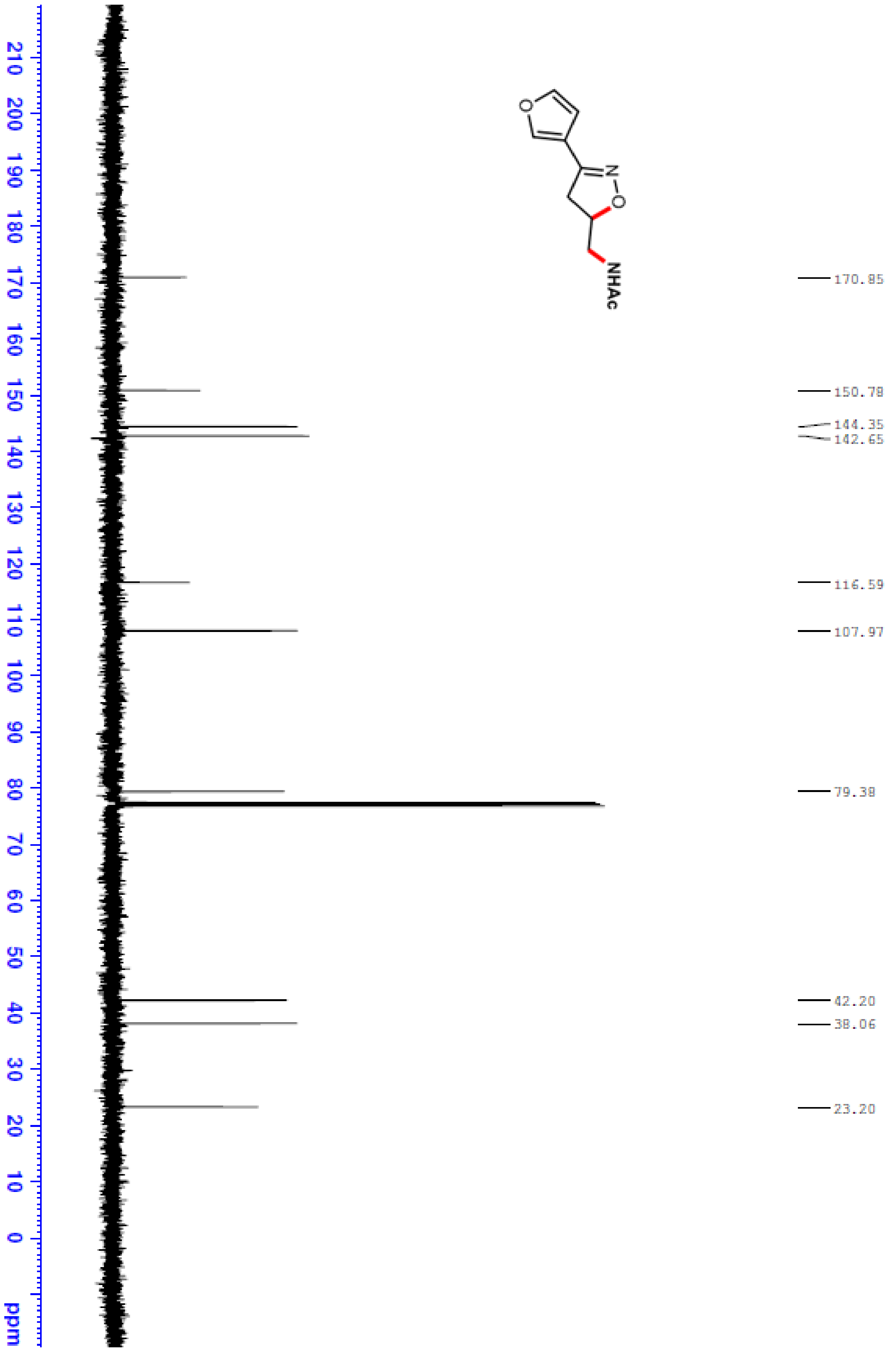

Figure S17. ^{1}H NMR ($CDCl_3$) of **3i**

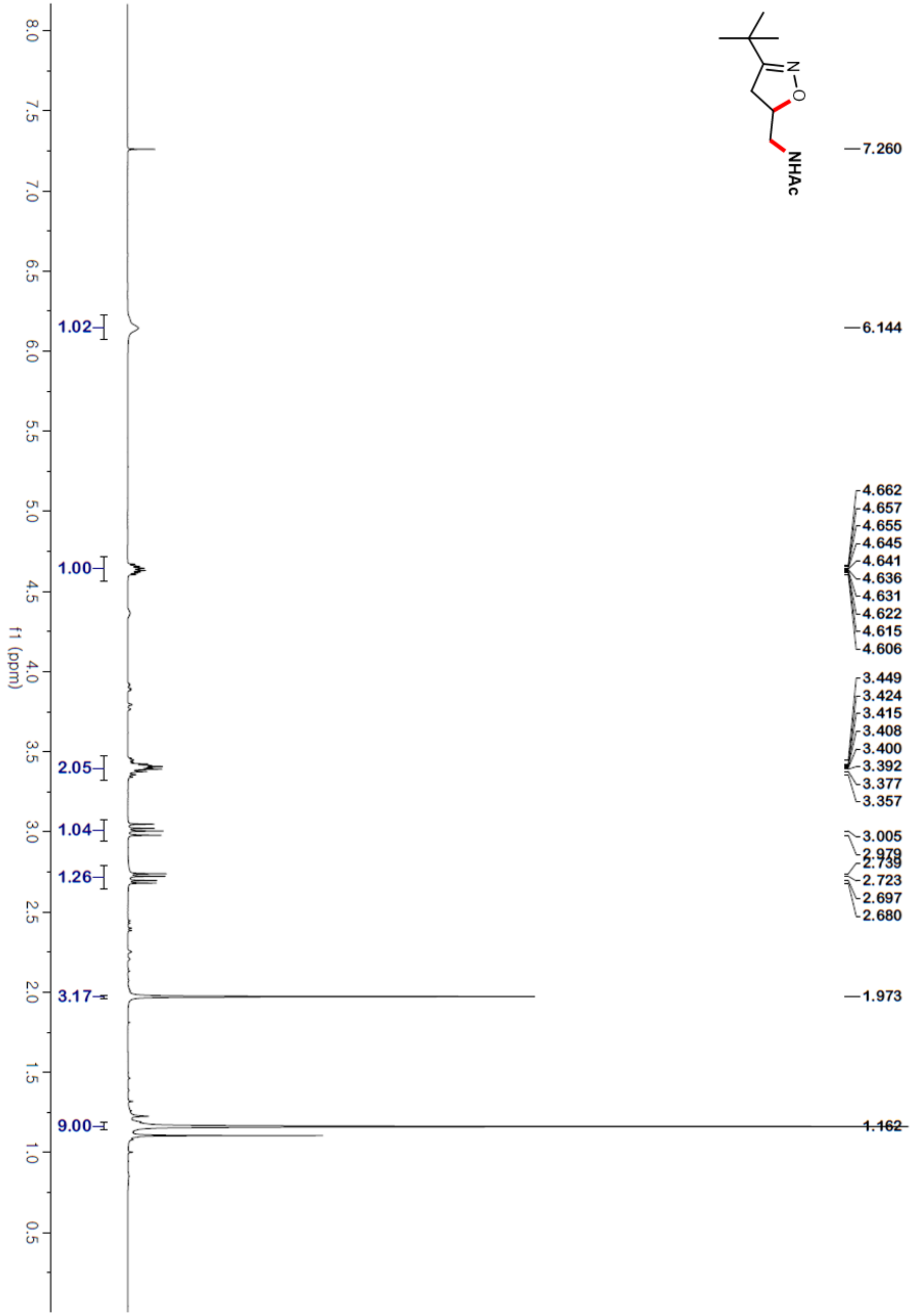

Figure S18. ^{13}C NMR ($CDCl_3$) of **3i**

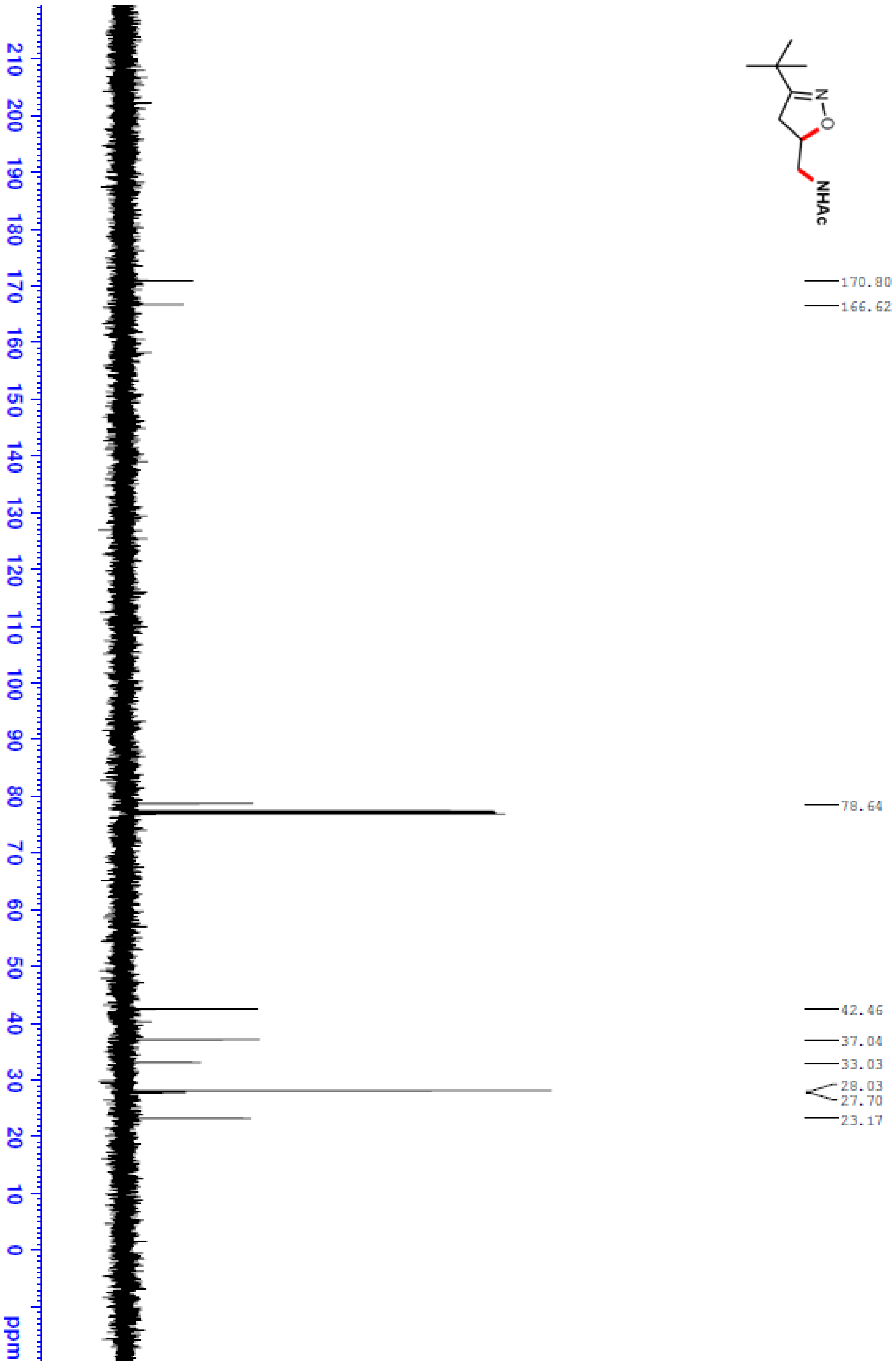

Figure S19. ^{1}H NMR ($CDCl_3$) of **3j**

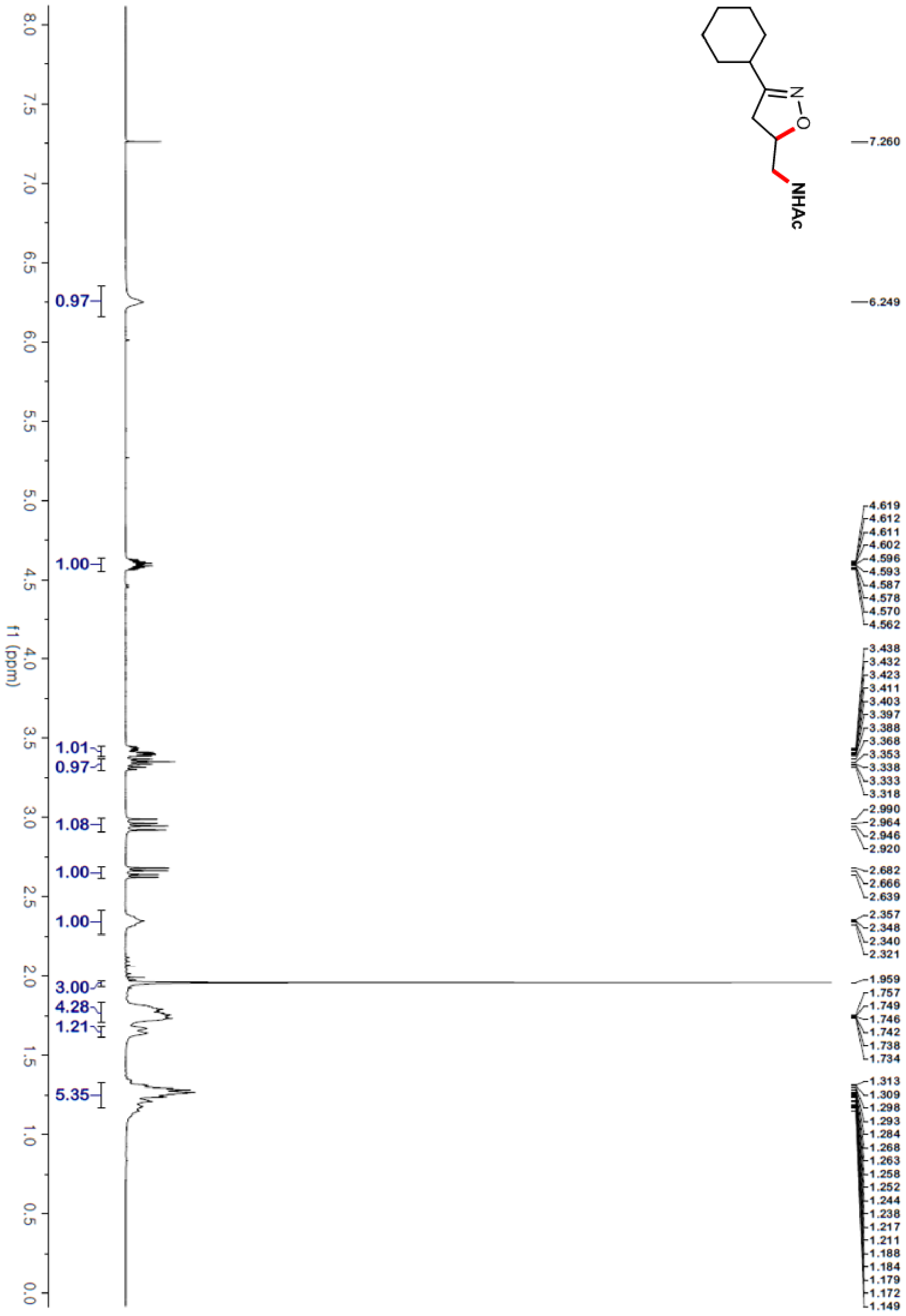

Figure S20. ^{13}C NMR ($CDCl_3$) of **3j**

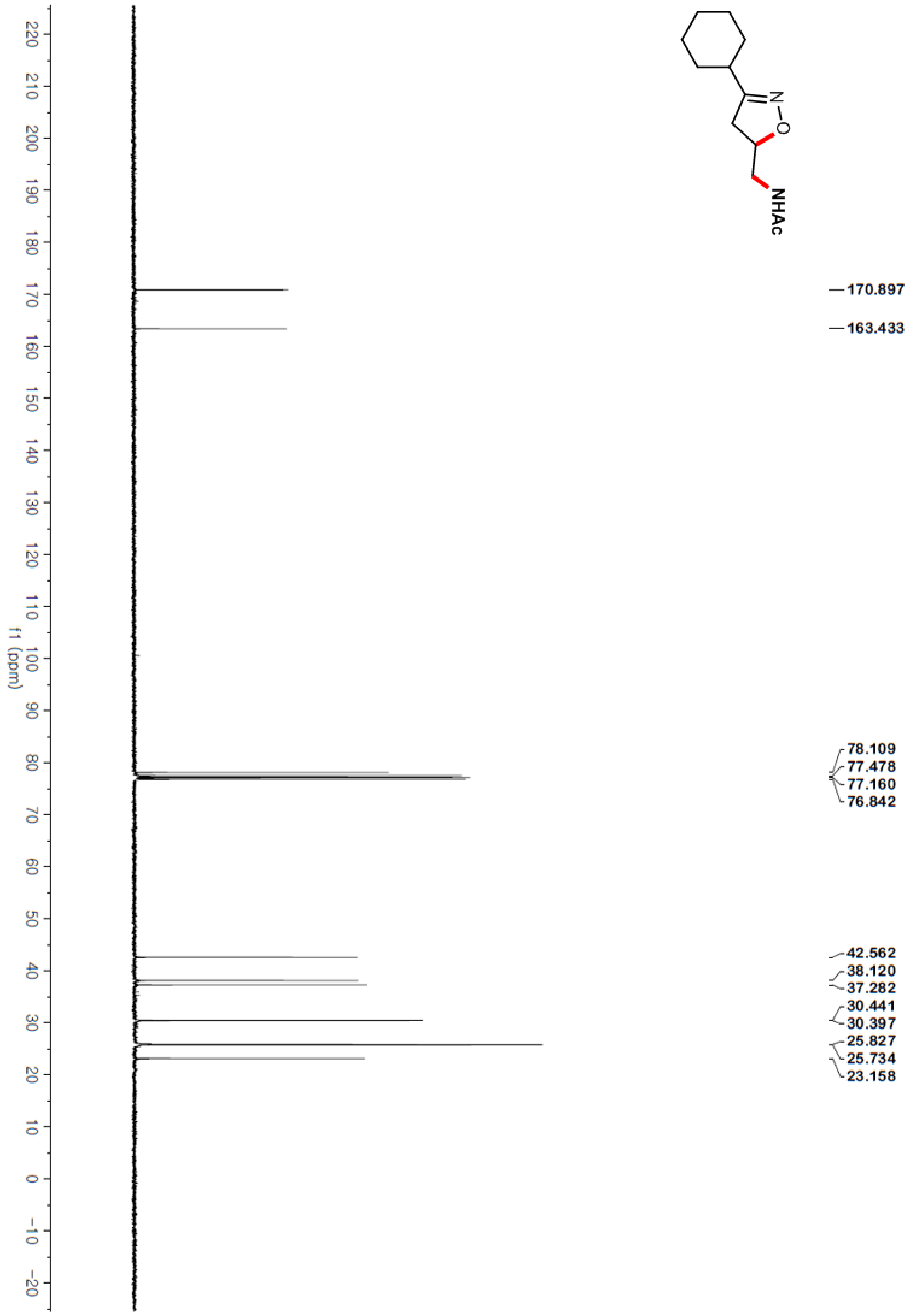

Figure S21. ^{1}H NMR ($CDCl_3$) of **3k**

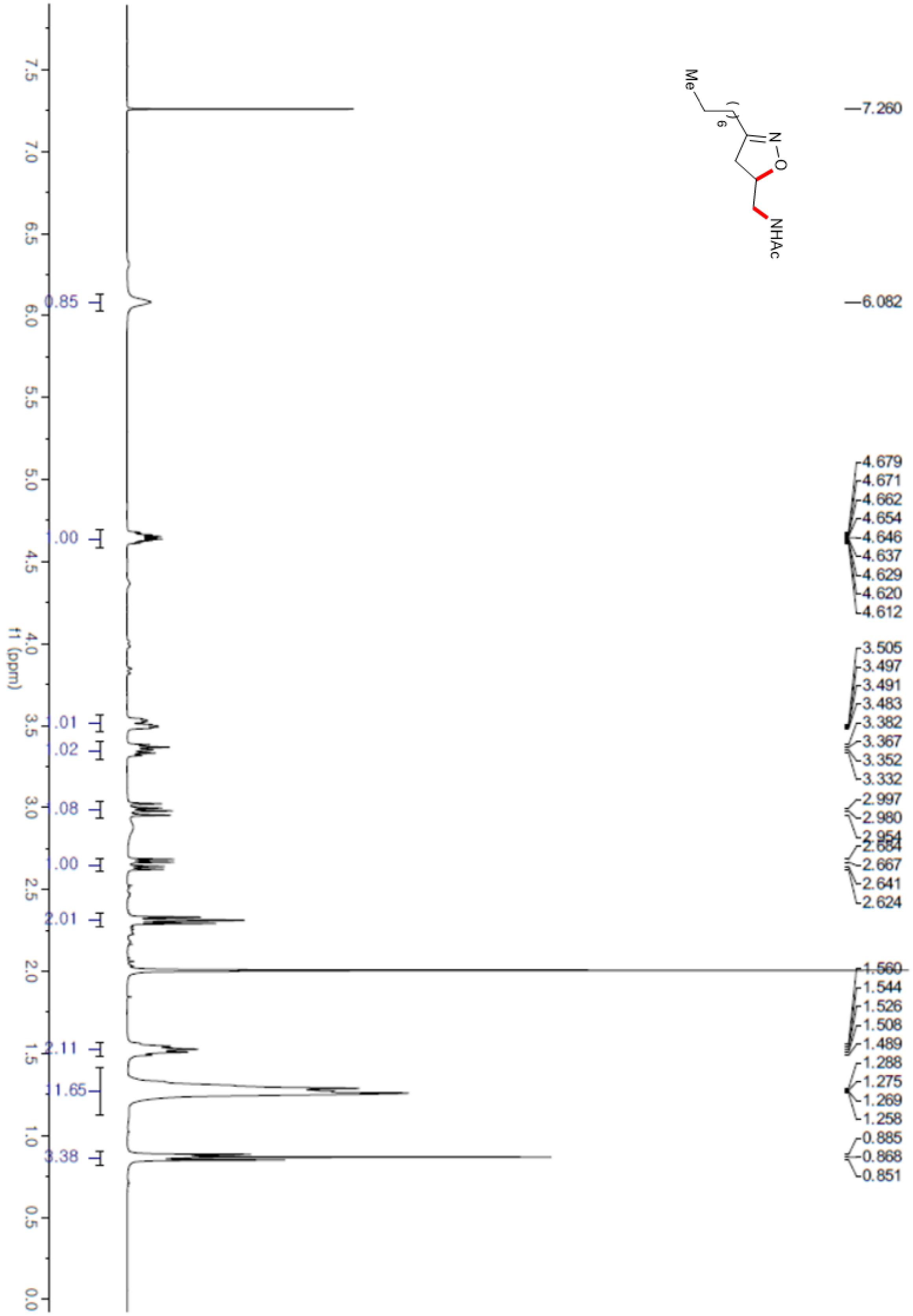

Figure S32. ^{13}C NMR ($CDCl_3$) of **3k**

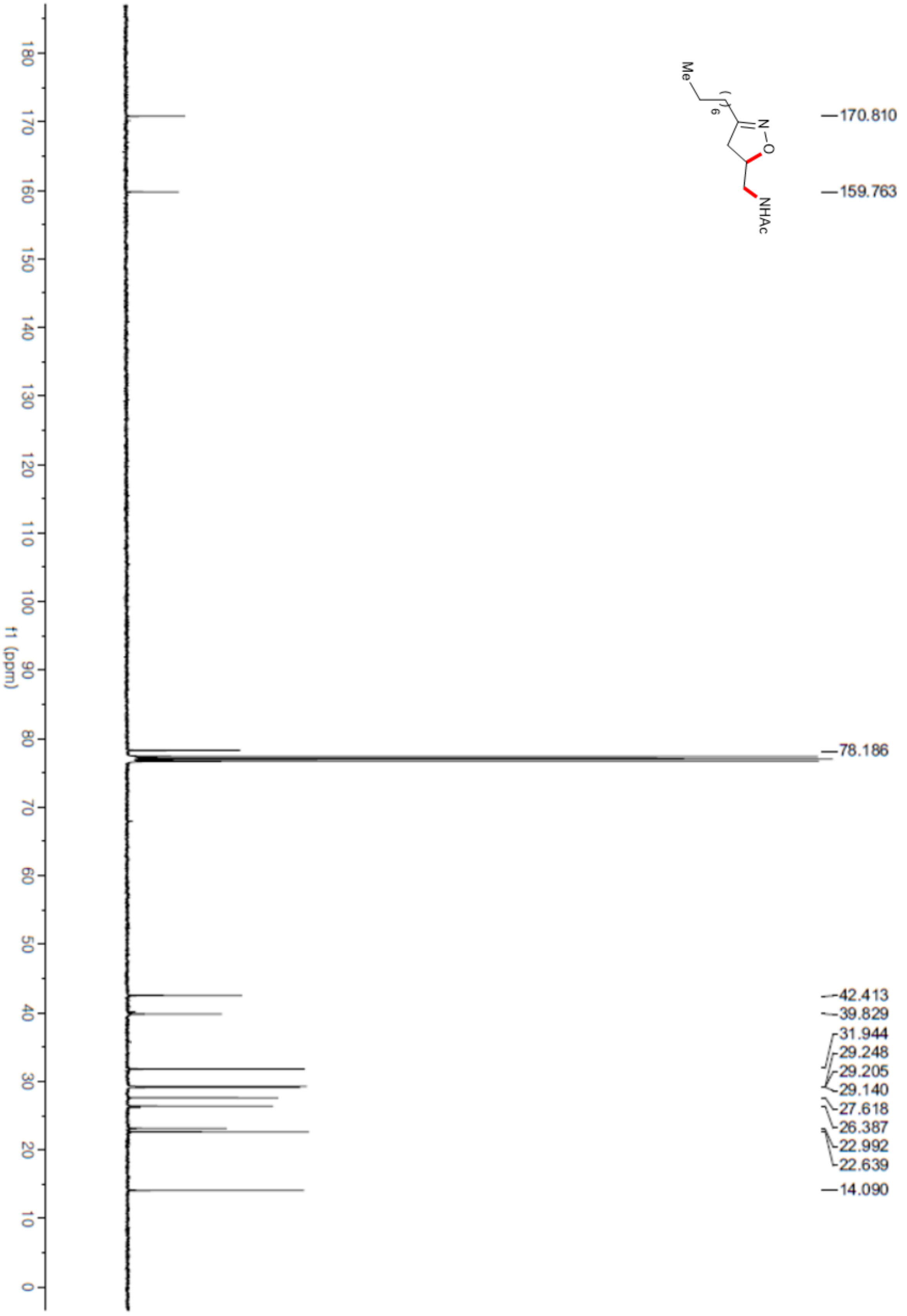

Figure S23. ^{1}H NMR ($CDCl_3$) of **3l**

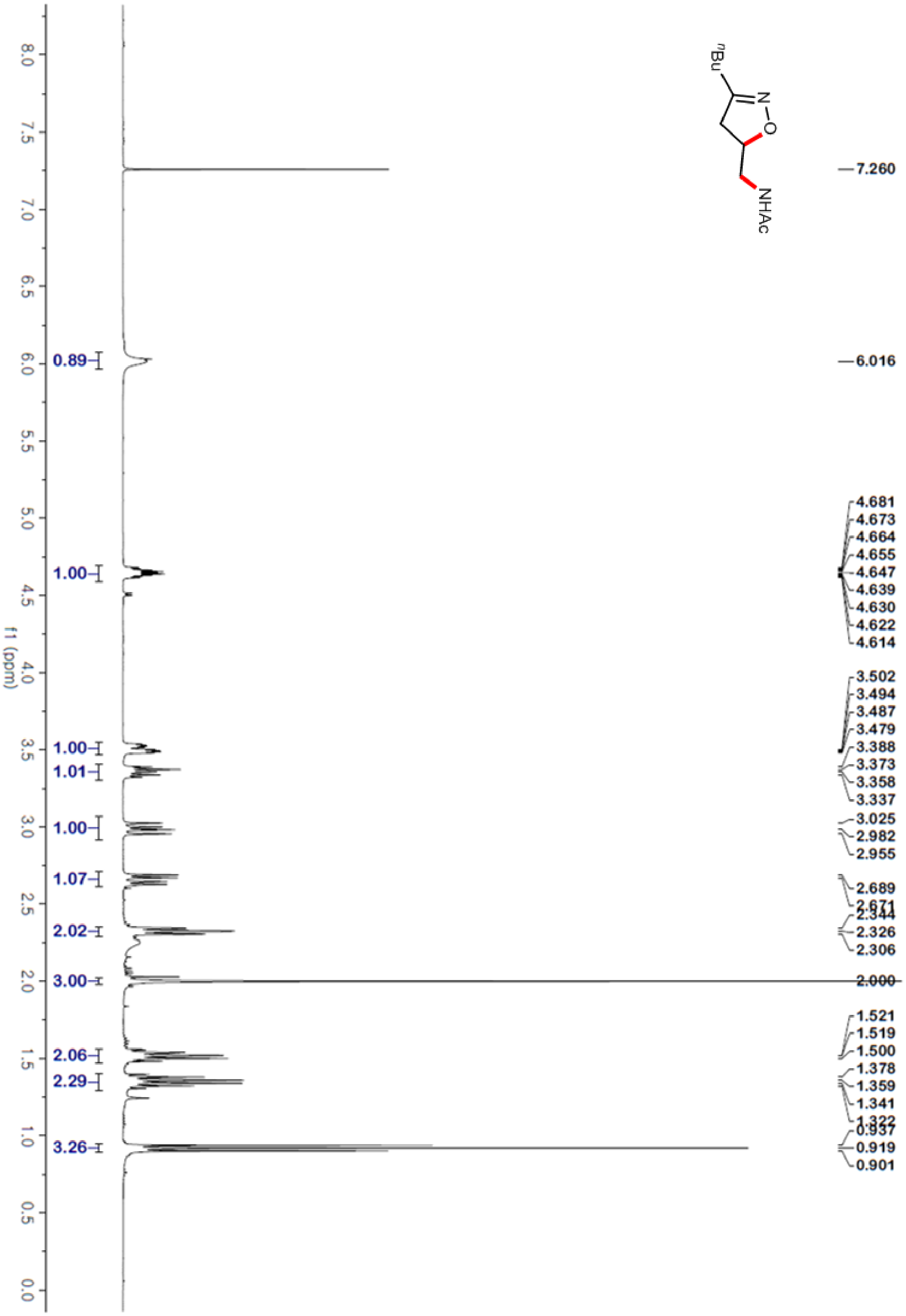

Figure S24. ^{13}C NMR ($CDCl_3$) of **3l**

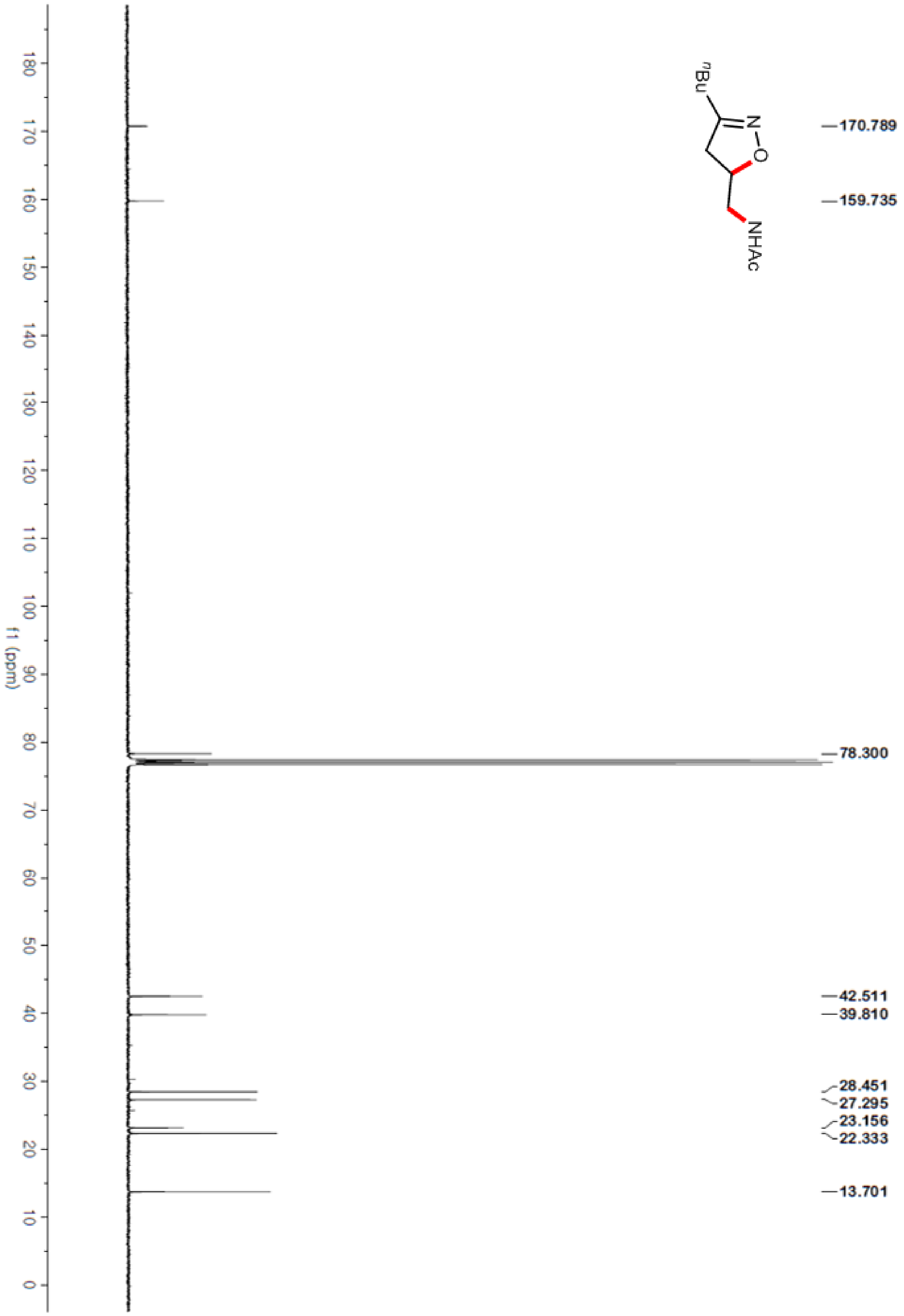

Figure S45. ^{1}H NMR ($CDCl_3$) of **3m**

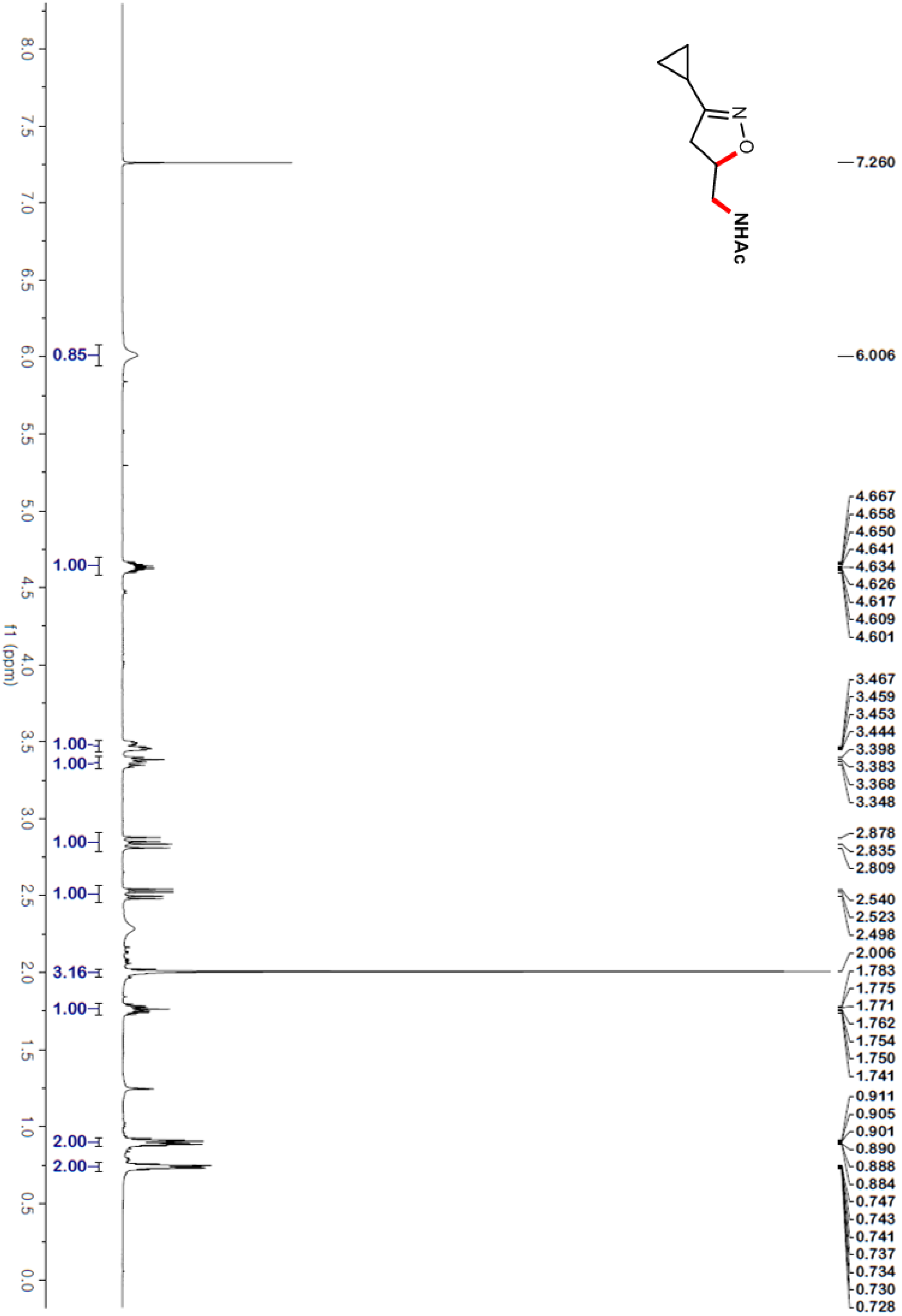

Figure S56. ^{13}C NMR ($CDCl_3$) of **3m**

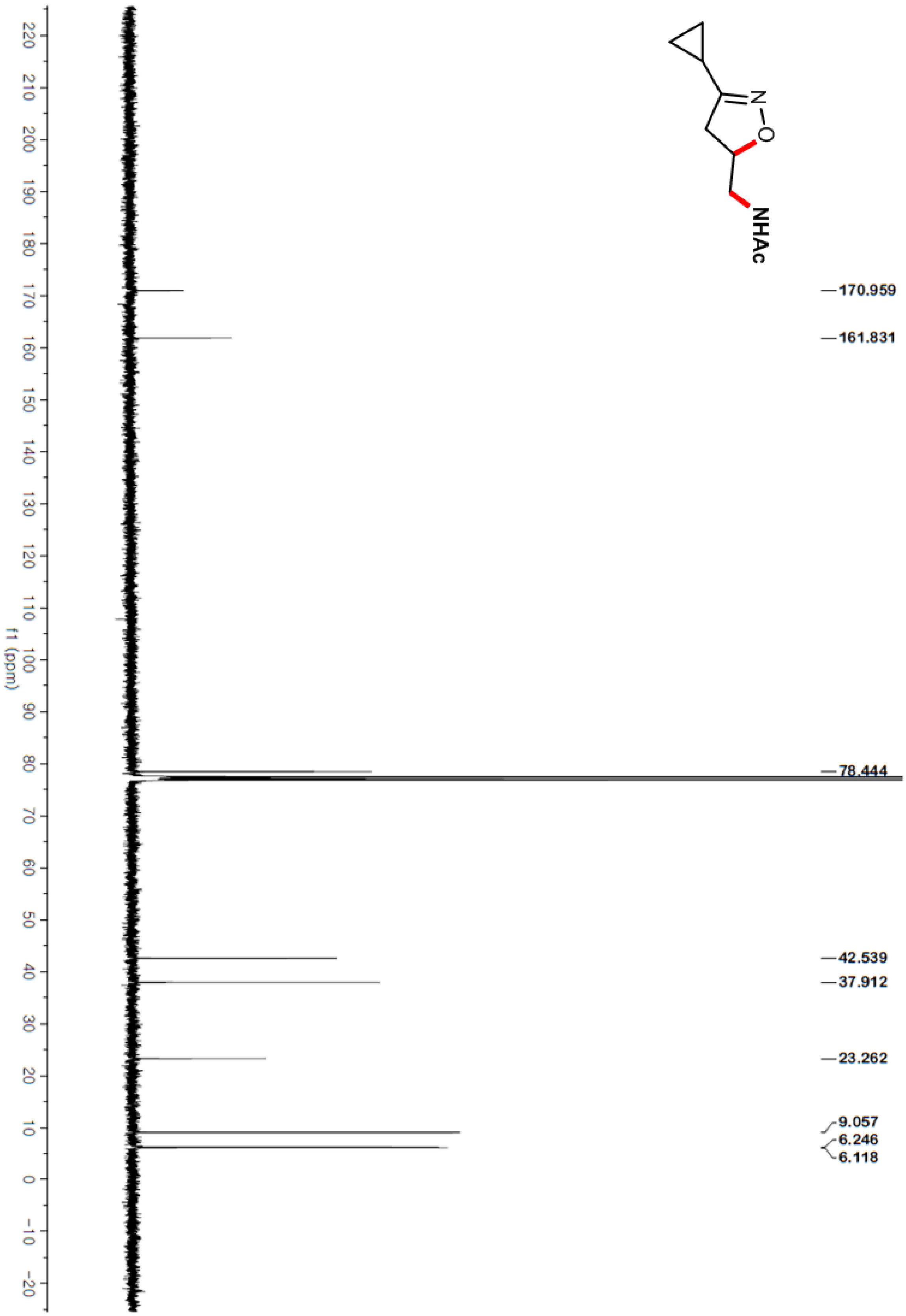

Figure S67. ^{1}H NMR ($CDCl_3$) of **5a**

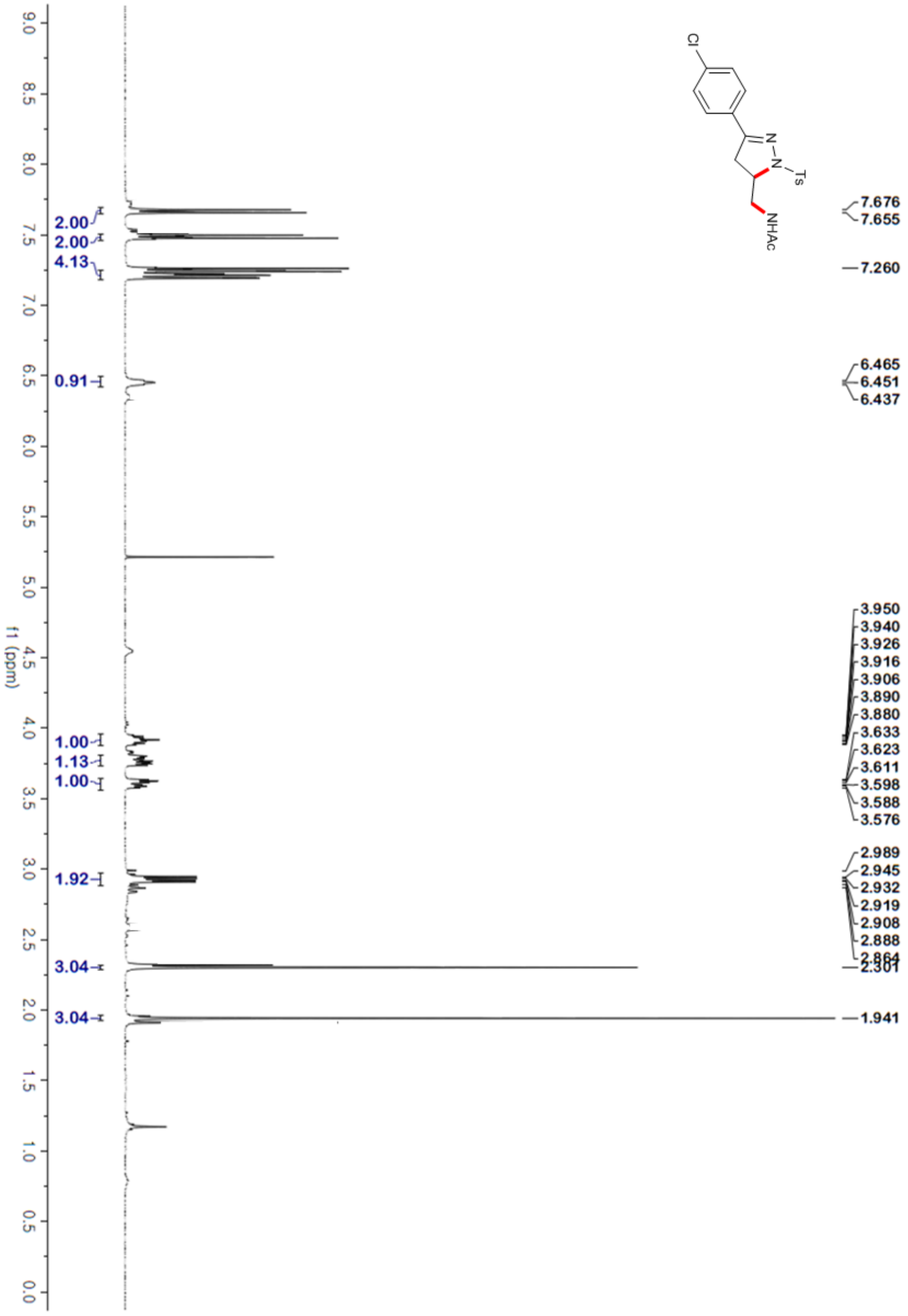

Figure S78. ^{13}C NMR ($CDCl_3$) of **5a**

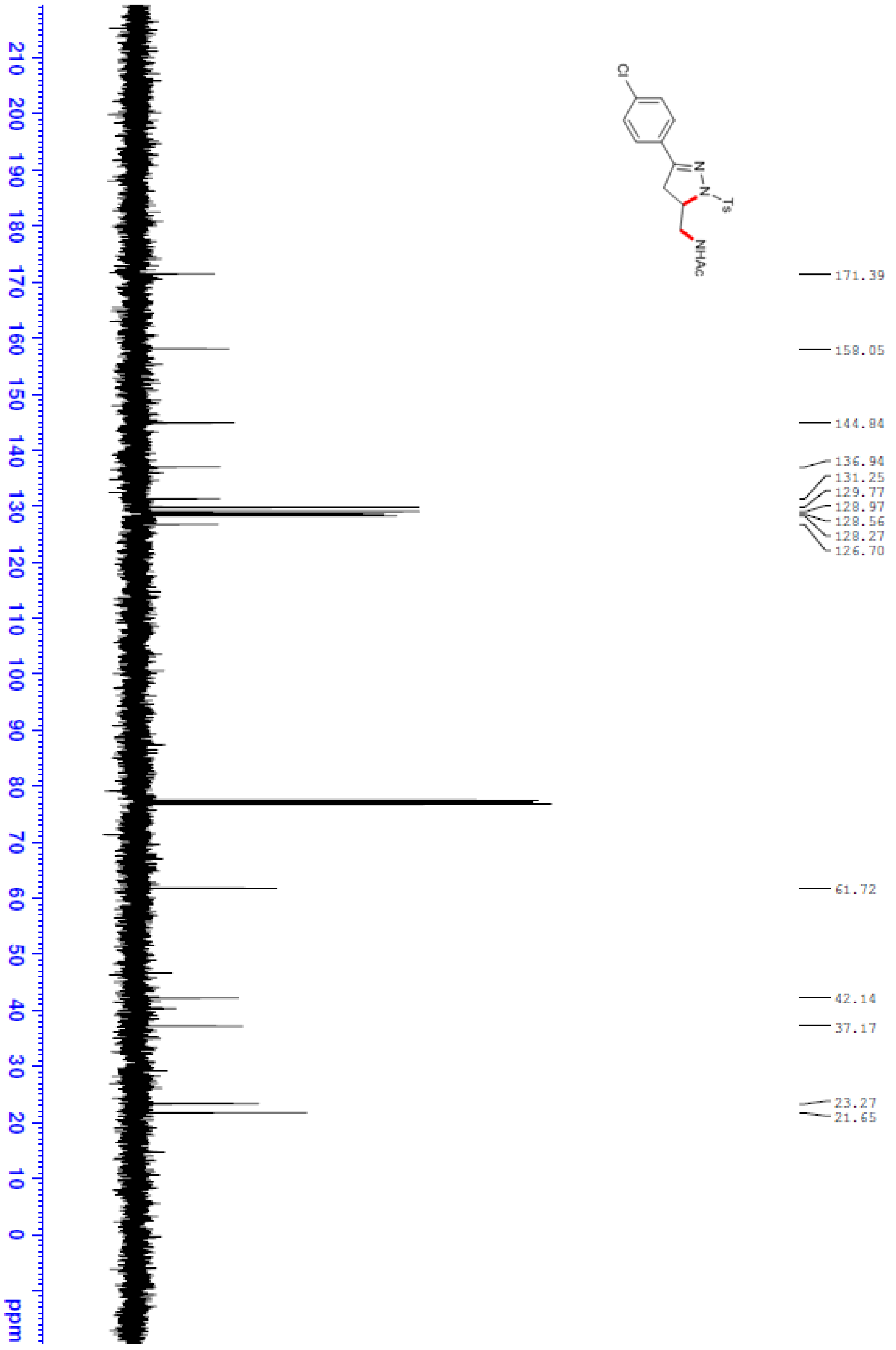

Figure S89. ^{1}H NMR ($CDCl_3$) of **5b**

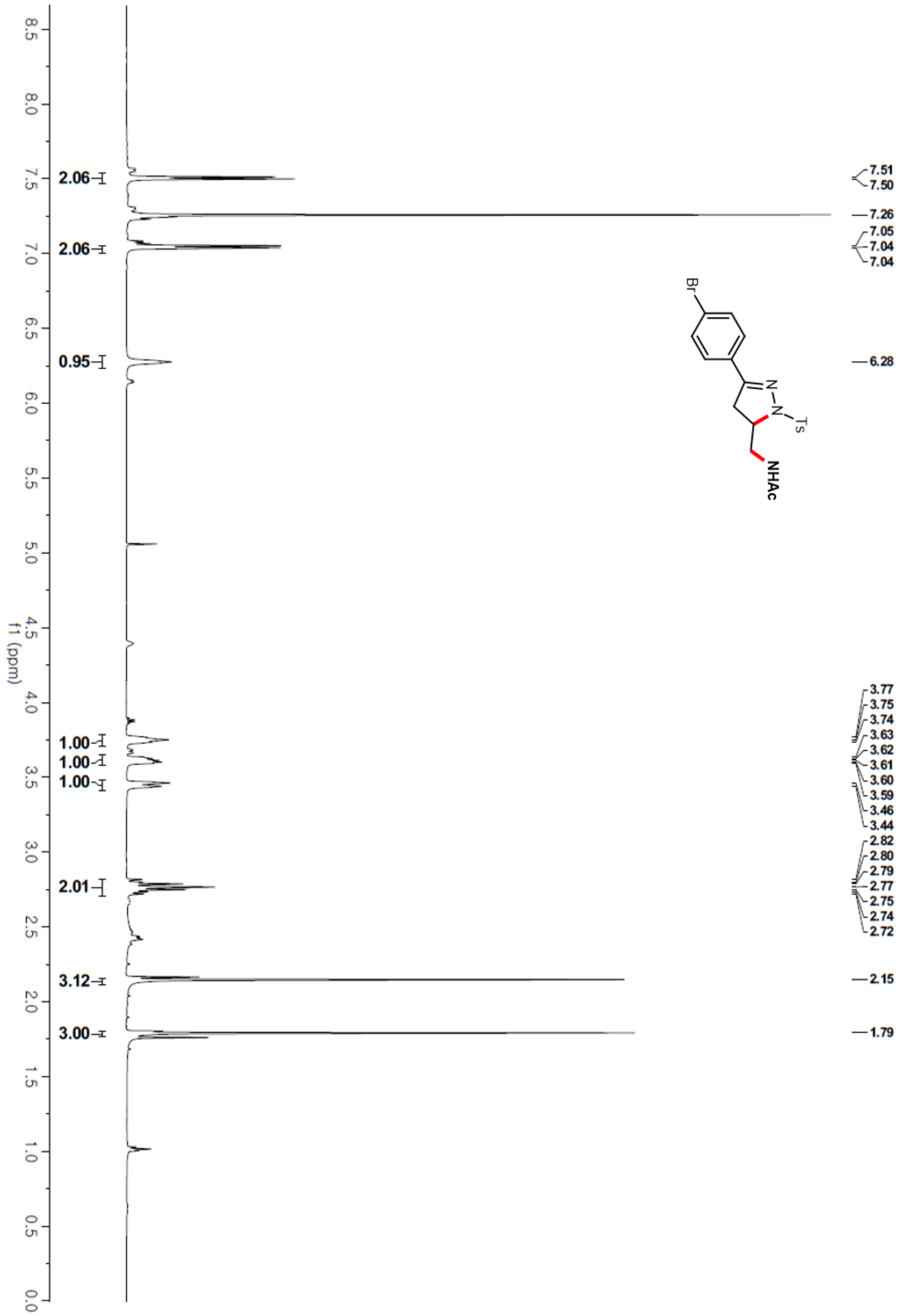

Figure S30. ^{13}C NMR ($CDCl_3$) of **5b**

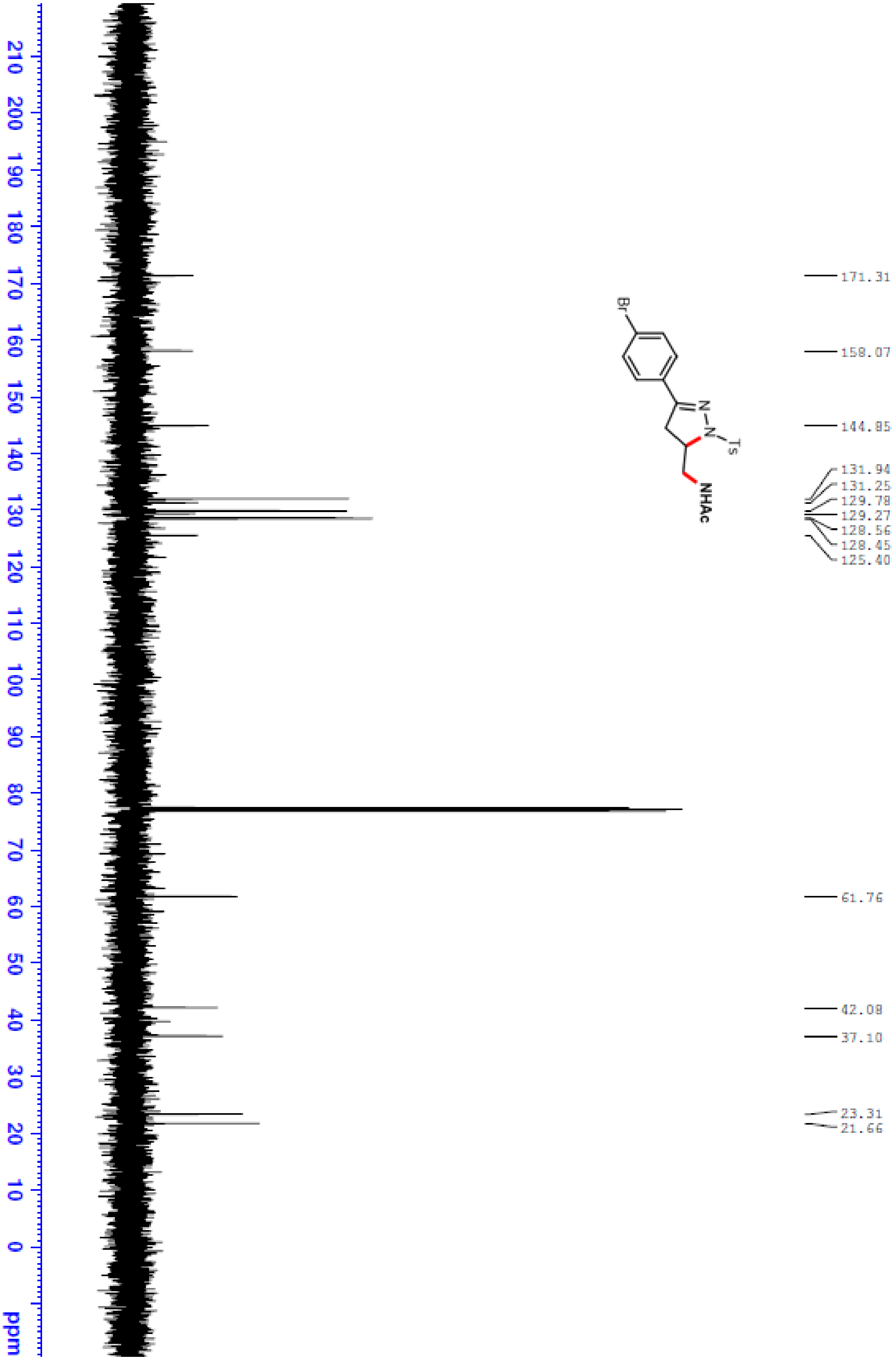

Figure S31. ^{1}H NMR ($CDCl_3$) of **5c**

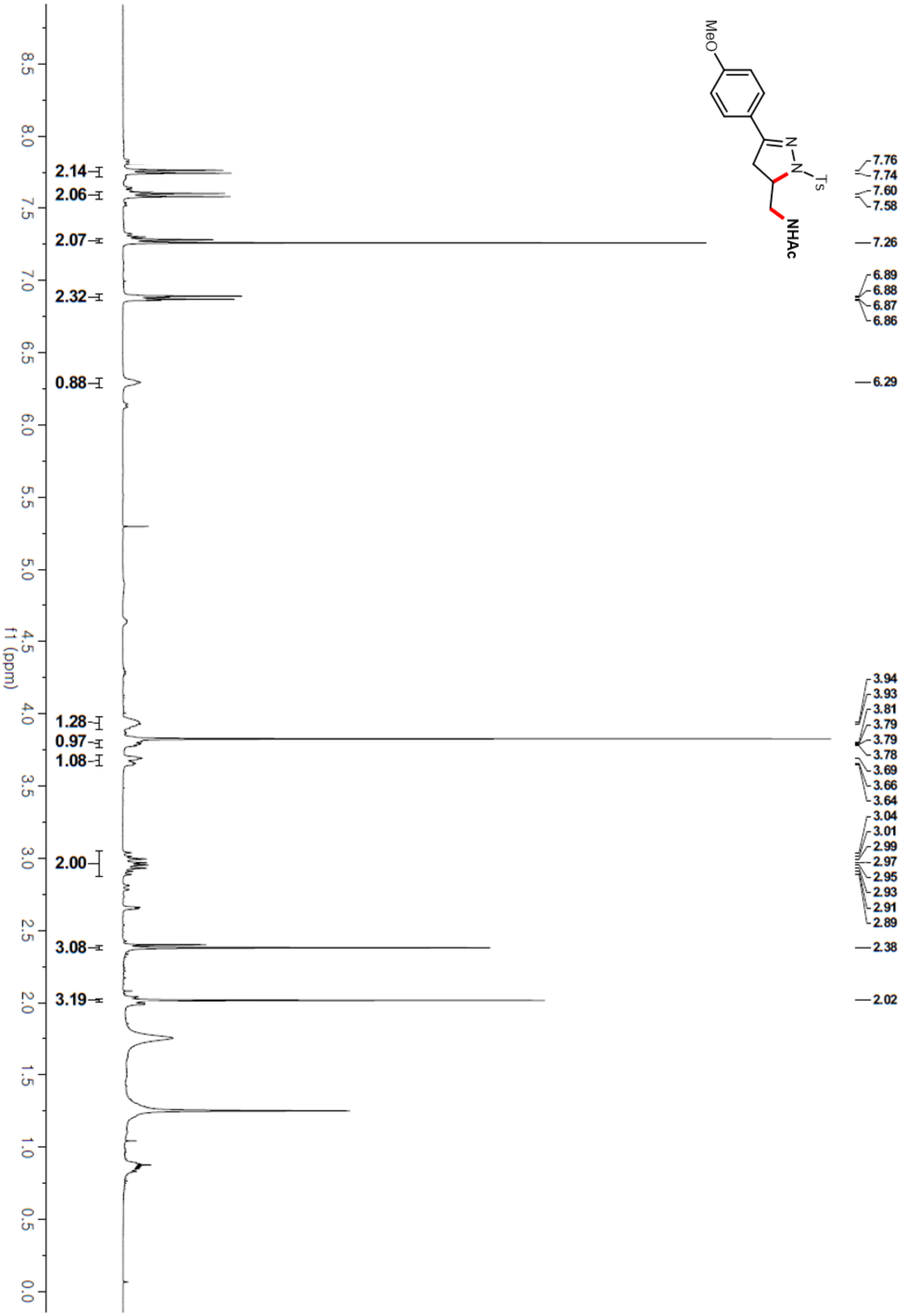

Figure S32. ^{13}C NMR ($CDCl_3$) of **5c**

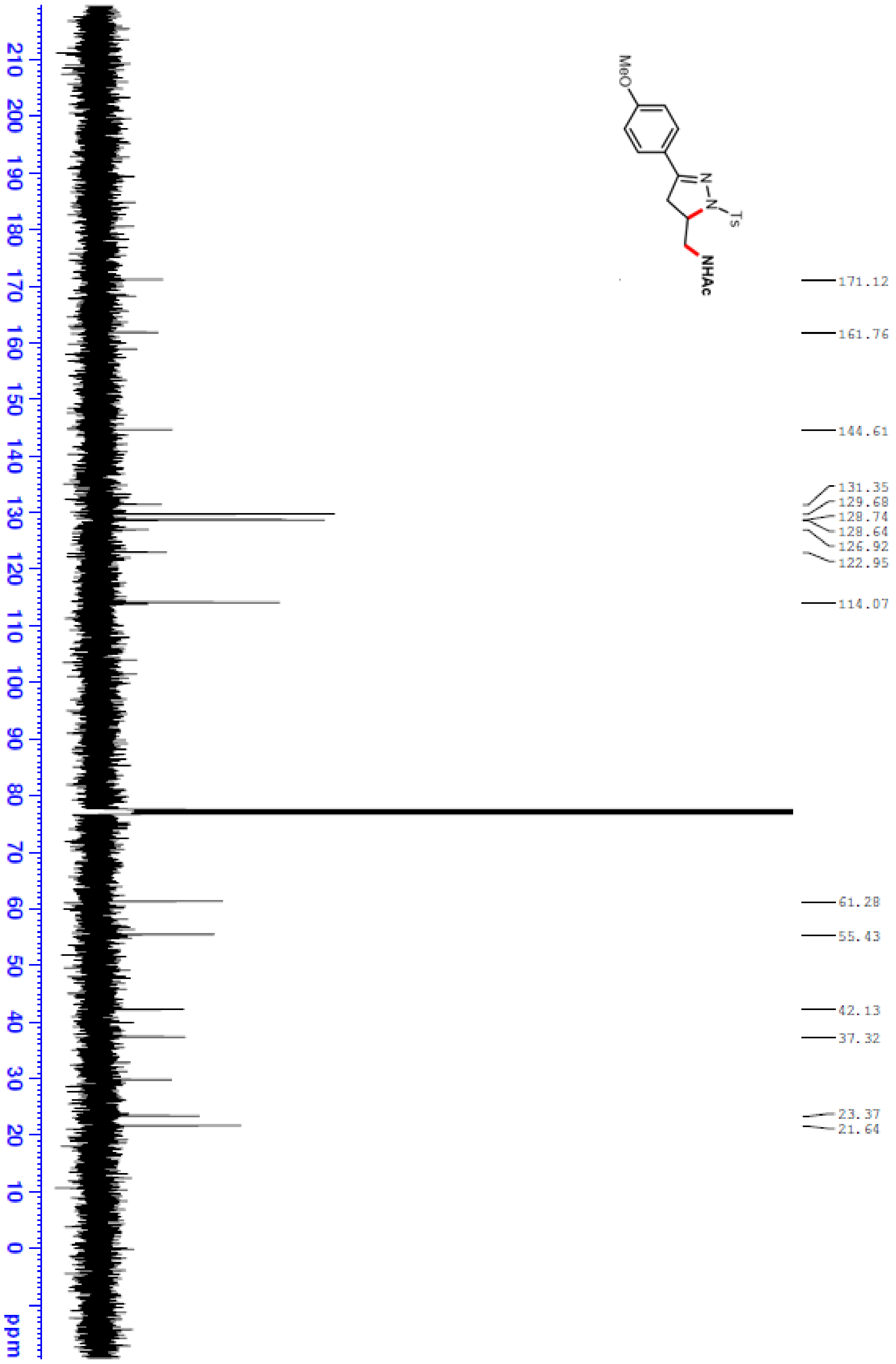

Figure S33. ^{1}H NMR ($CDCl_3$) of **5d**

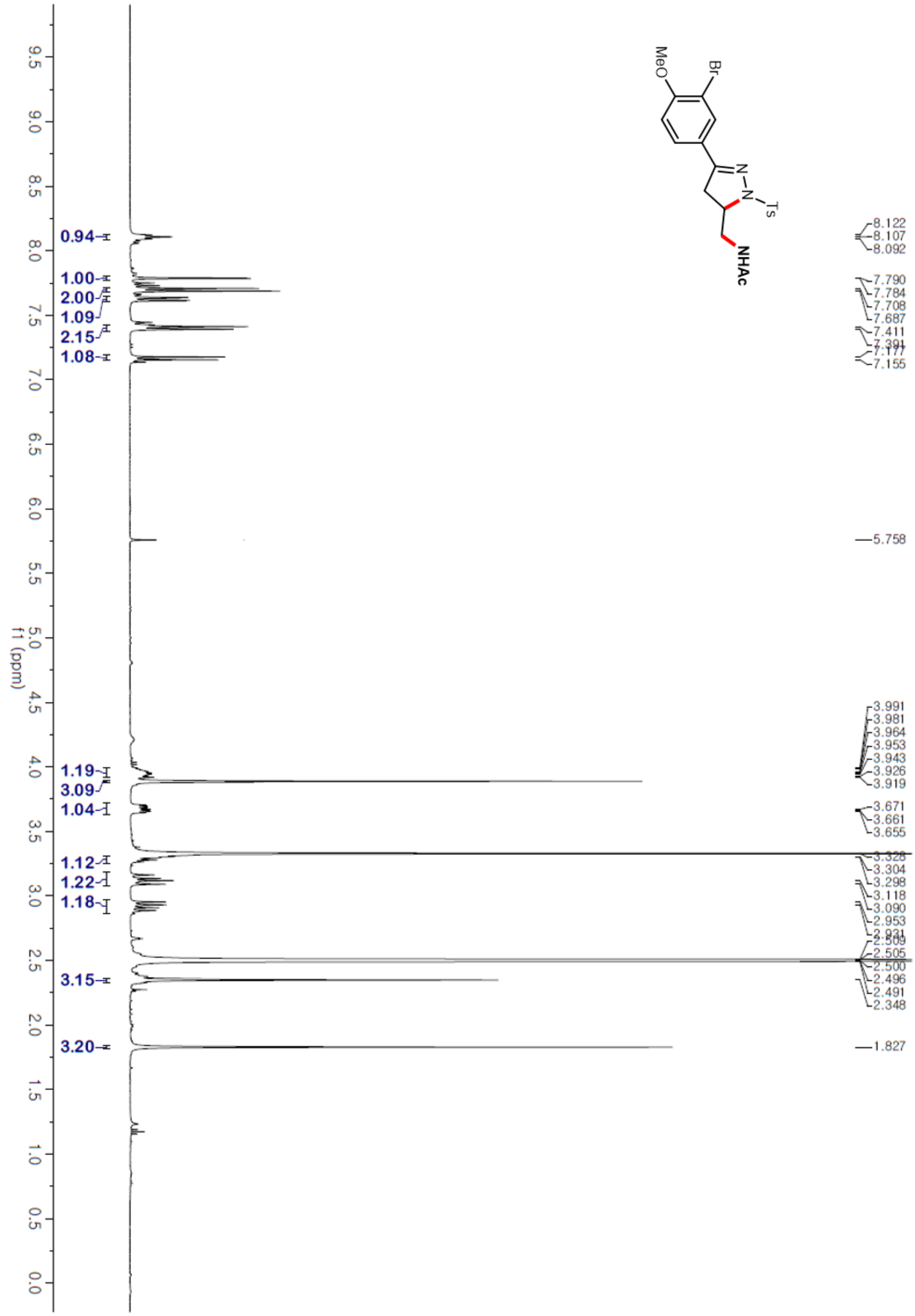

Figure S34. ^{13}C NMR ($CDCl_3$) of **5d**

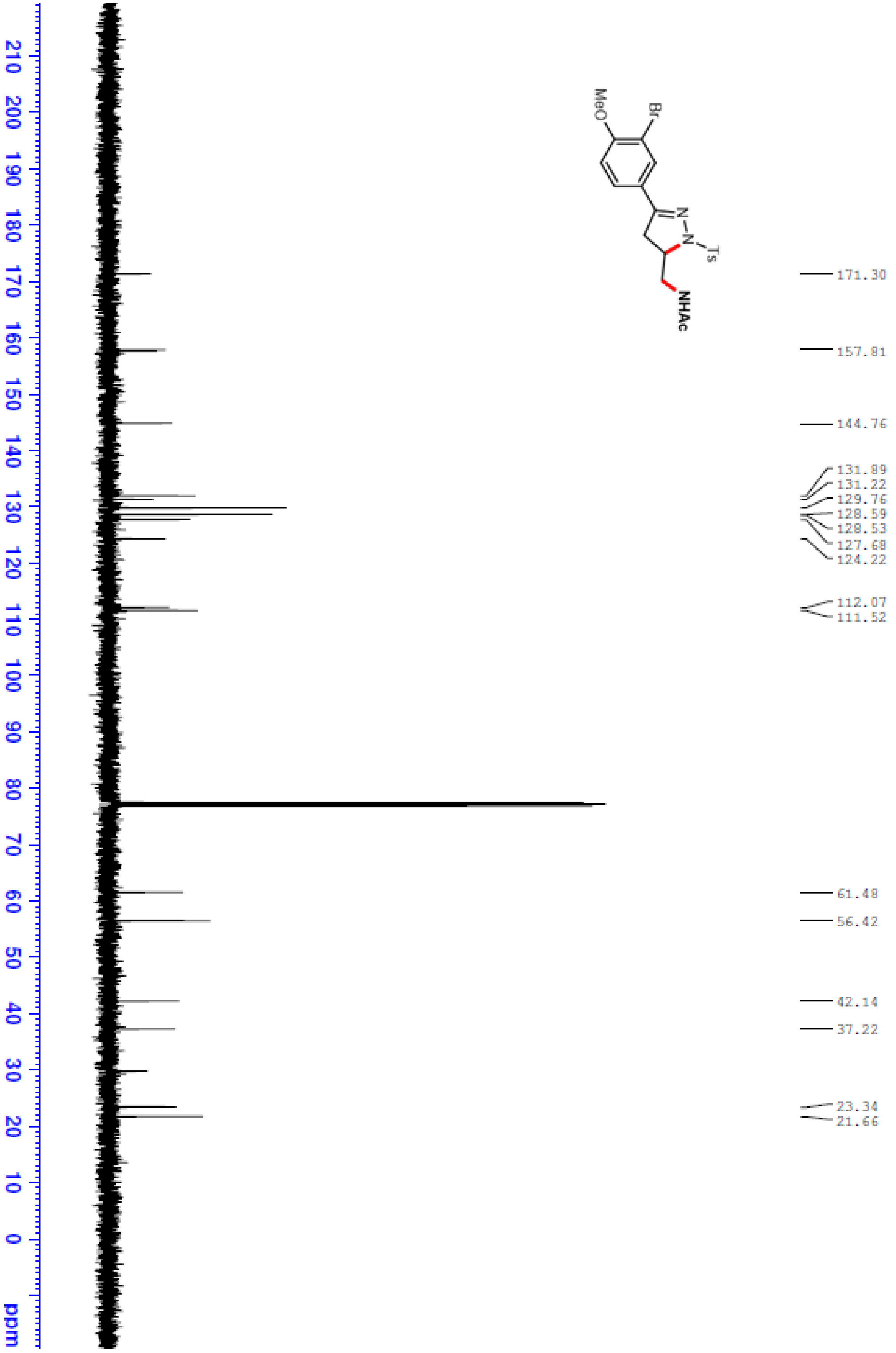

Figure S35. ^{1}H NMR ($CDCl_3$) of **5e**

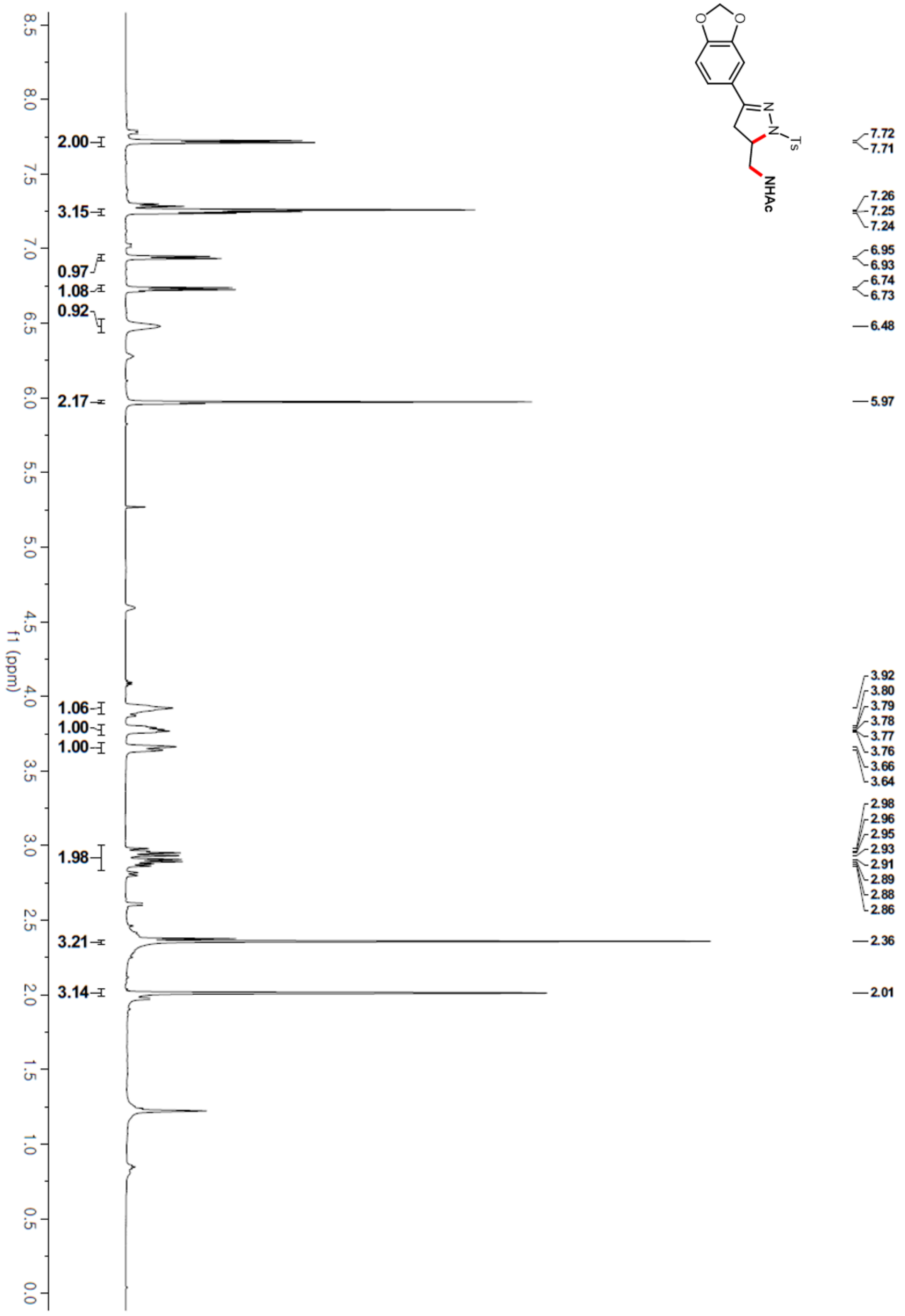

Figure S36. ^{13}C NMR ($CDCl_3$) of **5e**

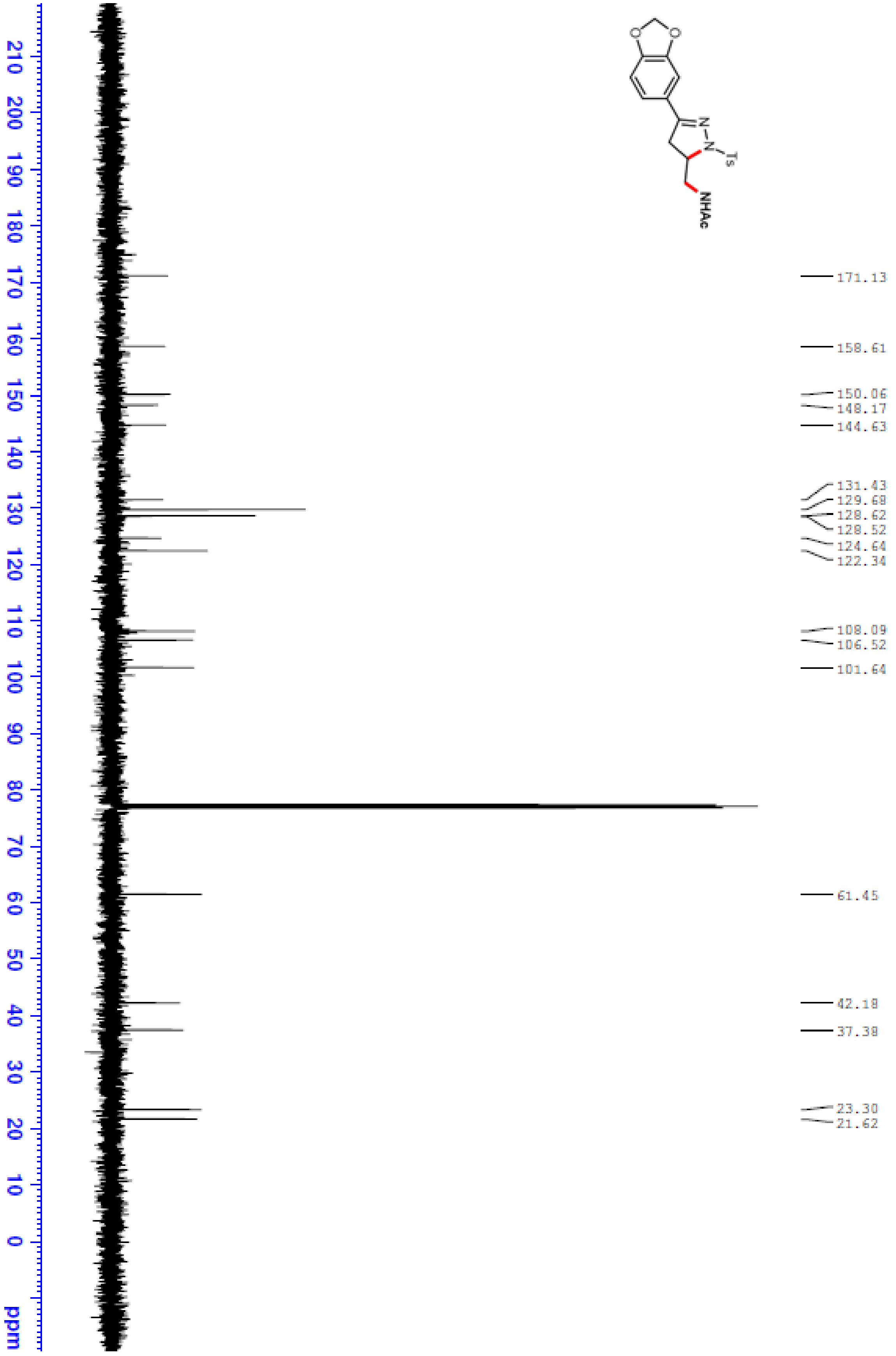

Figure S37. ^{1}H NMR ($CDCl_3$) of **5f**

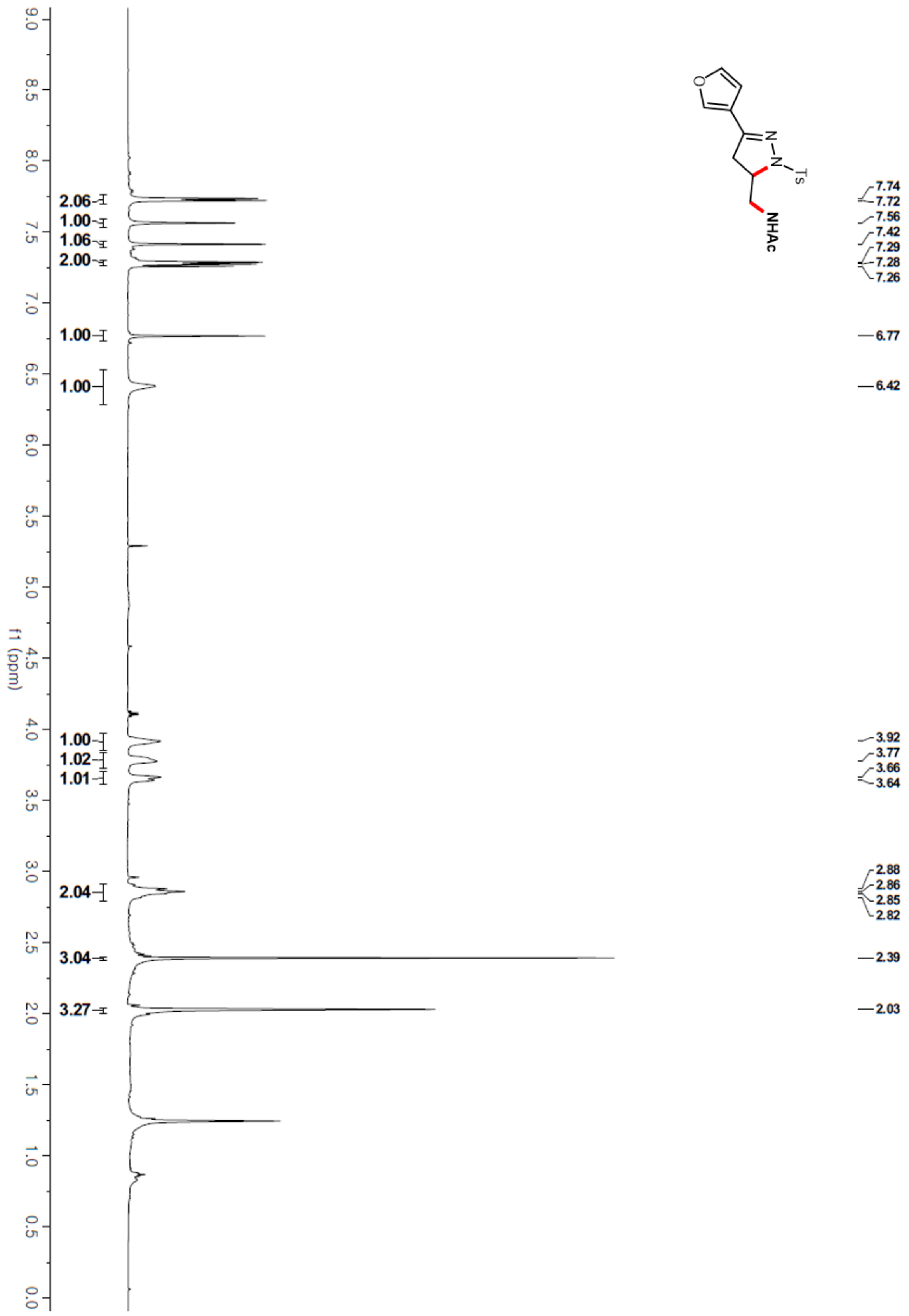

Figure S38. ^{13}C NMR ($CDCl_3$) of **5f**

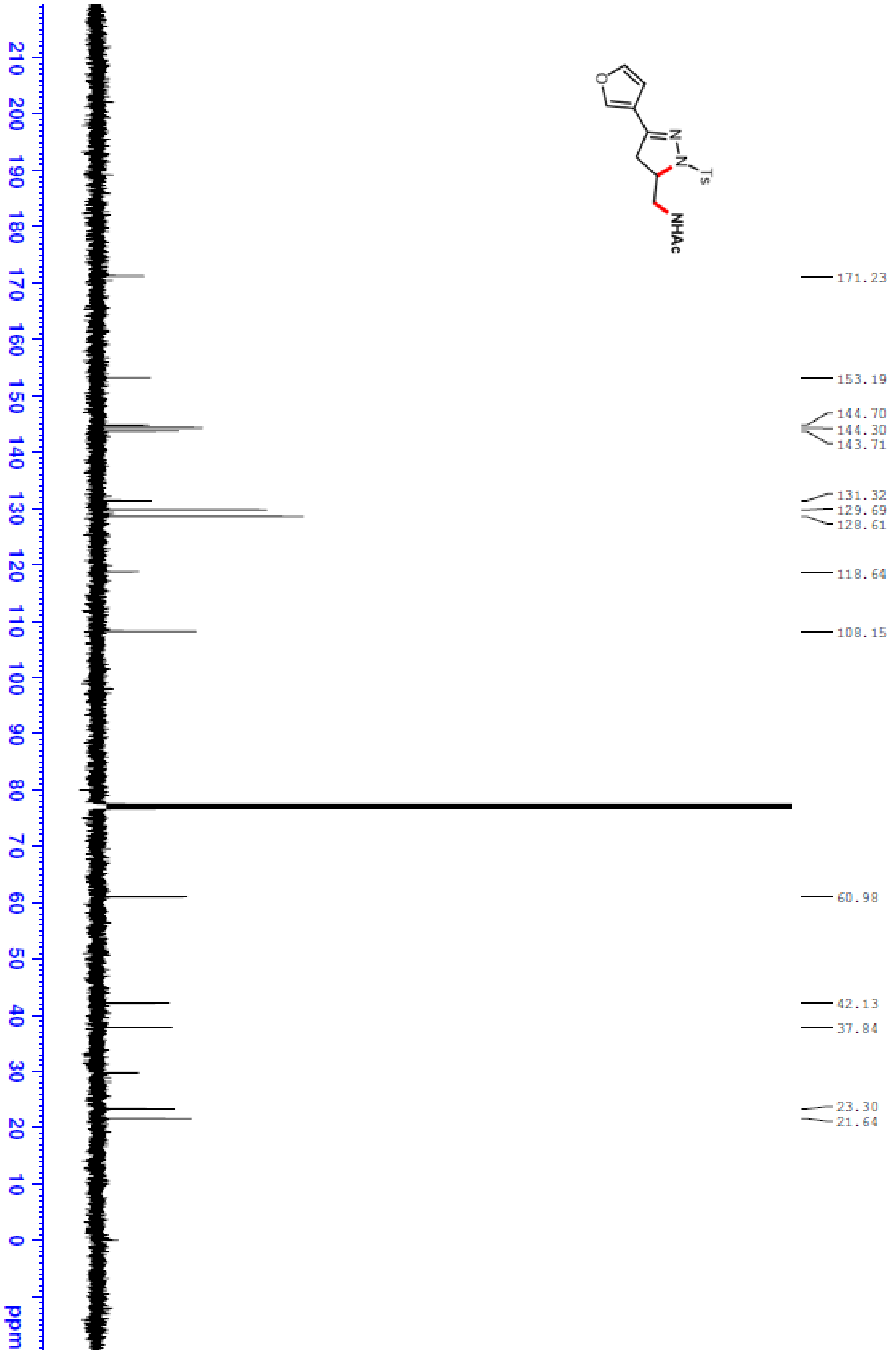

Figure S39. ^{1}H NMR ($CDCl_3$) of **5g**

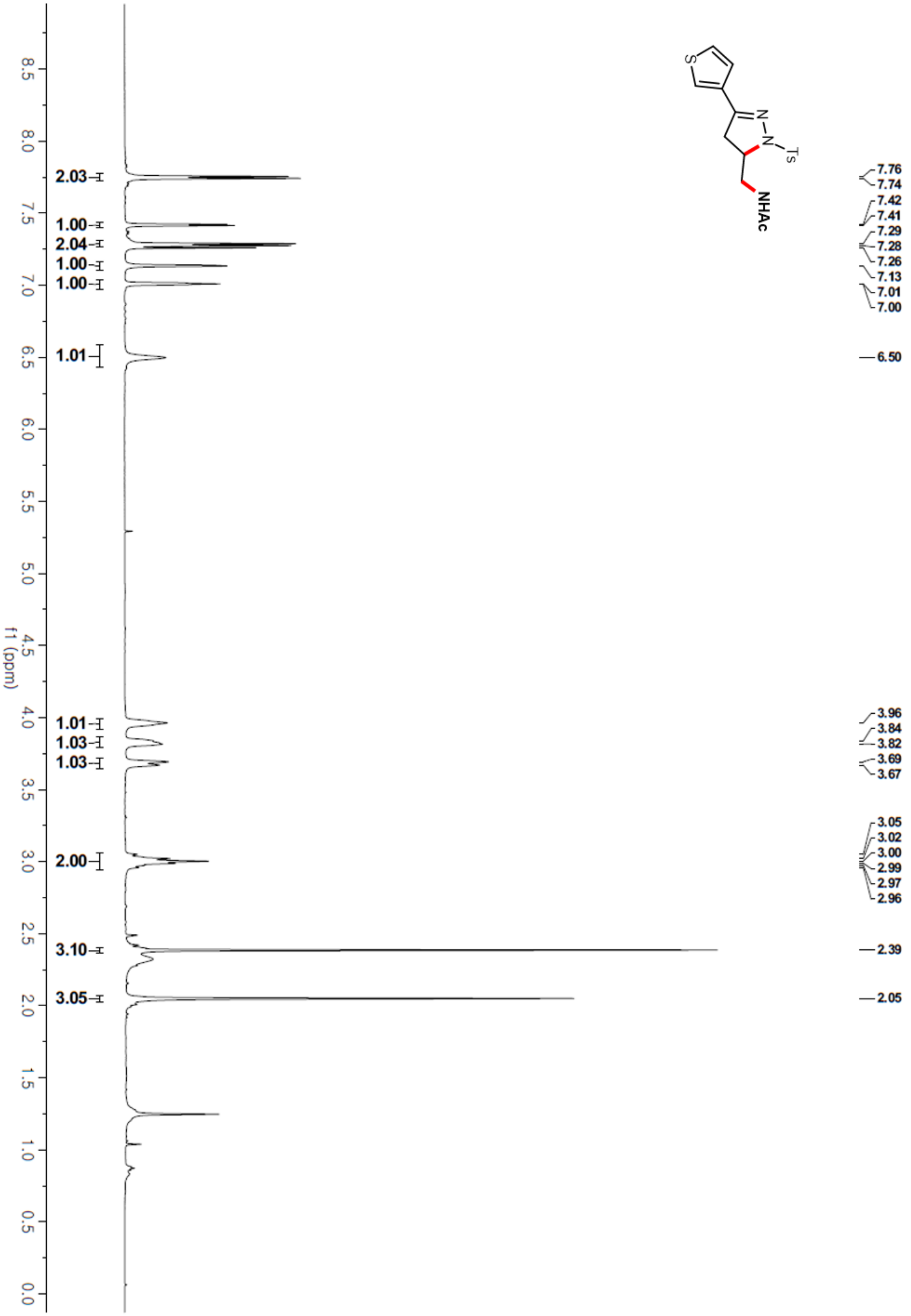

Figure S40. ^{1}H NMR ($CDCl_3$) of **5g**

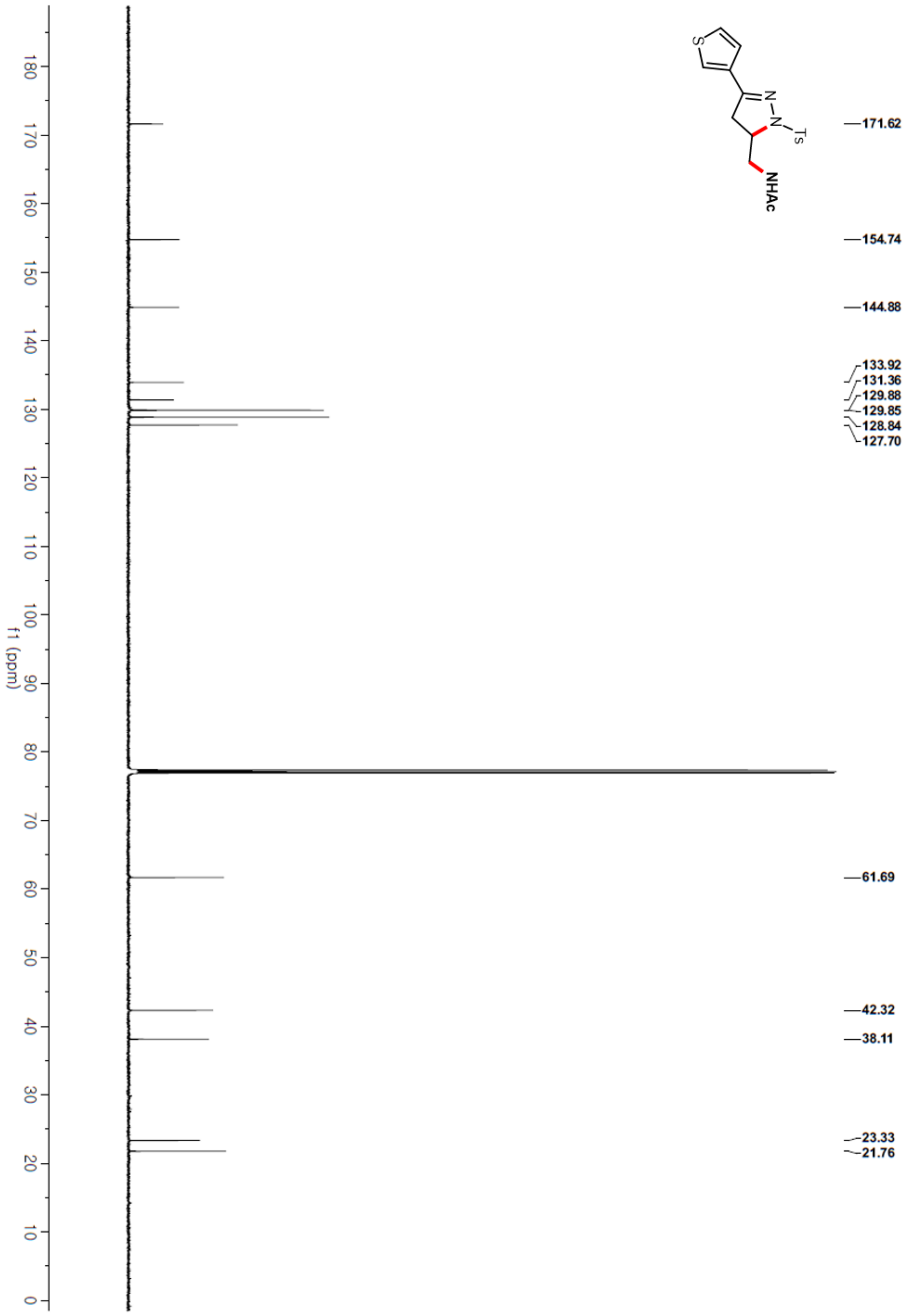

Figure S41. ^{1}H NMR ($CDCl_3$) of **5h**

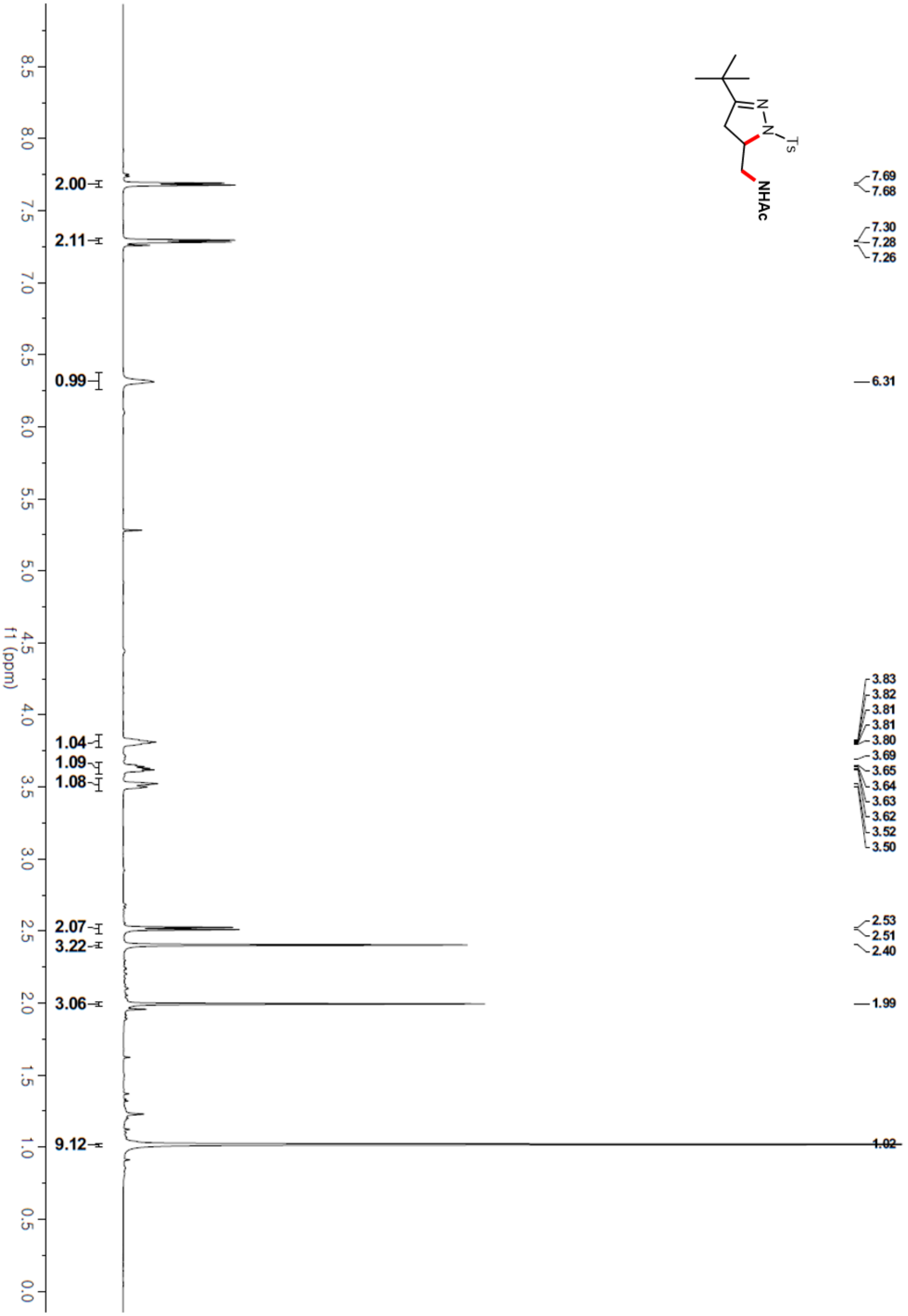

Figure S42. ^{13}C NMR ($CDCl_3$) of **5h**

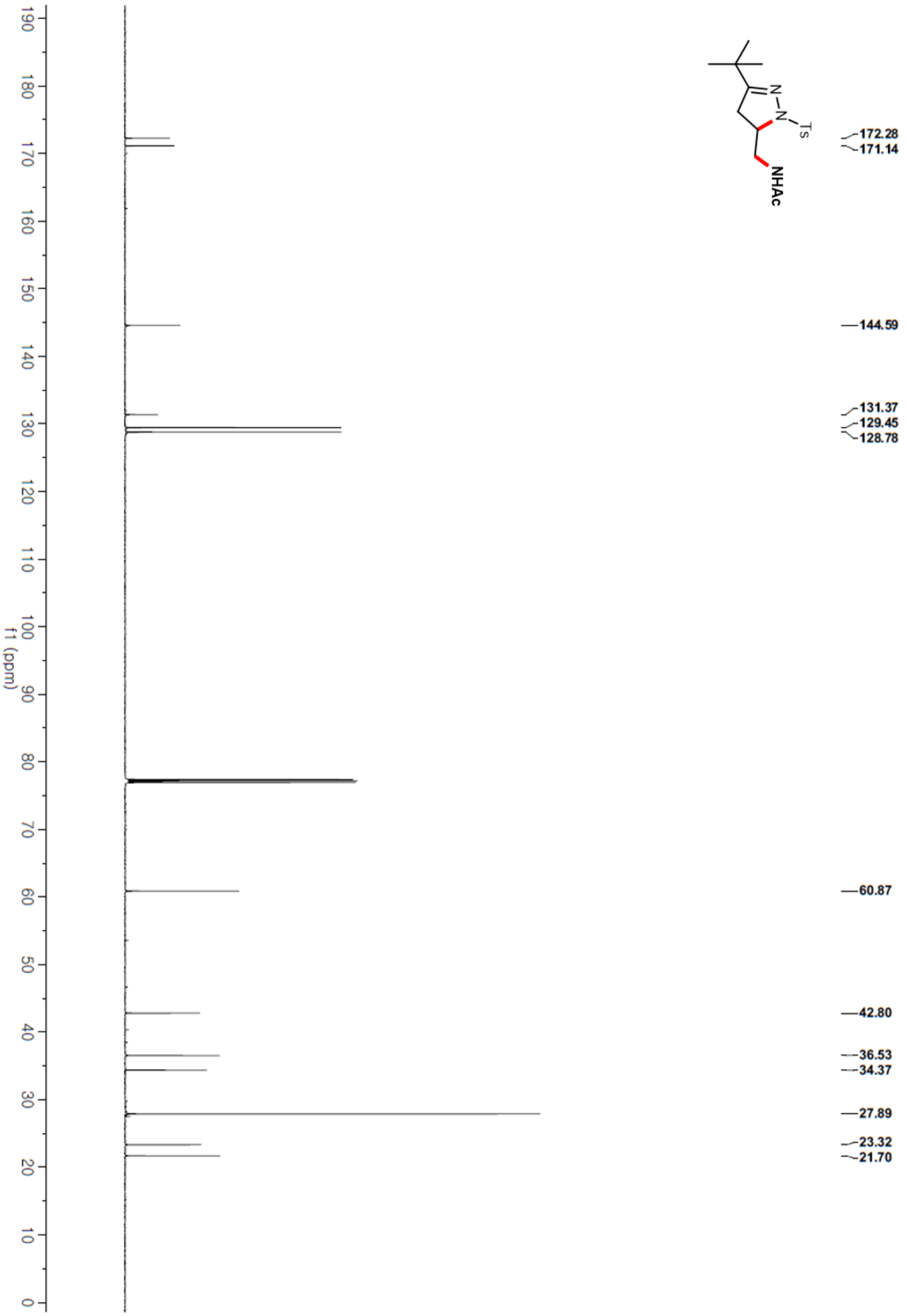

Figure S43. ^{1}H NMR ($CDCl_3$) of **5i**

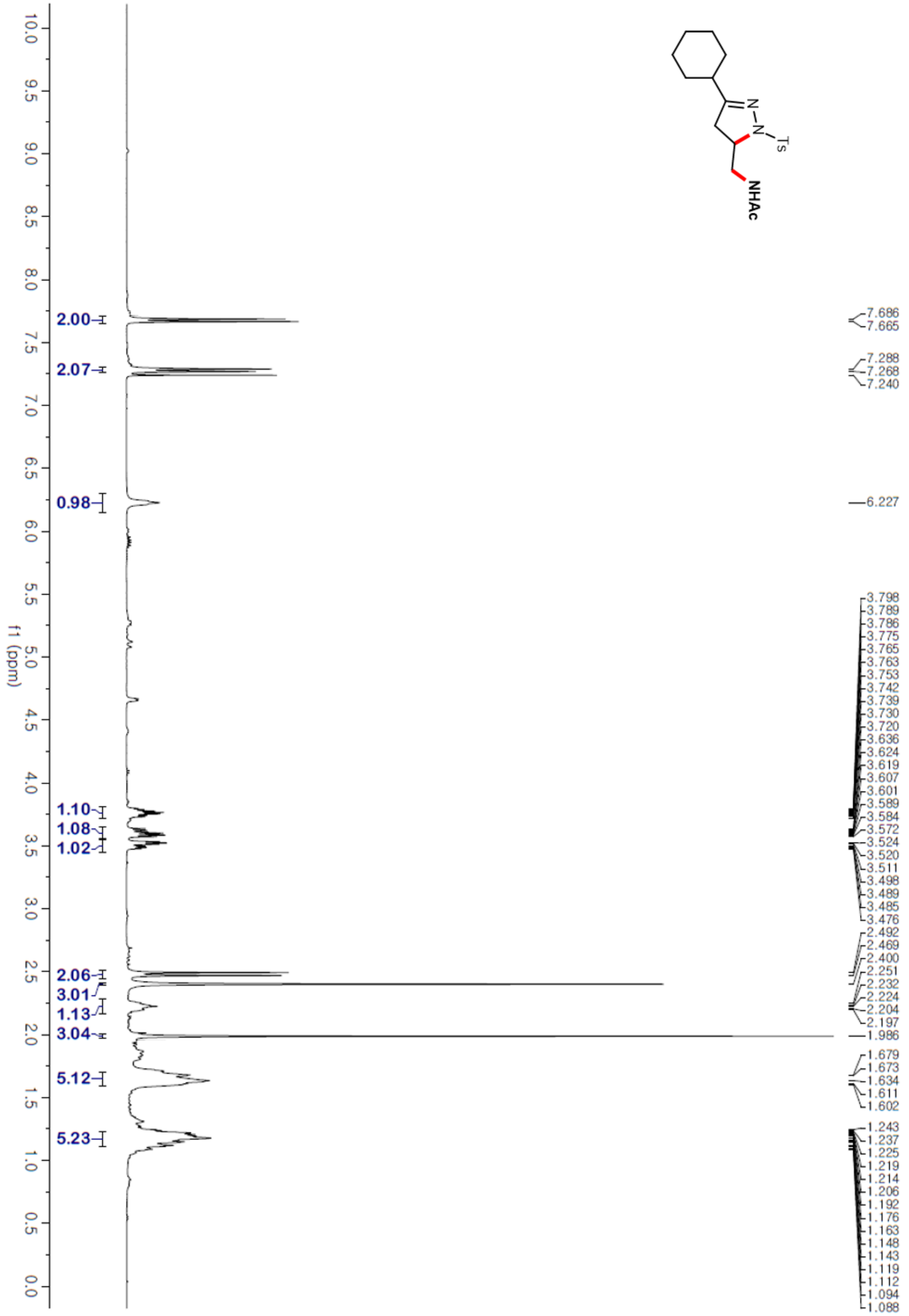

Figure S44. ^{13}C NMR ($CDCl_3$) of **5i**

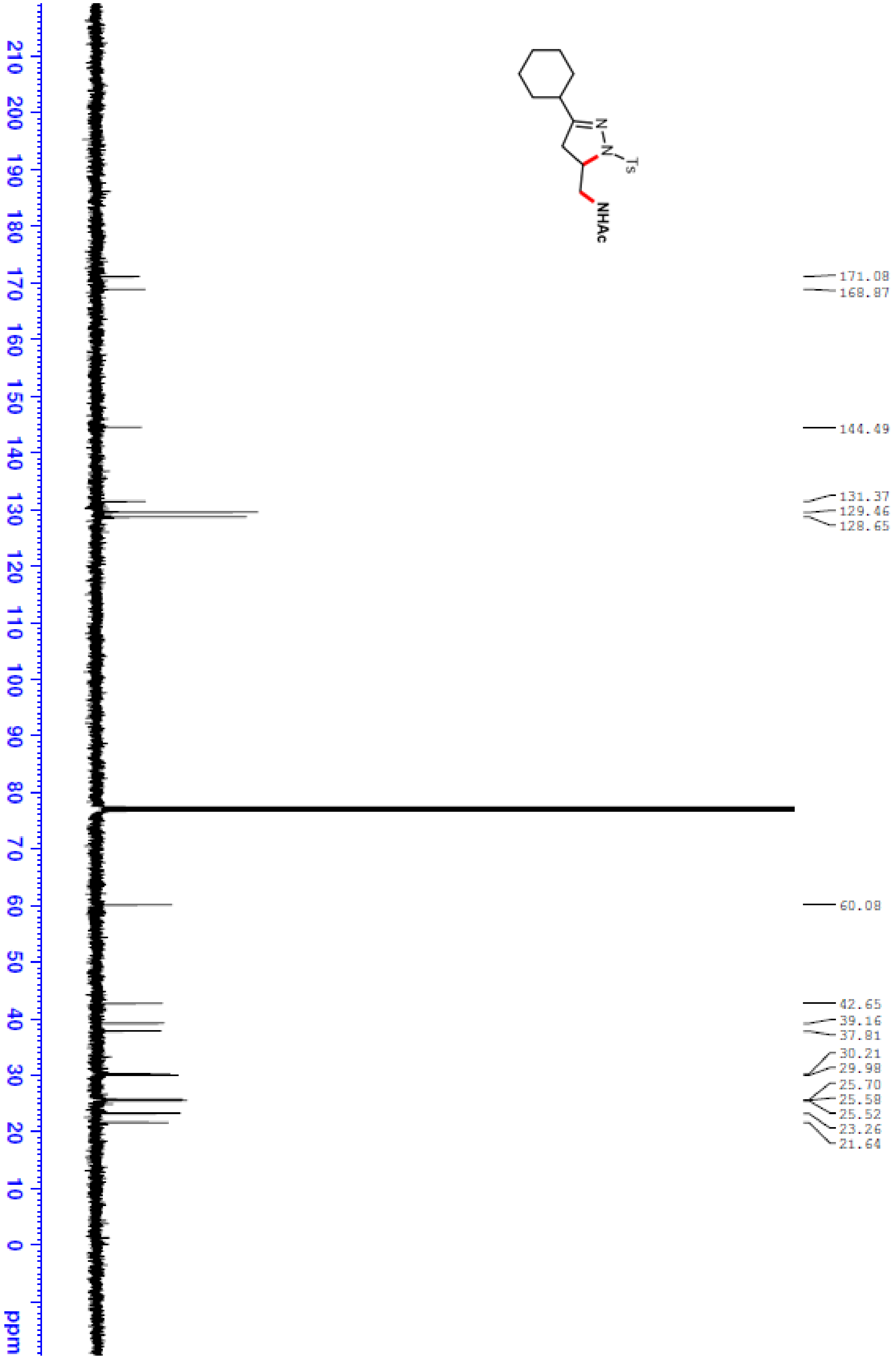

Supplement: File 1 — Experimental procedures, characterization data, and copies of 1H and 13C NMR spectra. [file Beilstein_J_Org_Chem-14-1028-s001.pdf]
